# Mapping the methodological diversity of published drug discontinuation studies—a scoping review of study topics, objectives, and designs

**DOI:** 10.1186/s13063-023-07105-6

**Published:** 2023-01-26

**Authors:** Nina Grede, Katrin Kuss, Ina Staudt, Norbert Donner-Banzhoff, Annika Viniol

**Affiliations:** Department of General Medicine, Preventive and Rehabilitation Medicine, University of Marburg, Karl-Von-Frisch Str. 4, 35043 Marburg, Germany

**Keywords:** Drug discontinuation, Withdrawal, Scoping review, Polypharmacy, PRISMA-ScR

## Abstract

**Background:**

Trials evaluating drug discontinuation (drug discontinuation trials, DDTs) show a broad methodological spectrum. There are several specific methodological aspects in drug discontinuation trials (e.g., determination of research question; configuration of intervention; definition of outcomes). To verify this specifies, we did a scoping review about the study designs of drug discontinuation trials.

**Methods:**

A systematic literature search in Medline (PubMed), The Cochrane Library, EMBASE, CINAHL, Web of Science, and PsycINFO was performed. In a two-step selection process, we identified DDTs, which evaluate the discontinuation of one or more long-term medication as the investigated intervention, by two independent reviewers. Besides bibliographic data, we extracted several parameters to describe the used study design of the included DDTs: motivation for DDT, initially treatment aim of the discontinued medication, study design, methods of discontinuation, follow-up times, number of study participants, and outcome parameter.

**Results:**

Out of 12,132 records, we included 581 DDTs. The most common motivation for doing a DDT were expected side effects (48.8%), the motivation of proving the efficacy of medication (21.6%), or doubts on the expected benefit of the used medication (13.8%). The majority of the discontinued medication was initially prescribed to improve the prognosis of a chronic disease (60.4%) or to relieve symptoms (31%). The study designs of the trials showed a broad methodological spectrum. The minority of the drug discontinuation trials were randomized controlled trials (34%).

**Conclusion:**

The results of this scoping review illustrates the need for an evidence-based methodological standard for planning and conducting drug discontinuation trials.

**Supplementary Information:**

The online version contains supplementary material available at 10.1186/s13063-023-07105-6.

## Background

The design of the classical drug trials has evolved over at least 70 years [1]. A large number of systematic reviews and discussion papers have investigated the appropriateness of their study designs, outcomes, statistical analysis, and reporting.

Design and methodology of studies evaluating the discontinuation of long-term medication are much less established. Clinical practice guidelines focus much more on starting medications than on stopping them [2].

For patients suffering from chronic disease, a large number of drugs is available to alleviate symptoms, to prevent relapse or to improve prognosis. Regulatory authorities and guideline panels require sufficient documentation of drug efficacy and safety before approval and/or recommendation.

As a result, most treatments have been evaluated by appropriately designed studies, such as randomized controlled trials (RCTs).

Due to continuous improvement of medical technologies, even patients with severe chronic diseases are experiencing a notable prolongation of life [3].

Patients suffering from more than one chronic condition are often confronted with many medications prescribed for their health problem(s). Each of these may be perfectly justified. In combination, however, they increase treatment burden and the risk of side-effects and interactions [4, 5]. Against this, background clinicians are encouraged to critically evaluate long-term medication and to stop it if appropriate [6].

Clinicians attempting drug withdrawal are acting on the basis of substantially weaker evidence in comparison to starting treatment [7]. Deprescribing is problematic only in certain clinical contexts. If the effect of a drug can be evaluated by immediate and reliable feedback (e.g., improvement in symptoms), clinicians institute and stop treatment by mostly informal *n* = 1-trials. Anti-Parkinson drugs or isosorbide-dinitrate for angina are common examples. In this case, individual experience trumps clinical trial evidence. The individual benefit of disease-modifying drugs, however, is difficult to evaluate. Their effects can be experienced only in the future. Moreover, clinical studies with clinically relevant outcomes show them to be associated with treatment (exposure) only in a probabilistic manner. Evidence derived from reliable clinical trials is thus urgently needed to inform deprescribing decisions especially for drugs impacting on long-term prognosis.

It is not new to conduct discontinuation studies. But the expectations and perspectives on this have changed and are getting more attention in the recent years. Good examples are the initiatives of “deprescribing.org” [8] and the “Australian Deprescribing Network [9] (AdeN).” These collaborations provide researchers and clinicians with support and a wide range of information. Since 2019, there is also a working group in Europe called “The Northern European Researchers in Deprescribing (NERD) Network,” which is also multidisciplinary and engaged to the complex issue of deprescribing. The vision of the initiators is to inform and share insights on new approaches and research findings with healthcare providers, researchers and the general public.

We conducted a scoping review of published drug discontinuation trials to (1) describe the scope (i.e., the diverse methodological characteristics, number and nature) in this research field and (2) discuss the research results.

## Methods

This scoping review followed the framework outlined by Arksey and O’Malley [10] and subsequent recommendations made by Levac et al. [11], in order to map the study designs of trials evaluating the discontinuation of one or more long-term medications. We define long-term medication as drugs, which are not prescribed for a specified time period.

We carried out the following steps: (1) searching for relevant studies, (2) selecting studies based on pre-defined inclusion criteria, (3) extracting data, and (4) summarizing and reporting the results.

The results of this review are reported in accordance with the PRISMA -ScR checklist (Preferred Reporting Items for Systematic reviews and Meta-Analyses extension for Scoping Reviews [12]; Additional file 1).

### Search strategy

We performed a systematic literature search in Medline (PubMed), The Cochrane Library, EMBASE, CINAHL, Web of Science, and PsycINFO. The initial search was done in January 2016 and updated in March 2021. The databases were searched since its inception and covered all published references up to the end of the year 2020.

We used the following search syntax: The term “discontinuation” in synonymous terms (in title) OR the MESH term “Safety-Based Drug Withdrawals” AND the MESH term “Drug therapy.” References including the terms “alcohol” or “tobacco” in the title were excluded. References with non-human study populations were excluded by a filter. The entire syntax and the search process exemplary for Medline is presented in Additional file 2.

### Inclusion and exclusion criteria

We included studies in which the discontinuation of a drug was evaluated. There was no restriction regarding research question, discipline, drug class, or study outcome. We included all prospective study designs (e.g., RCTs, observational studies). We excluded observational studies of drugs stopped spontaneously outside studies by either patients or clinicians and studies of multiple drug interventions. Furthermore, we excluded references with the following characteristics: retrospective study design; qualitative studies; case reports; no original research article; no abstract available; other publication language than English, German, French, or Spanish.

### Selection of studies

References thus identified underwent a two-step selection process. First, title and abstract were screened regarding to the following predefined criteria: “original research article,” “aim of the study was the discontinuation of at least one medication controlled by investigator,” “long-term medication,” “study of humans,” and “no retrospective study design.” Only references fulfilling all of these criteria were subsequently analyzed as full-text regarding the inclusion/exclusion criteria. Hits were checked by two independent reviewers (NG, KK). Disagreements were discussed with a third person (AV).

### Data extraction

For each included study, we recorded relevant bibliographic information (title, names of the authors, publication year). The investigators’ motivation for conducting a drug discontinuation trial was retrieved in its original wording, usually from the introduction section, e.g., “medication is associated with adverse effects” or “[doubt] whether medication is still effective.” Similarly, we extracted the original goal of treatment (why the medication considered was initially prescribed) in the words of the authors, e.g., “lowering blood pressure” or “suppressing cerebral convulsions.” For both “motivation for discontinuation” and “goal of treatment,” we wrote categorizations allowing us to categorize our findings. The categories for these complex domains were developed and tested iteratively.

For drugs investigated, we recorded active ingredient and the conditions it had been prescribed for (categorized by AV). Furthermore, we documented study design, the method of discontinuation (e.g., tapering vs. immediate stopping), the average follow-up period, the number of study participants, and the primary study outcome(s). If explicit information regarding the study type was lacking, we tried to derive a conclusion based on methodological information provided by authors. As a result, we classified studies as (1) “RCT;” (2) quasi-experimental, i.e., at least two study arms with at least one control arm with continued drug treatment, but no randomization( and (3) uncontrolled cohort study, i.e., a prospective study without control arm. In addition, we extracted the average duration of drug treatment before inclusion in the study.

### Summarizing and reporting the results

We calculated descriptive statistics (frequencies and percentages) for each extracted category. All statistical analyses were performed with SPSS software (v 22.0; IBM Corporation, Armonk, NY, USA).

## Results

### Search result and study selection

**Fig. 1:**
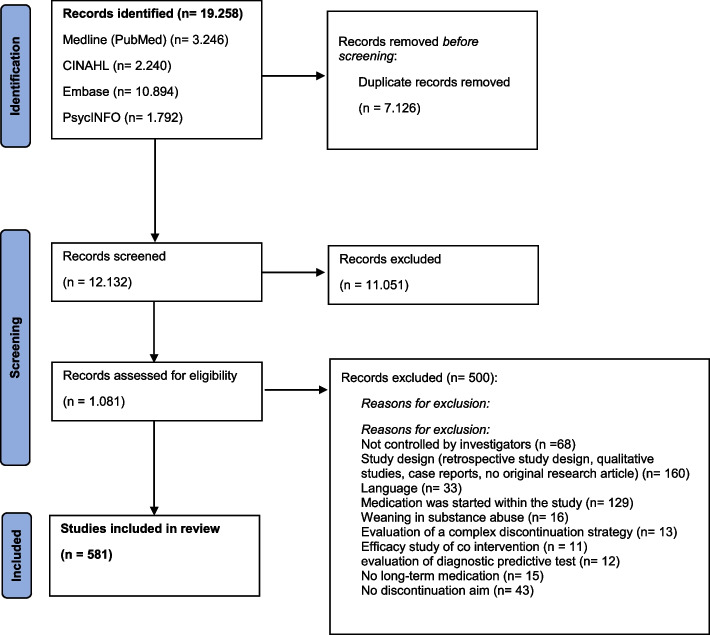
PRISMA flow diagram [12]

Our electronic database search identified 19.258 references. After the exclusion of 7.126 duplicates, − 12.132 references underwent the title and abstract screening. During this process, we excluded 11.051 records, which did not comply with the inclusion criteria. Reading of 1.081 full texts led to the exclusion of 500 articles; we thus included 581 studies as the sample for this report. The numbers of records identified, screened, excluded, and included are shown in the PRISMA—ScR flow chart (Fig. 1).

### Included studies

The majority of the identified drug discontinuation trials originate from Europe (54%; 315/581), North America (27%; 156/581), or Asia (14%; 81/581). Internal medicine (42%; 245/579), psychiatry (24%; 139/579), and neurology (10%; 59/579) were the most common clinical disciplines. From the perspective of diseases originally treated by drugs considered for discontinuation, psychiatric disorders were the most common (22%). The next common diseases groups were state after transplantation/immunosuppressive treatment (12%), epilepsy (12%), and cardiovascular diseases (10%); see Table 1. Most studies were conducted in an outpatient setting (79%), 18% with hospitalized patients, and 2.5% in mixed settings. Therefore, in the presentation of results that now follows, the denominator in each case reports the total number of studies from which the information could be extracted.

**Table 1:** Disease and drug groups of the discontinued medication (*n* = 558)

	*n*	Col %
Psychiatric disorder	122	21.9
Condition after transplantation	67	12.0
Epilepsy	66	11.8
Cardiovascular disease	58	10.4
Cancer disease	35	6.3
HIV or hepatitis	32	5.7
Endocrine diseases diabetes, rheumatism, lung disease, neurological disease, dementia, gastrointestinal diseases, diabetes, fungal disease, pain disorder		Each 1–5%
Mental retardation, post menopause, autoimmune disease, kidney disease, osteoporosis, eye disease, prostate disease, coagulation disease, collagenosis, dermatological disease, chromosome aberration		Each < 1%
Discontinued drug groups (*n* = 560)		
Antipsychotics	165	29.5
Immunosuppressants	82	14.6
Hormone therapy	57	10.2
Antiepileptic drugs	49	8.8
Corticoid therapy	47	8.4
Antiviral drugs, antihypertensive drugs, diuretics, antibiotics, Parkinson’s disease medications, antifungal medication, cytostatic drugs, antidiabetic drugs, anticoagulants, bisphosphonate therapy, cardiovascular medications		Each 1–5%
Opioids, antacid therapy, bronchospasmolytics, antidementia drugs, statins, antiprotozoics, analgesics, antihistamines, biliary therapeutics		Each < 1%

All included studies are shown in Additional file 3.

### Study characteristics

#### Study design

In nearly half of the reports (45%; 264/581), the authors did not explicitly state the study design. Study designs included RCTs, non-randomized studies, quasi-experimental studies, cohort studies, pre-post designs, and case series. Among the publications with explicitly mentioned study designs, RCTs were the most frequent ones (57%; 172/303), in 19% (58/303) a cohort study without controls, and in 15% (45/303) a non-randomized trial design with at least one parallel control arm. Including studies with implicit or indirect descriptions of study design allowed us to categorize overall 96% (556/581) of publications. Among these, only about a third could be characterized as RCTs (34%; 189/556). Almost one half of studies were uncontrolled cohort studies consisting of one single arm (46%; 256/556). The remaining studies (20%, 111/556) had at least two arms including one control arm, but no randomization (Table 2).

**Table 2:** Study design from the review authors (*n* = 556)

	*n*	Col %
Uncontrolled cohort studies (single arm)	256	46
RCT	189	34.1
At least two arms including one control arm, but no randomization	111	19.9

#### Primary outcomes

Primary outcomes could be characterized as follows: “biochemical measurements,” e.g., antibody titer (65%), “symptoms” (e.g., like pain intensity) in 62%, “imaging” (e.g., MRT findings) in 18% of all studies, “quality of life,” “morbidity” and “mortality” occurred together much less frequently in 15% of studies (Table 3).

**Table 3:** Primary outcomes (*n* = 575)

	*n*	Col %
Biochemical measurements	373	64.9
Symptoms	360	62.6
Medical imaging	102	17.7
Quality of life	44	7.7
Morbidity	33	5.7
Mortality	10	1.7

Outcome data extraction was done out of 575 studies; some studies used several outcomes simultaneously

In about two thirds of studies (380/581), the authors reported discontinuation rates as a secondary outcome. The rates of successful discontinuation showed a broad range; see Table 4.

**Table 4:** Discontinuation rates (*n* = 380)

	*n*	Col %
80 to 100%	82	21.6
60 to < 80%	64	16.8
40 to < 60%	94	24.7
20 to < 40%	78	20.5
< 20%	62	16.3

#### Sample sizes and follow-up

Three fourths of the studies (77%; 441/576) had less than 100 participants. The remaining studies had between 100 and 500 participants (100–200 participants: 14%; 80/576/201–500 participants: 6%; 36/576); 4% had more than 500 participants (21/576). The majority of the studies had a follow-up period of under 1 year (66%), one quarter (25%) between 1 and 3 years; 9% of the studies had a follow-up time over 3 years.

#### Discontinuation procedures

We identified two discontinuation procedures: in 41% (226/557) of the studies, the medication was tapered down before it was finally discontinued. An abrupt withdrawal took place in 28% of trials. In 27%, we found a combination of the two procedures. A replacement medication instead of the discontinuation medication was given in 8% of the discontinuation trials.

#### Objectives of original treatment

After studying the treatment objectives of included trials, we identified four categories: “improvement of prognosis,” “symptom control,” “prophylaxis,” and “cure.” The majority of the discontinued medication was initially prescribed to improve the prognosis of a chronic disease [e.g., antihypertensive agents against hypertension] (60%; 351/581). The second most common aim was to control symptoms [e.g., analgesics against pain; antiemetic against nausea] (31%; 180/581). The category “prophylaxis” comprised the prevention of disease in high-risk groups, such as antibiotic drug taken by HIV-infected individuals to prevent *P. carinii* chest infection. This kind of studies were uncommon (5%; 31/581). DDTs evaluating medication with curative intent [e.g., antibiotics against infections] were also rare (3%; 19/581).

#### Justification for drug discontinuation trials

Justifications or motivations for conducting a drug discontinuation trial from the investigators point of view could be grouped in seven categories (Table 5): the most frequently given reason for conducting a DDT were concerns for drug toxicity [e.g., rheumatoid arthritis treatment with TNF inhibitors (49%) [13]. In one fifth (22%), the study medication was discontinued to investigate the efficacy of the drug [e.g., to determine whether digoxin is effective in patients with chronic heart failure [14]. The third frequent reason were doubts regarding the benefit of the medication in the individual or a specified group [e.g., in children with epilepsy after 1 year without seizures, the necessity for continuation of antiepileptic drugs is unclear [15] (14%). Other reasons change of evidence and/or guideline recommendations (7%) or when patients use a medication without a sufficient evidence base and thus unknown benefit and/or safety (14%). In rare cases, drug discontinuation trials were conducted because of high costs of treatment (2%) or because of a high risk of drug interactions (0.3%).

**Table 5:** Motivation for conducting drug discontinuation trial: investigators’ perspective (*n* = 574)

	*n*	Col %
High risk for drug toxicity	280	48.8
Investigating efficacy by discontinuation	124	21.6
Doubts regarding benefit in particular group or case	79	13.8
Changed management of condition (e.g., medication will be exchanged through another therapy)	42	7.3
Insufficient evidence for medication	33	5.7
High costs	14	2.4
High interaction risk	2	0.3

We tabulated primary outcomes and follow-up periods according to the treatment objectives. Trials evaluating medication for improvement of prognosis mostly used biochemical measurements (44%), symptoms (31%), and medical imaging (12%) as primary outcome. Most of these studies had a follow-up time under 1 year (67%). Studies discontinuing medication for symptom control mostly used symptoms as primary outcome parameters (45%). The majority of these studies had a follow-up time under 1 year (64%) (see Tables 6 and 7).

**Table 6:** Outcomes investigated among the different categories of treatment goals

Primary outcomes	Initial treatment goal of the discontinued medication							
	**Improvement of prognosis**		**Symptom control**		**Prophylaxis**		**Curative intention**	
	*n*	Col %	*n*	Col %	*n*	Col %	*n*	Col %
**Biochemical measurements**	239	43.6	89	30.9	27	50.9	16	57.1
**Symptoms**	211	38.5	130	45.1	10	18.9	6	21.4
**Medical imaging**	67	12.2	30	10.4	2	3.8	3	10.7
**Quality of life**	17	3.1	24	8.3	1	1.9	2	7.1
**Morbidity**	8	1.5	13	4.5	11	20.8	1	3.6
**Mortality**	6	1.1	2	0.7	2	3.8	0	-
**At all**	548	100.0	288	100	53	100	28	100

**Table 7:** Follow-up periods among the different categories of treatment aims

Follow-up periods	Initial treatment aim of the discontinued medication							
	**Improvement of prognosis**		**Symptom control**		**Prophylaxis**		**Curative intention**	
	*n*	Col %	*n*	Col %	*n*	Col %	*n*	Col %
** ≤ 1 month**	11	4.3	3	2.1	5	19.2	2	15.4
** ≤ 3 months**	39	15.2	14	9.9	1	3.8	0	-
** ≤ 6 months**	59	42.6	40	28.2	6	23.1	4	30.8
**7 to < 12 month**	63	24.6	34	23.9	3	11.5	4	30.8
** > 1 to 3 years**	60	23.4	39	27.5	8	30.8	2	15.4
** > 3 to 5 years**	17	6.6	9	6.3	2	7.7	0	-
** > 5 years**	7	2.7	3	2.1	1	3.8	0	-
**At all**	256	100.0	142	100.0	26	100.0	13	100.0

## Discussion

### Important findings

We chose a broad focus regarding topics and study design to provide an over view over DDTs published so far.

Among motivations for considering drug discontinuation and conducting DDTs, doubts on effectiveness and/or safety of long-term treatments predominate. Study designs include among others uncontrolled cohorts, quasi-experimental studies, and RCTs (34%).

### Implication and relevance of findings

Discontinuation trials of drugs improving prognosis are twice as common as those addressing symptomatic treatment. This is plausible because for this kind of drugs evidence from discontinuation studies are of particular value for clinicians. However, evaluating the effect of withdrawing disease-modifying drugs is demanding in terms of length of follow-up, sample size, and risk study participants are exposed to.

In this setting, investigators often resort to surrogate outcomes such as biochemical measurements or medical imaging to detect possible harm early enough to prevent manifest clinical deterioration, e.g., graft rejection rates (stopping steroids after kidney transplant), BPH symptoms (stopping alpha-blocker or 5-reductase inhibitor), and blood pressure (stopping BP-lowering drugs).

This has resulted in primary outcomes and the follow-up periods of studies with disease-modifying drugs being no different from others (see Tables 6 and 7). Although studies of this kind require less resources, surrogates do not capture the experience of chronic conditions sufficiently [16]. Surrogate outcomes thus have only limited value in the primary evaluation of a drug; the same considerations should apply in the setting of drug discontinuation evaluation.

Studies of drugs prescribed in curative or prophylactically intention were comparatively uncommon: prophylaxis (medication for preventing illness in high-risk groups) and curative intention (medicine to heal a disease, mostly in an acute proceeding). The first is generally an infrequent indication for prescribing; the latter rarely leads to long-term treatment and is thus less relevant as a research topic.

The study type chosen by investigators also leaves room for improvement. Only one third of studies in this review were RCTs. Single-cohort and non-randomized parallel control studies were common. To inform clinical decision-making, randomization between discontinuation, i.e., the investigational treatment, and continuation of treatment should become the preferred study type to reduce bias.

Interestingly, most discontinuation efforts have targeted specific subgroups, such as older people. This is a group often not included in primary evidence-generating RCTs [3]. Clinicians are used to extrapolating from these studies to their own patients. However, doubts regarding the effectiveness and safety of a drug in older persons and other vulnerable groups typically motivate discontinuation studies. This example shows how primary efficacy and deprescribing studies are related to each other.

### Previously published research of discontinuation trials

There are already reviews that deal with the issue of discontinuation. However, most of them focus on specific substance classes. The included studies have very small numbers of cases and are overall very heterogeneous [17]. Fortunately, the number of studies supporting the requirement for more detailed research on evidence-based drug discontinuation processes is steadily increasing [18, 19].

Several authors who have investigated DDTs, described similar methodically deficiency [17, 18, 20]

Thompson et al. conducted an expert discussion about future directions for deprescribing research in March 2018 [21]. They formulated six priority areas: “(1) conducting high-quality and long-term clinical trials that measure patient-important outcomes, (2) focusing on patient involvement and perspectives, (3) investigating the pharmacoeconomics of deprescribing interventions, (4) understanding deprescribing interventions in different populations, (5) generating evidence on clinical management during deprescribing (e.g., managing adverse drug withdrawal effects, subsequent re-prescribing), and (6) implementing interventions in clinical practice.” [21].

Another approach to improve the quality of DDTs was elaborated by Viniol et al. They developed a new typology of research aims and corresponding methodological recommendations for trials evaluating drug discontinuation. They identified three situations in which there is often uncertainty when discontinuing medication. They defined three types of studies: (1) uncertainty regarding the effectiveness and/or safety of a drug, (2) uncertainty regarding the procedure of discontinuing a previously taken drug, and (3) uncertainty regarding the effectiveness of complex strategies used to discontinue one or more drugs [22]. In order to optimally design and adequately report DDTs planned for the future, the CONSORT Statement was elaborated by Blom et al. [23].

### Strengths and limitations

The strengths of this scoping review is the comprehensive literature search. No restrictions were made on the year of publication for the study selection.

Furthermore, we did not specify any predefined diseases or drug groups as inclusion criteria. Our comprehensive dataset thus provides critical overview of a wide range of methodological approaches used to conduct and report DDTs.

We limited our search to databases covering multiple disciplines such as Medline (PubMed), CINAHL, PsycINFO, Embase, and Web of Science Core Collection. At present, discontinuation studies are not explicitly labeled as such and must be identified indirectly (see our search strategy). Although we likely missed single studies fulfilling our inclusion criteria, our database can be regarded as sufficient for a review focusing on the methodological state of a particular field. The evaluation of the deprescribing of drugs is in flux with rapid changes of research questions and designs.

The studies identified by our search deserve more detailed analysis and discussion of their design components, such as choice of (multiple) outcomes, blinding, or statistical evaluation. These aspects will be subject to an additional publication.

### Implications for research and practice

The increasing burden of disease due to multimedication brings the relevance of drug discontinuation and related research to the forefront. This scoping review shows that discontinuation studies are increasingly being conducted. However, compared to the multitude of clinical discontinuation questions, there are currently relatively few discontinuation studies. A growing awareness of the relevance of weaning research will not be sufficient for more studies to be conducted. The studies are methodologically challenging and expensive. Since the pharmaceutical industry by its nature has no interest in funding drug discontinuation studies, there is a need for public funding sources through which scientists can obtain research funding for drug discontinuation studies.

The results of the scoping review suggest that the methodological quality of many drug discontinuation studies is inadequate. The already initiated development of methodological recommendations/standards is an important step in this direction. Challenging at this point is the great diversity of the initial clinical questions. Depending on the initial situation (doubts about efficacy and/or safety, efficacy of discontinuation procedures or complex discontinuation strategies, etc.), different methodological approaches appear appropriate. This must be taken into account in the development of drug discontinuation studies.

## Conclusion

This scoping review illustrates the need for an evidence-based standard for conducting drug discontinuation trials. This standard should include a definition for drug discontinuation trials, a differentiation of the varying research aims with specific methodically recommendations. Against the background of widespread polypharmacy, especially with the increasing portion of prognosis improving drugs, the requirement for drug discontinuation will grow and so will the need for a robust evidence-base. We hope that our scoping review will make a relevant contribution to this goal.

## Supplementary Information

**Additional file 1.** Preferred Reporting Items for Systematic reviews and Meta-Analyses extension for Scoping Reviews (PRISMA-ScR) Checklist.

**Additional file 2. **Entire syntax and the search process exemplary for Medline.

**Additional file 3.** List of all included references.

## Data Availability

All data generated or analyzed during this study are included in this published article.

## Abbreviations

AdeN: Australian Deprescribing Network
BMBF: Federal Ministry of Education and Research
CINAHL: Cumulative Index to Nursing and Allied Health Literature
DDT: Drug Discontinuation Trial
EMBASE: Excerpta Medica Database
HIV: Human immunodeficiency virus
MeSH: Medical Subject Heading
NERD: The Northern European Researchers in Deprescribing
*P.**carinii*: *Pneumocystis* *carinii*
PRISMA-ScR: Preferred Reporting Items for Systematic reviews and Meta-Analyses extension for Scoping Reviews
RCT: Randomized controlled trial
SPSS: Statistical Package for the Social Sciences
TNF: Tumor necrosis factor
