# Supplementary material for: Mapping the methodological diversity of published drug discontinuation studies—a scoping review of study topics, objectives, and designs
Source: Trials. 2023 Jan 26;24:58. doi: 10.1186/s13063-023-07105-6 (PMC9878942; doi:10.1186/s13063-023-07105-6)
Supplement: Supplementary file 2 — Additional file 2. Entire syntax and the search process exemplary for Medline. [file 13063_2023_7105_MOESM2_ESM.docx]

Medline (PubMed)

23.03.2021

| **ID** | **Search** | **hits** |
| --- | --- | --- |
| #1 | "Safety-Based Drug Withdrawals"[Mesh] | 399 |
| #2 | stop* [ti] | 15.896 |
| #3 | discontinu* [ti] | 10.819 |
| #4 | withdraw* [ti] | 23.918 |
| #5 | cessation [ti] | 15.862 |
| #6 | deprescri* [ti] | 550 |
| #7 | #1 OR #2 OR #3 OR #4 OR #5 OR #6 | 66.935 |
| #8 | “Drug Therapy"[Mesh] | 1.390.076 |
| #9 | #7 AND #8 | 7.108 |
| #10 | alcohol* [ti] | 144.398 |
| #11 | tobacco [ti] | 42.089 |
| #12 | smok* [ti] | 93.872 |
| #13 | cigarett* [ti] | 25.873 |
| #14 | # 10 OR #11 OR #12 OR #13 | 271.459 |
| #15 | #9 NOT #14 | 6.097 |
| #16 | ((Address[ptyp] OR Autobiography[ptyp] OR Bibliography[ptyp] OR Biography[ptyp] OR pubmed books[filter] OR Case Reports[ptyp] OR Classical Article[ptyp] OR Clinical Conference[ptyp] OR Comment[sb] OR Congress[ptyp] OR Consensus Development Conference[ptyp] OR Consensus Development Conference, NIH[ptyp] OR Corrected and Republished Article[sb] OR Dataset[ptyp] OR Dictionary[ptyp] OR Directory[ptyp] OR Duplicate Publication[ptyp] OR Editorial[ptyp] OR Electronic Supplementary Materials[ptyp] OR Festschrift[ptyp] OR Guideline[ptyp] OR Historical Article[ptyp] OR Interactive Tutorial[ptyp] OR Interview[ptyp] OR Introductory Journal Article[ptyp] OR Lecture[ptyp] OR Legal Case[ptyp] OR Legislation[ptyp] OR Letter[ptyp] OR News[ptyp] OR Newspaper Article[ptyp] OR Overall[ptyp] OR Patient Education Handout[ptyp] OR Periodical Index[ptyp] OR Personal Narrative[ptyp] OR Portrait[ptyp] OR Practice Guideline[ptyp] OR Published Erratum[sb] OR Retracted Publication[sb] OR Retraction of Publication[sb] OR Review[ptyp] OR Scientific Integrity Review[ptyp] OR Twin Study[ptyp] OR Video-Audio Media[ptyp] OR Webcast[ptyp])) | 7.368.707 |
| #17 | #15 NOT #16 | 4.428 |
| #18 | Filters: Publication date 2020/12/31, Humans | **3.246** |
