# Supplementary material for: Mapping the methodological diversity of published drug discontinuation studies—a scoping review of study topics, objectives, and designs
Source: Trials. 2023 Jan 26;24:58. doi: 10.1186/s13063-023-07105-6 (PMC9878942; doi:10.1186/s13063-023-07105-6)
Supplement: Supplementary file 3 — Additional file 3. List of all included references. [file 13063_2023_7105_MOESM3_ESM.docx]

| **Appendix 3: List of all included references** | | | |
| --- | --- | --- | --- |
| **Authors** | **Publi- cation  year** | **Titel** | **Journal** |
| Prien, Robert F.; Cole, Jonathan O.; Belkin, Naomi F. | 1969 | Relapse in chronic schizophrenics following abrupt withdrawal of tranquillizing medication | The British Journal of Psychiatry |
| Franzen, G. | 1971 | Serum cortisol in chronic schizophrenia: Changes in the diurnal rhythm and psychiatric mental status on withdrawal of drugs | Psychiatria clinica |
| Hughes, R. C.; Polgar, J. G.; Weightman, D.; Walton, John N. | 1971 | Levodopa in parkinsonism: The effects of withdrawal of anticholinergic drugs | BMJ: British Medical Journal |
| Polizos, Polizoes; Engelhardt, David M.; Hoffman, Stanley P.; Waizer, Jonas | 1973 | Neurological consequences of psychotropic drug withdrawal in schizophrenic children | Journal of Autism & Childhood Schizophrenia |
| Andrews, P.; Hall, J. N.; Snaith, R. P. | 1976 | A controlled trial of phenothiazine withdrawal in chronic schizophrenic patients | The British Journal of Psychiatry |
| Hogarty, Gerard E.; Ulrich, Richard F.; Mussare, Frank; Aristigueta, Narciso | 1976 | Drug discontinuation among long term, successfully maintained schizophrenia outpatients | Diseases of the Nervous System |
| Kristensen B.O.; Steiness E.; Weeke J. | 1978 | Propranolol withdrawal and thyroid hormones in patients with essential hypertension | Clin. Pharmacol. Ther. |
| Winsberg, Bertrand G.; Hurwic, Maria J.; Sverd, Jeffrey; Klutch, Albert | 1978 | Neurochemistry of withdrawal emergent symptoms in children | Psychopharmacology |
| Shee, C. D.; Medd, W. E. | 1979 | Stopping phenformin treatment in a diabetic clinic | Current medical research and opinion |
| Woggon, Brigitte; Bickel, P.; Schnyder, B. | 1979 | Discontinuation of antipsychotic drugs in chronic schizophrenic patients: II. Psychological test and psychopathological differences between patients with and without relapse | International Pharmacopsychiatry |
| Capstick, Norman | 1980 | Long-term fluphenazine decanoate maintenance dosage requirements of chronic schizophrenic patients | Acta psychiatrica Scandinavica |
| Dencker, S. J.; Lepp, M.; Malm, U. | 1980 | Do schizophrenics well adapted in the community need neuroleptics? A depot neuroleptic withdrawal study | Acta psychiatrica Scandinavica. Supplementum |
| Korman M.G.; Hetzel D.J.; Hansky J. | 1980 | Relapse of duodenal ulcer after cessation of long-term cimetidine treatment. A double-blind controlled study | Dig. Dis. Sci. |
| Siitonen, O.; Aro, A.; Huttunen, J. K.; Juvonen, H.; Jarvinen, R.; Korhonen, T.; Palomaki, P.; Ritala, P. | 1980 | Effect of discontinuation of biguanide therapy on metabolic control in maturity-onset diabetics | Lancet (London, England) |
| Bolli, P.; Buhler, F. R.; Raeder, E. A.; Amann, F. W.; Meier, M.; Rogg, H.; Burckhardt, D. | 1981 | Lack of beta-adrenoreceptor hypersensitivity after abrupt withdrawal of long-term therapy with oxprenolol | Circulation |
| Branchey, Marc H.; Branchey, Laure B.; Richardson, Mary A. | 1981 | Effects of neuroleptic adjustment on clinical condition and tardive dyskinesia in schizophrenic patients | The American journal of psychiatry |
| Jellinek, T.; Gardos, G.; Cole, J. O. | 1981 | Adverse effects of antiparkinson drug withdrawal | The American journal of psychiatry |
| Ohsawa N.; Kobayashi I.; Suwa K. | 1981 | TSH and prolactin secretions in Hashimoto's thyroiditis following withdrawal of thyroid hormone therapy | ENDOCRINOL. JPN. |
| Oller-Daurella, Luis; Sanchez, Miguel E.; Oller F.-V., Luis | 1981 | Possibilidades de supresion del tratamiento en epilepticos libres de crisis durante mas de cinco anos. = Results on discontinuance of antiepileptic drugs in patients seizure-free for more than five years | Archivos de NeurobiologÃ­a |
| Schneider A.B.; Line B.R.; Goldman J.M.; Robbins J. | 1981 | Sequential serum thyroglobulin determinations, 131I scans, and 131I uptakes after triiodothyronine withdrawal in patients with thyroid cancer | J. Clin. Endocrinol. Metab. |
| Wistedt, B. | 1981 | A depot neuroleptic withdrawal study. A controlled study of the clinical effects of the withdrawal of depot fluphenazine decanoate and depot flupenthixol decanoate in chronic schizophrenic patients | Acta psychiatrica Scandinavica |
| Zander, K. J.; Fischer, B.; Zimmer, R.; Ackenheil, M. | 1981 | Long-term neuroleptic treatment of chronic schizophrenic patients: Clinical and biochemical effects of withdrawal | Psychopharmacology |
| Bialos, Donald; Giller, Earl; Jatlow, Peter; Docherty, John; Harkness, Laurie | 1982 | Recurrence of depression after discontinuation of long-term amitriptyline treatment | The American journal of psychiatry |
| De Vaan G.A.M.; Van Munster P.J.J.; Bakkeren J.A.J.M. | 1982 | Recovery of immune function after cessation of maintenance therapy in acute lymphoblastic leukemia (ALL) of childhood | EUR. J. PEDIATR. |
| Delva, N. J.; Letemendia, F. J.; Prowse, A. W. | 1982 | Lithium withdrawal trial in chronic schizophrenia | The British Journal of Psychiatry |
| Goldman J.M.; Weintraub B.D. | 1982 | Recovery of pituitary secretion of thyrotropin and its free α- and β-subunits after triiodothyronine withdrawal | J. Clin. Endocrinol. Metab. |
| Hacki M.A.; Angehrn W.; Cavegn H.R.; Brandli O. | 1982 | Discontinuation of long-term digitalis therapy in elderly patients - A controlled trial | ZUR LANGZEIT-DIGITALISTHERAPIE ALTERER PATIENTEN. IST DIE MEHRZAHL UNNOTIG DIGITALISIERT? |
| Hopkins, D. R.; Sethi, K. B.; Mucklow, J. C. | 1982 | Benzodiazepine withdrawal in general practice | The Journal of the Royal College of General Practitioners |
| Laughren, Thomas P.; Battey, Yvonne; Greenblatt, David J.; Harrop, Daniel S. | 1982 | A controlled trial of diazepam withdrawal in chronically anxious patients | Acta psychiatrica Scandinavica |
| Levinson, P. D.; Khatri, I. M.; Freis, E. D. | 1982 | Persistence of normal BP after withdrawal of drug treatment in mild hypertension | Archives of internal medicine |
| Margo, Andrew; McMahon, Paul | 1982 | Lithium withdrawal triggers psychosis | The British Journal of Psychiatry |
| Odejide, O. A.; Aderounmu, A. F. | 1982 | Double-blind placebo substitution: withdrawal of fluphenazine decanoate in schizophrenic patients | The Journal of clinical psychiatry |
| Ohtsuka Y.; Yamatogi Y.; Oka E.; Ohtahara S. | 1982 | Treatment and discontinuation of antiepileptic drugs in childhood epilepsy | FOLIA PSYCHIATR. NEUROL. JPN. |
| Rangno, R. E.; Nattel, S.; Lutterodt, A. | 1982 | Prevention of propranolol withdrawal mechanism by prolonged small dose propranolol schedule | The American journal of cardiology |
| Wistedt, B.; Jorgensen, A.; Wiles, D. | 1982 | A depot neuroleptic withdrawal study. Plasma concentration of fluphenazine and flupenthixol and relapse frequency | Psychopharmacology |
| Baker, Linda A.; Cheng, Louis Y.; Amara, I. B. | 1983 | The withdrawal of benztropine mesylate in chronic schizophrenic patients | The British Journal of Psychiatry |
| Bournerias, F.; Monnier, N.; Reveillaud, R. J. | 1983 | Risk of orally administered aluminium hydroxide and results of withdrawal | Proceedings of the European Dialysis and Transplant Association. European Dialysis and Transplant Association |
| Carter, Graham | 1983 | The abrupt withdrawal of antiparkinsonian drugs in mentally handicapped patients | The British Journal of Psychiatry |
| Daly C.; Edwards H. | 1983 | Withdrawal of digoxin in general practice in elderly patients | IR. MED. J. |
| Gheorghiade M.; Beller G.A. | 1983 | Effects of discontinuing maintenance digoxin therapy in patients with ischemic heart disease and congestive heart failure in sinus rhythm | Am. J. Cardiol. |
| Lepantalo M.; Von Knorring J.; Lindfors O.; Scheinin T.M. | 1983 | The effect of withdrawal of beta-adrenergic blockade on intermittent claudication | Angiology |
| Maland L.J.; Lutz L.J.; Castle C.H. | 1983 | Effects of withdrawing diuretic therapy on blood pressure in mild hypertension | Hypertension |
| Perenyi A.; Gardos G.; Samu I. | 1983 | Changes in extrapyramidal symptoms following anticholinergic drug withdrawal | CLIN. NEUROPHARMACOL. |
| Petursson H.; Gudjonsson G.H.; Lader M.H. | 1983 | Psychometric performance during withdrawal from long-term benzodiazepine treatment | Psychopharmacology |
| Taggart A.J.; McDevitt D.G. | 1983 | Diuretic withdrawal - a need for caution | Curr. Med. Res. Opin. |
| Wistedt, B.; Wiles, D.; Jorgensen, A. | 1983 | A depot neuroleptic withdrawal study neurological effects | Psychopharmacology |
| Wistedt, Bã¶Rje; Palmstierna, Tom | 1983 | Depressive symptoms in chronic schizophrenic patients after withdrawal of long-acting neuroleptics | Journal of Clinical Psychiatry |
| Greil, W.; Haag, H.; Rossnagl, G.; Ruther, E. | 1984 | Effect of anticholinergics on tardive dyskinesia. A controlled discontinuation study | The British journal of psychiatry : the journal of mental science |
| Gualtieri, C. Thomas; Quade, Dana; Hicks, Robert E.; Mayo, James P.; Schroeder, Stephen R. | 1984 | Tardive dyskinesia and other clinical consequences of neuroleptic treatment in children and adolescents | The American journal of psychiatry |
| Ionescu-Tirgoviste C.; Mincu I.; Simionescu L. | 1984 | Disappearance rate of insulin antibodies after discontinuing insulin treatment in 42 Type 2 (non-insulin-dependent) diabetic patients | Diabetologia |
| Jennings G.; Korner P.; Esler M.; Restall R. | 1984 | Redevelopment of essential hypertension after cessation of long term therapy; preliminary findings | CLIN. EXP. HYPERTENS. PART A THEORY PRACT. |
| Jennings, G. L.; Korner, P. I.; Laufer, E.; Esler, M. D.; Burton, D.; Bruce, A. | 1984 | How hypertension redevelops after cessation of long-term therapy | Journal of hypertension. Supplement : official journal of the International Society of Hypertension |
| Johnston D.G.; Hall K.; Kendall-Taylor P. | 1984 | Effect of dopamine agonist withdrawal after long-term therapy in prolactinomas. Studies with high-definition computerised tomography | LANCET |
| Lader, Malcolm | 1984 | Benzodiazepine dependence | Progress in neuro-psychopharmacology & biological psychiatry |
| Muller-Spahn F.; Ackenheil M.; Albus M. | 1984 | Neuroendocrine effects of apomorphine in chronic schizophrenic patients under long-term neuroleptic therapy and after drug withdrawal: Relations to psychopathology and tardive dyskinesia | Psychopharmacology |
| Angrist, B.; Peselow, E.; Rubinstein, M.; Wolkin, A.; Rotrosen, J. | 1985 | Amphetamine response and relapse risk after depot neuroleptic discontinuation | Psychopharmacology |
| Bagdy G.; Perenyi A.; Frecska E. | 1985 | Decrease in dopamine, its metabolites and noradrenaline in cerebrospinal fluid of schizophrenic patients after withdrawal of long-term neuroleptic treatment | Psychopharmacology |
| Bendz H. | 1985 | Kidney function in a selected lithium population. A prospective, controlled, lithium-withdrawal study | Acta Psychiatr. Scand. |
| Frecska, Ede; Perã©Nyi, Andrã¡S; Bagdy, Gyã¶Rgy; Rã©Vai, Katalin | 1985 | CSF dopamine turnover and positive schizophrenic symptoms after withdrawal of long-term neuroleptic treatment | Psychiatry research |
| Giller, E.; Bialos, D.; Harkness, L.; Jatlow, P.; Waldo, M. | 1985 | Long-term amitriptyline in chronic depression | Hillside Journal of Clinical Psychiatry |
| Hohfeld R.; Toyka K.V.; Besinger U.A. | 1985 | Myasthenia gravis: Reactivation of clinical disease and of autoimmune factors after discontinuation of long-term azathioprine | Ann. Neurol. |
| Langford H.G.; Blaufox M.D.; Oberman A. | 1985 | Dietary therapy slows the return of hypertension after stopping prolonged medication | J. Am. Med. Assoc. |
| Ludgate, J.; O'Dwyer, R.; Callaghan, N. | 1985 | An improvement in cognitive function following polypharmacy reduction in a group of epileptic patients | Acta neurologica Scandinavica |
| McInnis, M.; Petursson, H. | 1985 | Withdrawal of trihexyphenidyl | Acta psychiatrica Scandinavica |
| Shinnar S.; Vining E.P.G.; Mellits E.D. | 1985 | Discontinuing antiepileptic medication in children with epilepsy after two years without seizures: A prospective study | New Engl. J. Med. |
| Stellon, A. J.; Hegarty, J. E.; Portmann, B.; Williams, R. | 1985 | Randomised controlled trial of azathioprine withdrawal in autoimmune chronic active hepatitis | Lancet (London, England) |
| Wilkins C.E.; Khurana M.S. | 1985 | Digitalis withdrawal in elderly nursing home patients | J. Am. Geriatr. Soc. |
| Busto, Usoa; Sellers, Edward M.; Naranjo, Claudio A.; Cappell, Howard; Sanchez-Craig, Martha; Sykora, Kathy | 1986 | Withdrawal reaction after long-term therapeutic use of benzodiazepines | The New England journal of medicine |
| Cook, Brian L.; Helms, Paul M.; Smith, Robert E.; Tsai, Merling | 1986 | Unipolar depression in the elderly: Reoccurrence on discontinuation of tricyclic antidepressants | Journal of affective disorders |
| Dencker, Sven J.; Malm, Ulf; Lepp, M. | 1986 | Schizophrenic relapse after drug withdrawal is predictable | Acta psychiatrica Scandinavica |
| Gallassi R.; Lorusso S.; Stracciari A. | 1986 | Withdrawal of phenobarbital and carbamazepine in epileptic patients: A preliminary neuropsychological report | Acta Neurol. Scand. |
| Kugoh, T.; Hosokawa, K. | 1986 | A trial of discontinuation of barbiturates in patients with secondary generalized epilepsy | The Japanese journal of psychiatry and neurology |
| Mellman, Thomas A.; Uhde, Thomas W. | 1986 | Withdrawal syndrome with gradual tapering of alprazolam | The American journal of psychiatry |
| Schweizer, Edward; Rickels, Karl | 1986 | Failure of buspirone to manage benzodiazepine withdrawal | The American journal of psychiatry |
| Van Der Leeden H.; Dijkmans B.A.C.; Hermans J.; Cats A. | 1986 | A double-blind study on the effect of discontinuation of gold therapy in patients with rheumatoid arthritis | Clin. Rheumatol. |
| Bouma P.A.D.; Peters A.C.B.; Arts R.J.H.M.; Stijnen Th.; Van Rossum J. | 1987 | Discontinuation of antiepileptic therapy: A prospective study in children | J. Neurol. Neurosurg. Psychiatry |
| Lader M.; Olajide D. | 1987 | A comparison of buspirone and placebo in relieving benzodiazepine withdrawal symptoms | J. Clin. Psychopharmacol. |
| Metz, S.; Klein, C.; Morton, N. | 1987 | Rebound hypertension after discontinuation of transdermal clonidine therapy | The American journal of medicine |
| Theodore W.H.; Porter R.J.; Raubertas R.F. | 1987 | Seizures during barbiturate withdrawal: Relation to blood level | Ann. Neurol. |
| Coulter, D. L. | 1988 | Withdrawal of barbiturate anticonvulsant drugs: prospective controlled study | American journal of mental retardation : AJMR |
| Duncan, J. S.; Shorvon, S. D.; Trimble, M. R. | 1988 | Withdrawal symptoms from phenytoin, carbamazepine and sodium valproate | Journal of neurology, neurosurgery, and psychiatry |
| Gallassi, Roberto; Morreale, Angela; Lorusso, Sebastiano; Procaccianti, Gaetano; Lugaresi, Elio; Baruzzi, Agostino | 1988 | Carbamazepine and phenytoin: Comparison of cognitive effects in epileptic patients during monotherapy and withdrawal | Archives of Neurology |
| Kirch D.G.; Jaskiw G.; Linnoila M.; Weinberger D.R.; Wyatt R.J. | 1988 | Plasma amine metabolites before and after withdrawal from neuroleptic treatment in chronic schizophrenic inpatients | Psychiatry Res |
| Pato, Michele T.; Zohar-Kadouch, Rachel; Zohar, Joseph; Murphy, Dennis L. | 1988 | Return of symptoms after discontinuation of clomipramine in patients with obsessive-compulsive disorder | The American journal of psychiatry |
| Sakol, Martyn S.; Power, Kevin G. | 1988 | The effects of long-term benzodiazepine treatment and graded withdrawal on psychometric performance | Psychopharmacology |
| Schlecht L.P.; Brubaker R.F. | 1988 | The effects of withdrawal of timolol in chronically treated glaucoma patients | Ophthalmology |
| Stellon A.J.; Keating J.J.; Johnson P.J.; McFarlane I.G.; Williams R. | 1988 | Maintenance of remission in autoimmune chronic active hepatitis with azathioprine after corticosteroid withdrawal | HEPATOLOGY |
| Alvarez, Norberto | 1989 | Discontinuance of antiepileptic medications in patients with developmental disability and diagnosis of epilepsy | American Journal on Mental Retardation |
| Bromfield, E. B.; Dambrosia, J.; Devinsky, O.; Nice, F. J.; Theodore, W. H. | 1989 | Phenytoin withdrawal and seizure frequency | Neurology |
| Clemens, B. | 1989 | Timing discontinuation of antiepileptic treatment in childhood epilepsies--the role of the sleep deprivation EEG: a preliminary study | The Japanese journal of psychiatry and neurology |
| Findlay, D. J.; Sharma, J.; McEwen, J.; Ballinger, Brian R.; MacLennan, W. J.; McHarg, A. M. | 1989 | Double-blind controlled withdrawal of thioridazine treatment in elderly female inpatients with senile dementia | International journal of geriatric psychiatry |
| Jann, M. W.; Fidone, G. S.; Hernandez, J. M.; Amrung, S.; Davis, C. M. | 1989 | Clinical implications of increased antipsychotic plasma concentrations upon anticonvulsant cessation | Psychiatry research |
| Lal, S. | 1989 | Improvement in spasmodic torticollis following treatment and withdrawal from high dose lorazepam--clinical observations | Progress in neuro-psychopharmacology & biological psychiatry |
| Mitchell A.; Haynes R.B.; Adsett C.A.; Bellissimo A.; Wilczynski N. | 1989 | The likelihood of remaining normotensive following antihypertensive drug withdrawal | J. Gen. Intern. Med. |
| Muijen, Matthijs; Jones, Deric P.; Roy, David; Silverstone, Trevor; Mehmet, All | 1989 | Mianserin withdrawal and the pupil response of depressed and recovered patients: A preliminary report | Biological psychiatry |
| Nobels F.; Van Gaal L.; Rillaerts E.; De Leeuw I. | 1989 | Effect of oral antidiabetic drug withdrawal in Type 2 diabetes | Diabetic Med |
| Ries, Richard K.; Roy-Byrne, Peter P.; Ward, Nicholas G.; Neppe, Vernon; Cullison, Sam | 1989 | Carbamazepine treatment for benzodiazepine withdrawal | The American journal of psychiatry |
| Rubinoff H.; Fireman B.H. | 1989 | Testing for recovery of thyroid function after withdrawal of long-term suppression therapy | J. CLIN. EPIDEMIOL. |
| Schweizer, Edward; Case, Warren G.; Rickels, Karl | 1989 | Benzodiazepine dependence and withdrawal in elderly patients | The American journal of psychiatry |
| Specht U.; Boenigk H.E.; Wolf P. | 1989 | Discontinuation of clonazepam after long-term treatment | Epilepsia |
| Duncan, J. S.; Shorvon, S. D.; Trimble, M. R. | 1990 | Discontinuation of phenytoin, carbamazepine, and valproate in patients with active epilepsy | Epilepsia |
| Joyce, E. M.; Moodley, Parimala; Keshavan, M. S.; Lader, M. H. | 1990 | Failure of clonidine treatment in benzodiazepine withdrawal | Journal of Psychopharmacology |
| Khan, R. S.; Amin, F.; Powchik, P.; Knott, P.; Goldstein, M.; Apter, S.; Kerman, B.; Jaff, S.; Davidson, M. | 1990 | Increments in plasma homovanillic acid concentrations after neuroleptic discontinuation are associated with worsening of schizophrenic symptoms | Progress in neuro-psychopharmacology & biological psychiatry |
| Macarthur, C. | 1990 | Withdrawal of maintenance digoxin from institutionalized elderly | Postgraduate medical journal |
| Monteleone P.; Fiumani P.M.; Franza F.; Maj M. | 1990 | Neuroleptic withdrawal and response to dexamethasone suppression test in chronic schizophrenics | NEUROENDOCRINOL. LETT. |
| Rickels, Karl; Case, Warren G.; Schweizer, Edward; Garcia-Espana, Felipe; Fridman, R. | 1990 | Benzodiazepine dependence: Management of discontinuation | Psychopharmacology bulletin |
| Rickels, Karl; Schweizer, Edward; Case, George; Greenblatt, David J. | 1990 | Long-term therapeutic use of benzodiazepines: I. Effects of abrupt discontinuation | Archives of general psychiatry |
| Risse, Steven C.; Whitters, Alan; Burke, James; Chen, Stephen; Scurfield, R. M.; Raskind, M. A. | 1990 | Severe withdrawal symptoms after discontinuation of alprazolam in eight patients with combat-induced posttraumatic stress disorder | Journal of Clinical Psychiatry |
| Schweizer, Edward; Rickels, Karl; Case, George; Greenblatt, David J. | 1990 | Long-term therapeutic use of benzodiazepines: II. Effects of gradual taper | Archives of general psychiatry |
| Tibblin G.; Aberg H. | 1990 | Non-pharmacological treatment of hypertension: Differences between health centres in patients' blood pressure and success at withdrawal from drugs | Fam. Pract. |
| Udelman, H. D.; Udelman, D. L. | 1990 | Concurrent use of buspirone in anxious patients during withdrawal from alprazolam therapy | The Journal of clinical psychiatry |
| Barton G.; Hicks E.; Patterson V.H.; Swallow M.W.; Bush A.; Finnegean J.A.; Gupta A.; Sharma A.; Thomas K.T.; Ferguson I.T.; Karki C.B.; Walters m. R.; Morrow J.; Richens A.; Wallace S.; Wroe S.J.; Godwin-Austen R.; Davidson A.; Davidson D.L.W. | 1991 | Randomised study of antiepileptic drug withdrawal in patients in remission | LANCET |
| Bykerk V.; Sampalis J.; Esdaile J.M.; Choquette D.; Senecal J.-L.; Danoff D.; Smith C.D.; Cividino A.; Osterland C.K.; Yeadon C. | 1991 | A randomized study of the effect of withdrawing hydroxychloroquine sulfate in systemic lupus erythematosus | New Engl. J. Med. |
| Calev, Avraham; Nigal, Doron; Kugelmass, Sol; Weller, Malcolm P.; Lerer, Bernard | 1991 | Performance of long-stay schizophrenics after drug withdrawal on matched immediate and delayed recall tasks | British Journal of Clinical Psychology |
| Forman D.E.; Coletta D.; Kenny D.; Kosowsky B.D.; Stoukides J.; Rohrer M.; Pastore J.O. | 1991 | Clinical issues related to discontinuing digoxin therapy in elderly nursing home patients | Arch. Intern. Med. |
| Garcia-Borreguero, D.; Bronisch, T.; Apelt, S.; Yassouridis, A.; Emrich, H. M. | 1991 | Treatment of benzodiazepine withdrawal symptoms with carbamazepine | European archives of psychiatry and clinical neuroscience |
| Haugaard, L.; Norregaard, O. F.; Dahl, R. | 1991 | In-hospital sting challenge in insect venom-allergic patients after stopping venom immunotherapy | The Journal of allergy and clinical immunology |
| Joughin N.; Tata P.; Collins M.; Hooper C.; Falkowski J. | 1991 | In-patient withdrawal from long-term benzodiazepine use | BR. J. ADDICT. |
| Klein, Ehud; Mairaz, Rami; Pascal, Martha; Hefez, Albert; Lavie, Peretz | 1991 | Discontinuation of lithium treatment in remitted bipolar patients: Relationship between clinical outcome and changes in sleep-wake cycles | Journal of Nervous and Mental Disease |
| Medical Research Council Antiepileptic Drug Withdrawal Study Group | 1991 | Randomised study of antiepileptic drug withdrawal in patients in remission. Medical Research Council Antiepileptic Drug Withdrawal Study Group | Lancet (London, England) |
| Muiesan M.L.; Agabiti-Rosei E.; Romanelli G.; Beschi M.; Castellano M.; Alari G.; Rizzoni D.; Muiesan G. | 1991 | Improved left ventricular systolic and diastolic function after regression of cardiac hypertrophy, treatment withdrawal, and redevelopment of hypertension | J. CARDIOVASC. PHARMACOL. |
| Ruskin, Paul E.; Nyman, Gary | 1991 | Discontinuation of neuroleptic medication in older, outpatient schizophrenics: A placebo-controlled, double-blind trial | Journal of Nervous and Mental Disease |
| Schweizer, Edward; Rickels, Karl; Case, Warren G.; Greenblatt, David J. | 1991 | Carbamazepine treatment in patients discontinuing long-term benzodiazepine therapy: Effects on withdrawal severity and outcome | Archives of general psychiatry |
| Souza F.G.M.; Mander A.J.; Foggo M.; Dick H.; Shearing C.H.; Goodwin G.M. | 1991 | The effects of lithium discontinuation and the non-effect of oral inositol upon thyroid hormones and cortisol in patients with bipolar affective disorder | J. AFFECT. DISORD. |
| The Canadian Hydroxychloroquine Study Group | 1991 | A randomized study of the effect of withdrawing hydroxychloroquine sulfate in systemic lupus erythematosus. The Canadian Hydroxychloroquine Study Group | The New England journal of medicine |
| Wolf, M. A.; Bailly, L.; Diener, J. M.; Martinet, J. P. | 1991 | Sevrage neuroleptique complet chez des patients schizophrÃ¨nes prÃ©sentant une symptomatologie intense et rÃ©sistant au traitement. = Complete neuroleptic withdrawal in schizophrenic patients with a florid symptomatology resistant to treatment | L'EncÃ©phale: Revue de psychiatrie clinique biologique et thÃ©rapeutique |
| Birzele, Hans-Joachim | 1992 | Benzodiazepine induced amnesia after long-term medication and during withdrawal | European Review of Applied Psychology / Revue EuropÃ©enne de Psychologie AppliquÃ©e |
| Gallassi R.; Morreale A.; Di Sarro R.; Marra M.; Lugaresi E.; Baruzzi A. | 1992 | Cognitive effects of antiepileptic drug discontinuation | Epilepsia |
| Gherpelli, J. L.; Kok, F.; dal Forno, S.; Elkis, L. C.; Lefevre, B. H.; Diament, A. J. | 1992 | Discontinuing medication in epileptic children: a study of risk factors related to recurrence | Epilepsia |
| Hawthorne A.B.; Logan R.F.A.; Hawkey C.J.; Foster P.N.; Axon A.T.R.; Swarbrick E.T.; Scott B.B.; Lennard-Jones J.E. | 1992 | Randomised controlled trial of azathioprine withdrawal in ulcerative colitis | BR. MED. J. |
| Hricik D.E.; Bartucci M.R.; Mayes J.T.; Schulak J.A. | 1992 | The effects of steroid withdrawal on the lipoprotein profiles of cyclosporine-treated kidney and kidney-pancreas transplant recipients | Transplantation |
| Hricik D.E.; Lautman J.; Bartucci M.R.; Moir E.J.; Mayes J.T.; Schulak J.A. | 1992 | Variable effects of steroid withdrawal on blood pressure reduction in cyclosporine-treated renal transplant recipients | Transplantation |
| Jacoby A.; Johnson A.; Chadwick D. | 1992 | Psychosocial outcomes of antiepileptic drug discontinuation | Epilepsia |
| May, T. W.; Bulmahn, A.; Wohlhã¼Ter, M.; Rambeck, B. | 1992 | Effects of withdrawal of phenytoin on cognitive and psychomotor functions in hospitalized epileptic patients on polytherapy | Acta neurologica Scandinavica |
| McLennan, J.; Findlay, D. J.; Sharma, J.; McEwen, J.; Ballinger, B. R.; MacLennan, W. J.; McHarg, A. M. | 1992 | Prolactin response to withdrawal of thioridazine in dementia | International journal of geriatric psychiatry |
| NAUMAN, D.; GREENBERG, B.; MASSIE, B.; BRISTOW, J. D.; CHEITLIN, M. | 1992 | EFFECTS OF STOPPING LONG-TERM VASODILATOR THERAPY IN PATIENTS WITH CHRONIC AORTIC-INSUFFICIENCY | Chest |
| Palm L.; Anderson H.; Elmqvist D.; Blennow G. | 1992 | Daytime sleep tendency before and after discontinuation of antiepileptic drugs in preadolescent children with epilepsy | Epilepsia |
| Rocco, Pier L.; Giavedoni, Anna; Pacella, Giulia | 1992 | Withdrawal from benzodiazepines in a hospital setting: An open trial with buspirone | Current Therapeutic Research |
| Salzman, Carl; Fisher, Janina; Nobel, Kenneth; Glassman, Randy; Wolfson, Abbie; Kelley, Margaret | 1992 | Cognitive improvement following benzodiazepine discontinuation in elderly nursing home residents | International journal of geriatric psychiatry |
| Sampath, G.; Shah, A.; Krska, J.; Soni, S. D. | 1992 | Neuroleptic discontinuation in the very stable schizophrenic patient: Relapse rates and serum neuroleptic levels | Human Psychopharmacology: Clinical and Experimental |
| Takata, Y.; Yoshizumi, T.; Ito, Y.; Ueno, M.; Tsukashima, A.; Iwase, M.; Kobayashi, K.; Fujishima, M. | 1992 | Comparison of withdrawing antihypertensive therapy between diuretics and angiotensin converting enzyme inhibitors in essential hypertensives | American heart journal |
| Aldenkamp A.P.; Alpherts W.C.J.; Blennow G.; Elmqvist D.; Heijbel J.; Nilsson H.L.; Sandstedt P.; Tonnby B.; Wahlander L.; Wosse E. | 1993 | Withdrawal of antiepileptic medication in children - Effects on cognitive function: The multicenter Holmfrid study | Neurology |
| Angervo M.; Toivonen J.; Leinonen P.; Valimaki M.; Seppala M. | 1993 | Thyroxine withdrawal is accompanied by decreased circulating levels of insulin-like growth factor-binding protein-1 in thyroidectomized patients | J. Clin. Endocrinol. Metab. |
| Berrios X.; Del Campo E.; Guzman B.; Bisno A.L. | 1993 | Discontinuing rheumatic fever prophylaxis in selected adolescents and young adults: A prospective study | Ann. Intern. Med. |
| Chataway, J.; Fowler, A.; Thompson, P. J.; Duncan, J. S. | 1993 | Discontinuation of clonazepam in patients with active epilepsy | Seizure |
| Dixon L.; Thaker G.; Conley R.; Ross D.; Cascella N.; Tamminga C. | 1993 | Changes in psychopathology and dyskinesia after neuroleptic withdrawal in a double-blind design | Schizophr. Res. |
| Dupont A.; Gomez J.-L.; Cusan L.; Koutsilieris M.; Labrie F. | 1993 | Response to flutamide withdrawal in advanced prostate cancer in progression under combination therapy | J. Urol. |
| Faedda, G. L.; Tondo, L.; Baldessarini, R. J.; Suppes, T.; Tohen, M. | 1993 | Outcome after rapid vs gradual discontinuation of lithium treatment in bipolar disorders | Archives of general psychiatry |
| Ingulli E.; Tejani A.; Markell M. | 1993 | The beneficial effects of steroid withdrawal on blood pressure and lipid profile in children posttransplantation in the cyclosporine era | Transplantation |
| Ingulli, E.; Sharma, V.; Singh, A.; Suthanthiran, M.; Tejani, A. | 1993 | Steroid withdrawal, rejection and the mixed lymphocyte reaction in children after renal transplantation | Kidney international. Supplement |
| Kendrick, Anna M.; Duncan, John S.; Trimble, Michael R. | 1993 | Effects of discontinuation of individual antiepileptic drugs on mood | Human Psychopharmacology: Clinical and Experimental |
| Kirsten D.K.; Wegner R.E.; Jorres R.A.; Magnussen H. | 1993 | Effects of theophylline withdrawal in severe chronic obstructive pulmonary disease | Chest |
| Kocsis, James H.; Shaw, Eric D.; Stokes, Peter E.; Wilner, Philip; Elliot, Alan S.; Sikes, Carolyn; Myers, Barnett; Manevitz, Alan; Parides, Michael | 1993 | Neuropsychologic effects of lithium discontinuation | Journal of clinical psychopharmacology |
| Lader, M.; Farr, I.; Morton, S. | 1993 | A comparison of alpidem and placebo in relieving benzodiazepine withdrawal symptoms | International clinical psychopharmacology |
| Packer, M.; Gheorghiade, M.; Young, J. B.; Costantini, P. J.; Adams, K. F.; Cody, R. J.; Smith, L. K.; van Voorhees, L.; Gourley, L. A.; Jolly, M. K. | 1993 | Withdrawal of digoxin from patients with chronic heart failure treated with angiotensin-converting-enzyme inhibitors. RADIANCE Study | The New England journal of medicine |
| Scher H.I.; Kelly W.K. | 1993 | Flutamide withdrawal syndrome: Its impact on clinical trials in hormone- refractory prostate cancer | J. Clin. Oncol. |
| Sugai, K. | 1993 | Seizures with clonazepam: discontinuation and suggestions for safe discontinuation rates in children | Epilepsia |
| Sullivan, Mark; Toshima, Michelle; Lynn, Pamela; Roy-Byrne, Peter | 1993 | Phenobarbital versus clonazepam for sedative-hypnotic taper in chronic pain patients: A pilot study | Annals of Clinical Psychiatry |
| Takata Y. | 1993 | Which antihypertensive drug to withdraw and when | CARDIOL. BOARD REV. |
| Uretsky, B. F.; Young, J. B.; Shahidi, F. E.; Yellen, L. G.; Harrison, M. C.; Jolly, M. K. | 1993 | Randomized study assessing the effect of digoxin withdrawal in patients with mild to moderate chronic congestive heart failure: results of the PROVED trial. PROVED Investigative Group | Journal of the American College of Cardiology |
| van Herwaarden, G.; Berger, H. J.; Horstink, M. W. | 1993 | Short-term memory in Parkinson's disease after withdrawal of long-term anticholinergic therapy | Clinical neuropharmacology |
| Walma, E. P.; Hoes, A. W.; Prins, A.; Boukes, F. S.; van der Does, E. | 1993 | Withdrawing long-term diuretic therapy in the elderly: a study in general practice in The Netherlands | Family medicine |
| Westhuyzen J.; Matherson K.; Tracey R.; Fleming S.J. | 1993 | Effect of withdrawal of folic acid supplementation in maintenance hemodialysis patients | CLIN. NEPHROL. |
| Canter C.E.; Moorhead S.; Saffitz J.E.; Huddleston C.B.; Spray T.L.; Boucek Jr. R.J.; Dark J.H.; Addonizio L.J. | 1994 | Steroid withdrawal in the pediatric heart transplant recipient initially treated with triple immunosuppression | J. Heart Lung Transplant. |
| De Jonge J.W.; Knottnerus J.A.; Van Zutphen W.M.; De Bruijne G.A.; Struijker Boudier H.A.J. | 1994 | Short term effect of withdrawal of diuretic drugs prescribed for ankle oedema | BR. MED. J. |
| Del Brutto O.H. | 1994 | Prognostic factors for seizure recurrence after withdrawal of antiepileptic drugs in patients with neurocysticercosis | Neurology |
| Fabrega A.J.; Cohan J.; Meslar P.; Pollack R.; Belzer F.O.; Turcotte J.G. | 1994 | Effects of steroid withdrawal on long-term renal allograft recipients with posttransplantation diabetes mellitus | Surgery |
| FOTHERBY, M. D.; POTTER, J. F. | 1994 | POSSIBILITIES FOR ANTIHYPERTENSIVE DRUG-THERAPY WITHDRAWAL IN THE ELDERLY | Journal of human hypertension |
| Grinstead W.C.; Francis M.J.; Marks G.F.; Tawa C.B.; Zoghbi W.A.; Young J.B. | 1994 | Discontinuation of chronic diuretic therapy in stable congestive heart failure secondary to coronary artery disease or to idiopathic dilated cardiomyopathy | Am. J. Cardiol. |
| HO, G. Y.F.; BLAUFOX, M. D.; WASSERTHEILSMOLLER, S.; OBERMAN, A.; LANGFORD, H. | 1994 | PLASMA-RENIN PREDICTS SUCCESS OF ANTIHYPERTENSIVE DRUG-WITHDRAWAL | American journal of hypertension |
| Julian B.A.; Gaston R.S.; Barker C.V.; Krystal G.; Diethelm A.G.; Curtis J.J. | 1994 | Erythropoiesis after withdrawal of enalapril in post-transplant erythrocytosis | Kidney Int |
| Klompmaker I.J.; Homan Van der Heide J.J.; Tegzess A.M.; Meijer S.; Haagsma E.B.; Verwer R.; Slooff M.J.H. | 1994 | Effects of cyclosporin A withdrawal on renal function and renal stimulation in liver transplant patients treated with triple-drug immunosuppression for over 2 years | Nephrol. Dial. Transplant. |
| MORGAN, T.; HOPPER, J.; ANDERSON, A.; CARRICKS, L.; JONES, E.; JOHNS, J.; GREEN, R.; NOWSON, C. | 1994 | CAN DRUG-THERAPY BE STOPPED IN ELDERLY HYPERTENSIVE PATIENTS | CARDIOLOGY IN THE ELDERLY |
| Mukamel, E.; Servadio, C. | 1994 | Rapid tumour recurrence following cessation of long-term treatment with intravesical thiotepa | British journal of urology |
| Nadal M.; Wikstrom L.; Allgulander S. | 1994 | Once hypertensive, always hypertensive? A three year follow-up after stopping medication | SCAND. J. PRIM. HEALTH CARE |
| Shinnar, S.; Berg, A. T.; MOSHE, S. L.; KANG, H.; ODELL, C.; ALEMANY, M.; GOLDENSOHN, E. S.; HAUSER, W. A. | 1994 | DISCONTINUING ANTIEPILEPTIC DRUGS IN CHILDREN WITH EPILEPSY - A PROSPECTIVE-STUDY | Annals of Neurology |
| Tennison, M.; Greenwood, R.; Lewis, D.; Thorn, M. | 1994 | Discontinuing antiepileptic drugs in children with epilepsy. A comparison of a six-week and a nine-month taper period | The New England journal of medicine |
| Thapa, Purushottam B.; Meador, Keith G.; Gideon, Patricia; Fought, Randy L.; Ray, W. A. | 1994 | Effects of antipsychotic withdrawal in elderly nursing home residents | Journal of the American Geriatrics Society |
| Tonnby B.; Nilsson H.L.; Aldenkamp A.P.; Alpherts W.C.J.; Blennow G.; Elmqvist D.; Heijbel J.; Sandstedt P.; Wahlander L.; Wosse E. | 1994 | Withdrawal of antiepileptic medication in children Correlation of cognitive function and plasma concentration-The multicentre 'Holmfrid' study | Epilepsy Res |
| Ben Hadj Ali, B.; Dogui, M.; Ben Ammou, S.; Loo, H. | 1995 | Antiparkinson drugs in neuroleptic treatment: comparative study of progressive and abrupt withdrawal | L'Encephale |
| Donati F.; Hassink R.I.; Jung H.; Vassella F. | 1995 | Factors predicting the risk of relapse after antiepileptic drug discontinuation in children with partial seizures | EUR. J. PEDIATR. SUPPL. |
| Falletta J.M.; Woods G.M.; Verter J.I.; Buchanan G.R.; Pegelow C.H.; Iyer R.V.; Miller S.T.; Holbrook C.T.; Kinney T.R.; Vichinsky E.; Becton D.L.; Wang W.; Johnstone H.S.; Wethers D.L.; Reaman G.H.; DeBaun M.R.; Grossman N.J.; Kalinyak K.; Jorgensen J.H.; Bjornson A.; Thomas M.D.; Reid C. | 1995 | Discontinuing penicillin prophylaxis in children with sickle cell anemia | J. Pediatr. |
| Horwitz, Gary J.; Tariot, Pierre N.; Mead, Karen; Cox, Christopher | 1995 | Discontinuation of antipsychotics in nursing home patients with dementia | The American Journal of Geriatric Psychiatry |
| Klompmaker, I. J.; Gouw, A. S.; Haagsma, E. B.; Verwer, R.; Slooff, M. J. | 1995 | Histological and biochemical effects of cyclosporine A withdrawal from a triple drug regimen after over 2 years of treatment in liver transplant patients | Journal of hepatology |
| Landman, J. O.; Hamdy, N. A.; Pauwels, E. K.; Papapoulos, S. E. | 1995 | Skeletal metabolism in patients with osteoporosis after discontinuation of long-term treatment with oral pamidronate | The Journal of clinical endocrinology and metabolism |
| McDiarmid, S. V.; Farmer, D. A.; Goldstein, L. I.; Martin, P.; Vargas, J.; Tipton, J. R.; Simmons, F.; Busuttil, R. W. | 1995 | A randomized prospective trial of steroid withdrawal after liver transplantation | Transplantation |
| Saudek F.; Pelikanova T.; Reneltova I. | 1995 | No effect of cyclosporine withdrawal on glucose metabolism at one year after kidney transplantation | DIABETES NUTR. METAB. CLIN. EXP. |
| Schweizer E.; Case W.G.; Garcia-Espana F.; Greenblatt D.J.; Rickels K. | 1995 | Progesterone co-administration in patients discontinuing long-term benzodiazepine therapy: Effects on withdrawal severity and taper outcome | Psychopharmacology |
| Bendz H.; Sjödin I.; Aurell M. | 1996 | Renal function on and off lithium in patients treated with lithium for 15 years or more. A controlled, prospective lithium-withdrawal study | Nephrol. Dial. Transplant. |
| Cassano, G. B.; Petracca, A.; Borghi, C.; Chiroli, S.; Didoni, G.; Garreau, M. | 1996 | A randomized, double-blind study of alpidem vs placebo in the prevention and treatment of benzodiazepine withdrawl syndrome | European Psychiatry |
| Del Brutto O.H.; Campos X. | 1996 | Discontinuation of antiepileptic drugs in patients with calcified neurocysticercosis | J. EPILEPSY |
| Delgado M.R.; Riela A.R.; Mills J.; Pitt A.; Browne R. | 1996 | Discontinuation of antiepileptic drug treatment after two seizure-free years in children with cerebral palsy | Pediatrics |
| Goldman M.H.; Davis B.; Cruz E.; Miller P.; Stevens S.L.; Freeman M.B.; Tyler J.D. | 1996 | Effects of azathioprine withdrawal in kidney recipients with stable function two years after transplant | Clin. Transplant. |
| Gøtzsche P.C.; Hansen M.; Stoltenberg M.; Svendsen A.; Beier J.; Faarvang K.L.; Wangel M.; Rydgren L.; Halberg P.; Juncker P.; Andersen V.; Hansen T.M.; Endahl L. | 1996 | Randomized, placebo controlled tial of withdrawal of slow-acting antirheumatic drugs and of observer bias in rheumatoid arthritis | Scand. J. Rheumatol. |
| Herrada J.; Dieringer P.; Logothetis C.J. | 1996 | Characterization of patients with androgen-independent prostatic carcinoma whose serum prostate specific antigen decreased following flutamide withdrawal | J. Urol. |
| Otto G.; Lohse A.; Hofmann W.J.; Golling M. | 1996 | Dose reduction and withdrawal of cyclosporine A in patients following liver transplantation | Cyclosporinreduktion bei Patienten nach Lebertransplantation |
| Ratcliffe, P. J.; Dudley, C. R.; Higgins, R. M.; Firth, J. D.; Smith, B.; Morris, P. J. | 1996 | Randomised controlled trial of steroid withdrawal in renal transplant recipients receiving triple immunosuppression | Lancet (London, England) |
| Riedel, Brant William | 1996 | A comparison of the efficacy of stimulus control for medicated and non-medicated insomniacs | Dissertation Abstracts International: Section B: The Sciences and Engineering |
| Riva D.; Devoti M. | 1996 | Discontinuation of phenobarbital in children: Effects on neurocognitive behavior | Pediatr. Neurol. |
| Somani S.K.; Engberg K.D.; Guay D.R.P. | 1996 | Dyskinesias secondary to gradual neuroleptic drug withdrawal in elderly nursing home residents | J. GERIATR. DRUG THER. |
| Wolde, S. ten; Breedveld, F. C.; Hermans, J.; Vandenbroucke, J. P.; van de Laar, M A; Markusse, H. M.; Janssen, M.; van den Brink, H R; Dijkmans, B. A. | 1996 | Randomised placebo-controlled study of stopping second-line drugs in rheumatoid arthritis | Lancet (London, England) |
| Adams Jr. K.F.; Gheorghiade M.; Uretsky B.F.; Young J.B.; Ahmed S.; Tomasko L.; Packer M. | 1997 | Patients with mild heart failure worsen during withdrawal from digoxin therapy | J. Am. Coll. Cardiol. |
| Beik A.I.; Higgins R.M.; Lam F.T.; Morris A.G. | 1997 | Steroid withdrawal and donor-specific hyporeactivity after cadaveric renal allotransplantation on maintenance triple therapy | Nephrol. Dial. Transplant. |
| Grinyo J.M.; Gil-Vernet S.; Serón D.; Cruzado J.M.; Moreso F.; Fulladosa X.; Castelao A.M.; Torras J.; Hooftman L.; Alsina J. | 1997 | Steroid withdrawal in mycophenolate mofetil-treated renal allograft recipients | Transplantation |
| Habraken H.; Soenen K.; Blondeel L.; Van Elsen J.; Bourda J.; Coppens E.; Willeput M. | 1997 | Gradual withdrawal from benzodiazepines in residents of homes for the elderly: Experience and suggestions for future research | Eur. J. Clin. Pharmacol. |
| Hardy, B. G.; Shulman, K. I.; Zucchero, C. | 1997 | Gradual discontinuation of lithium augmentation in elderly patients with unipolar depression | Journal of clinical psychopharmacology |
| Hollander, A. A.; Hene, R. J.; Hermans, J.; van Es, L. A.; van der Woude, F J | 1997 | Late prednisone withdrawal in cyclosporine-treated kidney transplant patients: a randomized study | Journal of the American Society of Nephrology : JASN |
| Nyberg, S.; Farde, L.; Halldin, C. | 1997 | Delayed normalization of central D2 dopamine receptor availability after discontinuation of haloperidol decanoate. Preliminary findings | Archives of general psychiatry |
| Ruoff, G. E. | 1997 | Sustained normotension in hypertensive patients withdrawn from medication for 1 year | Family medicine |
| Sanfey H.; Haussman G.; Isaacs I.; Ishitani M.; Lobo P.; McCullough C.; Pruett T. | 1997 | Steroid withdrawal in kidney transplant recipients: Is it a safe option? | Clin. Transplant. |
| Stegall M.D.; Everson G.T.; Schroter G.; Karrer F.; Bilir B.; Sternberg T.; Shrestha R.; Wachs M.; Kam I. | 1997 | Prednisone withdrawal late after adult liver transplantation reduces diabetes, hypertension, and hypercholesterolemia without causing graft loss | HEPATOLOGY |
| Tardy B.; Tardy-Poncet B.; Laporte-Simitsidis S.; Mismetti P.; Decousus H.; Guyotat D.; Bertrand J.C. | 1997 | Evolution of blood coagulation and fibrinolysis parameters after abrupt versus gradual withdrawal of acenocoumarol in patients with venous thromboembolism: A double-blind randomized study | Br. J. Haematol. |
| Walma E.P.; Hoes A.W.; Van Dooren C.; Prins A.; Van der Does E. | 1997 | Withdrawal of long term diuretic medication in elderly patients: A double blind randomised trial | BR. MED. J. |
| Dawson, N.; Figg, W. D.; Brawley, O. W.; Bergan, R.; Cooper, M. R.; Senderowicz, A.; Headlee, D.; Steinberg, S. M.; Sutherland, M.; Patronas, N.; Sausville, E.; Linehan, W. M.; Reed, E.; Sartor, O. | 1998 | Phase II study of suramin plus aminoglutethimide in two cohorts of patients with androgen-independent prostate cancer: simultaneous antiandrogen withdrawal and prior antiandrogen withdrawal | Clinical cancer research : an official journal of the American Association for Cancer Research |
| Devlin J.; Doherty D.; Wong T.; Donaldson P.; Portmann B.; Williams R. | 1998 | Defining the outcome of immunosuppression withdrawal after liver transplantation | HEPATOLOGY |
| Gomez, R.; Moreno, E.; Colina, F.; Loinaz, C.; Gonzalez-Pinto, I.; Lumbreras, C.; Perez-Cerda, F.; Castellon, C.; Garcia, I. | 1998 | Steroid withdrawal is safe and beneficial in stable cyclosporine-treated liver transplant patients | Journal of hepatology |
| Hantouche E.G.; Guelfi J.D.; Comet D. | 1998 | Discontinuation of long-term benzodiazepine use: Double-blind controlled study of α-β L-aspartate magnesium versus placebo in 144 chronic users of BZD | REAJUSTEMENT DES THERAPEUTIQUES ANTICONVULSIVES CHEZ L'ENFANT. CONTROLE PAR L'ETUDE ELECTROPHYSIOLOGIQUE DU SOMMEIL DES EFFETS DU VALPROATE DE SODIUM APRES SEVRAGE BARBITURIQUE |
| Jabs, D. A.; Bolton, S. G.; Dunn, J. P.; Palestine, A. G. | 1998 | Discontinuing anticytomegalovirus therapy in patients with immune reconstitution after combination antiretroviral therapy | American journal of ophthalmology |
| Leger J.; Garel C.; Fjellestad-Paulsen A.; Hassan M.; Czernichow P. | 1998 | Human growth hormone treatment of short-stature children born small for gestational age: Effect on muscle and adipose tissue mass during a 3-year treatment period and after 1 year's withdrawal | J. Clin. Endocrinol. Metab. |
| Macdonald, J. C.; Torriani, F. J.; Morse, L. S.; Karavellas, M. P.; Reed, J. B.; Freeman, W. R. | 1998 | Lack of reactivation of cytomegalovirus (CMV) retinitis after stopping CMV maintenance therapy in AIDS patients with sustained elevations in CD4 T cells in response to highly active antiretroviral therapy | The Journal of infectious diseases |
| Marcus, J. C. | 1998 | Stopping antiepileptic therapy in mentally-retarded, epileptic children | Neuropediatrics |
| Nishiyama, T.; Terunuma, M. | 1998 | Hormone/antihormone withdrawal and dexamethasone for hormone-refractory prostate cancer | International journal of urology : official journal of the Japanese Urological Association |
| Romach, Myroslava K.; Kaplan, Howard L.; Busto, Usoa E.; Somer, Gail; Sellers, Edward M. | 1998 | A controlled trial of ondansetron, a 5-HTâ‚ƒ antagonist, in benzodiazepine discontinuation | Journal of clinical psychopharmacology |
| Vrabec T.R.; Baldassano V.F.; Whitcup S.M. | 1998 | Discontinuation of maintenance therapy in patients with quiescent cytomegalovirus retinitis and elevated CD4+ counts | Ophthalmology |
| Campbell, A. John; Robertson, M. Clare; Gardner, Melinda M.; Norton, Robyn N.; Buchner, David M. | 1999 | Psychotropic medication withdrawal and a home-based exercise program to prevent falls: A randomized, controlled trial | Journal of the American Geriatrics Society |
| Churchyard A.; Mathias C.J.; Lees A.J. | 1999 | Selegiline-induced postural hypotension in Parkinson's disease: A longitudinal study on the effects of drug withdrawal | Mov. Disord. |
| Cowan F.J.; Evans W.D.; Gregory J.W. | 1999 | Metabolic effects of discontinuing growth hormone treatment | ARCH. DIS. CHILD. |
| Flint A.J.; Rifat S.L. | 1999 | Recurrence of first-episode geriatric depression after discontinuation of maintenance antidepressants | AM. J. PSYCHIATRY |
| Furrer, H.; Egger, M.; Opravil, M.; Bernasconi, E.; Hirschel, B.; Battegay, M.; Telenti, A.; Vernazza, P. L.; Rickenbach, M.; Flepp, M.; Malinverni, R. | 1999 | Discontinuation of primary prophylaxis against Pneumocystis carinii pneumonia in HIV-1-infected adults treated with combination antiretroviral therapy. Swiss HIV Cohort Study | The New England journal of medicine |
| Garfinkel D.; Zisapel N.; Wainstein J.; Laudon M. | 1999 | Facilitation of benzodiazepine discontinuation by melatonin: A new clinical approach | Arch. Intern. Med. |
| Gebremariam, A.; Mengesha, W.; Enqusilassie, F. | 1999 | Discontinuing anti-epileptic medication(s) in epileptic children: 18 versus 24 months | Annals of tropical paediatrics |
| Gellner, R.; Stange, M.; Schiemann, U.; Domschke, W.; Hengst, K. | 1999 | CRH test prior to discontinuation of long-term low-dose glucocorticoid therapy | Experimental and clinical endocrinology & diabetes : official journal, German Society of Endocrinology [and] German Diabetes Association |
| Hall M.C.; Fritzsch R.J.; Sagalowsky A.I.; Ahrens A.; Petty B.; Roehrborn C.G. | 1999 | Prospective determination of the hormonal response after cessation of luteinizing hormone-releasing hormone agonist treatment in patients with prostate cancer | Urology |
| Johannsson G.; Albertsson-Wikland K.; Bengtsson B.-Å.; Alm J.; Aronson S.; Gustafsson J.; Hagenäs L.; Häger A.; Ivarsson S.; Kriström B.; Marcus C.; Moëll C.; Nilsson K.-O.; Ritzén M.; Tuvemo T.; Westgren U.; Westphal O.; Åman J. | 1999 | Discontinuation of growth hormone (GH) treatment: Metabolic effects in GH-deficient and GH-sufficient adolescent patients compared with control subjects | J. Clin. Endocrinol. Metab. |
| Morimoto S.-I.; Shimizu K.; Yamada K.; Hiramitsu S.; Hishida H. | 1999 | Can β-blocker therapy be withdrawn from patients with dilated cardiomyopathy? | Am. Heart J. |
| Rickels, K.; Schweizer, E.; Espaã±A, F. Garcia; Case, G.; DeMartinis, N.; Greenblatt, D. | 1999 | Trazodone and valproate in patients discontinuing long-term benzodiazepine therapy: Effects on withdrawal symptoms and taper outcome | Psychopharmacology |
| Rickels, Karl; Lucki, Irwin; Schweizer, Edward; Garcã­A-Espaã±A, Felipe; Case, W. George | 1999 | Psychomotor performance of long-term benzodiazepine users before, during, and after benzodiazepine discontinuation | Journal of clinical psychopharmacology |
| Riva D.; Devoti M. | 1999 | Carbamazepine withdrawal in children with previous symptomatic partial epilepsy: Effects on neuropsychologic function | J. Child Neurol. |
| Stewart P.M.; Stewart S.E.; Clark P.M.S.; Sheppard M.C. | 1999 | Clinical and biochemical response following withdrawal of a long- acting, depot injection form of octreotide (Sandostatin-LAR®) | Clin. Endocrinol. |
| Stouthart P.J.H.M.; De Ridder C.M.; Rekers-Mombarg L.T.M.; Delemarre Van Der Waal H.A. | 1999 | Changes in body composition during 12 months after discontinuation of growth hormone therapy in young adults with growth hormone deficiency from childhood | J. Pediatr. Endocrinol. Metab. |
| Taniguchi, H.; Ohki, O.; Yokozeki, H.; Katayama, I.; Tanaka, A.; Kiyosawa, M.; Nishioka, K. | 1999 | Cataract and retinal detachment in patients with severe atopic dermatitis who were withdrawn from the use of topical corticosteroid | The Journal of dermatology |
| Ungvari G.S.; Chiu H.F.K.; Lam L.C.W.; Pang A.H.T.; Chung D.W.S.; Li S.-W.; Chiu S.-N.; Lum F.C.K.; Leung T. | 1999 | Gradual withdrawal of long-term anticholinergic antiparkinson medication in chinese patients with chronic schizophrenia | J. Clin. Psychopharmacol. |
| Van Kraaij D.J.W.; Jansen R.W.M.M.; Bouwels L.H.R.; Hoefnagels W.H.L. | 1999 | Furosemide withdrawal improves postprandial hypotension in elderly patients with heart failure and preserved left ventricular systolic function | Arch. Intern. Med. |
| Whitcup, S. M.; Fortin, E.; Lindblad, A. S.; Griffiths, P.; Metcalf, J. A.; Robinson, M. R.; Manischewitz, J.; Baird, B.; Perry, C.; Kidd, I. M.; Vrabec, T.; Davey, R. T. [JR]; Falloon, J.; Walker, R. E.; Kovacs, J. A.; Lane, H. C.; Nussenblatt, R. B.; Smith, J.; Masur, H.; Polis, M. A. | 1999 | Discontinuation of anticytomegalovirus therapy in patients with HIV infection and cytomegalovirus retinitis | JAMA |
| Best, J.; Althaus, C.; Kersten, A.; Theisen, A.; Gantke, B. | 2000 | Stopping secondary prevention in AIDS patients with inactive CMV retinitis treated with HAART (highly active antiretroviral therapy) | Der Ophthalmologe : Zeitschrift der Deutschen Ophthalmologischen Gesellschaft |
| Chakrabarti P.; Wong H.Y.; Scantlebury V.P.; Jordan M.L.; Vivas C.; Ellis D.; Lombardozzi-Lane S.; Hakala T.R.; Fung J.J.; Simmons R.L.; Starzl T.E.; Shapiro R. | 2000 | Outcome after steroid withdrawal in pediatric renal transplant patients receiving tacrolimus-based immunosuppression | Transplantation |
| Coll P.P.; Abourizk N.N. | 2000 | Successful withdrawal of thyroid hormone therapy in nursing home patients | J. Am. Board Fam. Pract. |
| Hoffmeyer, F.; Hoeper, M. M.; Spiekerkotter, E.; Harringer, W.; Haverich, A.; Fabel, H.; Niedermeyer, J. | 2000 | Azathioprine withdrawal in stable lung and heart/lung recipients receiving cyclosporine-based immunosuppression | Transplantation |
| Jordan M.L.; Chakrabarti P.; Luke P.; Shapiro R.; Vivas C.A.; Scantlebury V.P.; Fung J.J.; Starzl T.E.; Corry R.J. | 2000 | Results of pancreas transplantation after steroid withdrawal under tacrolimus immunosuppression | Transplantation |
| Ketelhut R.G.; Franz I.-W.; Behr U. | 2000 | Long-term discontinuation of oral contraceptives in hypertensive women lowers the blood pressure at rest and during exercise | Langfristiges absetzen oraler kontrazeptiva bei hypertensiven frauen senkt den blutdruck in ruhe und bei belastung |
| Matl, I.; Lacha, J.; Lodererova, A.; Simova, M.; Teplan, V.; Lanska, V.; Vitko, S. | 2000 | Withdrawal of steroids from triple-drug therapy in kidney transplant patients | Nephrology, dialysis, transplantation : official publication of the European Dialysis and Transplant Association - European Renal Association |
| Mayur P.M.; Gangadhar B.N.; Subbakrishna D.K.; Janakiramaiah N. | 2000 | Discontinuation of antidepressant drugs during electroconvulsive therapy: A controlled study | J. Affective Disord. |
| Molinuevo J.L.; Valldeoriola F.; Tolosa E.; Rumiá J.; Valls-Solé J.; Roldan H.; Ferrer E. | 2000 | Levodopa withdrawal after bilateral subthalamic nucleus stimulation in advanced Parkinson disease | Arch. Neurol. |
| Mussini C.; Pezzotti P.; Govoni A.; Borghi V.; Antinori A.; d'Arminio Monforte A.; De Luca A.; Mongiardo N.; Cerri M.C.; Chiodo F.; Concia E.; Bonazzi L.; Moroni M.; Ortona L.; Esposito R.; Cossarizza A.; De Rienzo B. | 2000 | Discontinuation of primary prophylaxis for Pneumocystis carinii pneumonia and toxoplasmic encephalitis in human immunodeficiency virus type I-Infected patients: The changes in opportunistic prophylaxis study | J. Infect. Dis. |
| Nejat R.J.; Rashid H.H.; Bagiella E.; Katz A.E.; Benson M.C. | 2000 | A prospective analysis of time to normalization of serum testosterone after withdrawal of androgen deprivation therapy | J. Urol. |
| Rice K.L.; Rubins J.B.; Lebahn F.; Parenti C.M.; Duane P.G.; Kuskowski M.; Joseph A.M.; Niewoehner D.E. | 2000 | Withdrawal of chronic systemic corticosteroids in patients with COPD: A randomized trial | AM. J. RESPIR. CRIT. CARE MED. |
| Rickels, K.; DeMartinis, N.; Garcia-Espana, F.; Greenblatt, D. J.; Mandos, L. A.; Rynn, M. | 2000 | Imipramine and buspirone in treatment of patients with generalized anxiety disorder who are discontinuing long-term benzodiazepine therapy | The American journal of psychiatry |
| Thervet E.; Morelon E.; Ducloux D.; Bererhi L.; Noël L.H.; Janin A.; Bedrossian J.; Puget S.; Chalopin J.M.; Mihatsch M.; Legendre C.; Kreis H. | 2000 | Cyclosporine withdrawal in stable renal transplant recipients after azathioprine-mycophenolate mofetil conversion | Clin. Transplant. |
| Van Kraaij D.J.W.; Jansen R.W.M.M.; Bouwels L.H.R.; Gribnau F.W.J.; Hoefnagels W.H.L. | 2000 | Furosemide withdrawal in elderly heart failure patients with preserved left ventricular systolic function | Am. J. Cardiol. |
| Verrotti A.; Morresi S.; Basciani F.; Cutarella R.; Morgese G.; Chiarelli F. | 2000 | Discontinuation of anticonvulsant therapy in children with partial epilepsy | Neurology |
| Yangco, B. G.; Bargen, J. C. von; Moorman, A. C.; Holmberg, S. D. | 2000 | Discontinuation of chemoprophylaxis against Pneumocystis carinii pneumonia in patients with HIV infection. HIV Outpatient Study (HOPS) Investigators | Annals of internal medicine |
| Baba, K.; Sakakibara, A.; Yagi, T.; Niwa, S.; Hattori, T.; Koishikawa, I.; Yoshida, K.; Kobayashi, T.; Takagi, K. | 2001 | Effects of theophylline withdrawal in well-controlled asthmatics treated with inhaled corticosteroid | The Journal of asthma : official journal of the Association for the Care of Asthma |
| Caldiroli M.; Cova V.; Lovisolo J.A.; Reali L.; Bono A.V. | 2001 | Antiandrogen withdrawal in the treatment of hormone-relapsed prostate cancer: Single institutional experience | Eur. Urol. |
| Chen Y.-J.; Chi Chow J.; Lee I.-C. | 2001 | Comparison the cognitive effect of anti-epileptic drugs in seizure-free children with epilepsy before and after drug withdrawal | Epilepsy Res |
| Deeks, S. G.; Wrin, T.; Liegler, T.; Hoh, R.; Hayden, M.; Barbour, J. D.; Hellmann, N. S.; Petropoulos, C. J.; McCune, J. M.; Hellerstein, M. K.; Grant, R. M. | 2001 | Virologic and immunologic consequences of discontinuing combination antiretroviral-drug therapy in HIV-infected patients with detectable viremia | The New England journal of medicine |
| Fors H.; Bjarnason R.; Wirén L.; Albertsson-Wikland K.; Bosaeus I.; Bengtsson B.-Å.; Johannsson G. | 2001 | Currently used growth-promoting treatment of children results in normal bone mass and density. A prospective trial of discontinuing growth hormone treatment in adolescents | Clin. Endocrinol. |
| Jouan, M.; Saves, M.; Tubiana, R.; Carcelain, G.; Cassoux, N.; Aubron-Olivier, C.; Fillet, A. M.; Nciri, M.; Senechal, B.; Chene, G.; Tural, C.; Lasry, S.; Autran, B.; Katlama, C. | 2001 | Discontinuation of maintenance therapy for cytomegalovirus retinitis in HIV-infected patients receiving highly active antiretroviral therapy | AIDS (London, England) |
| Klaus G.; Jeck N.; Konrad M.; Förster B.; Soergel M. | 2001 | Risk of steroid withdrawal in pediatric renal transplant patients with suspected steroid toxicity | CLIN. NEPHROL. |
| Koletar S.L.; Heald A.E.; Finkelstein D.; Hafner R.; Currier J.S.; McCutchan J.A.; Vallee M.; Torriani F.J.; Powderly W.G.; Fass R.J.; Murphy R.L. | 2001 | A prospective study of discontinuing primary and secondary Pneumocystis carinii pneumonia prophylaxis after CD4 cell count increase to > 200 × 106/l | AIDS |
| Lerut J.P.; Ciccarelli O.; Mauel E.; Gheerardhyn R.; Talpe S.; Sempoux C.; Laterre P.-F.; Roggen F.M.; Van Leeuw V.; Otte J.-B.; Gianello P. | 2001 | Adult liver transplantation and steroidazathioprine withdrawal in cyclosporine (Sandimmun)-based immunosuppression 5 year results of a prospective study | Transplant Int |
| Lönnkvist K.; Hellman C.; Lundahl J.; Halldén G.; Hedlin G. | 2001 | Eosinophil markers in blood, serum, and urine for monitoring the clinical course in childhood asthma: Impact of budesonide treatment and withdrawal | J. Allergy Clin. Immunol. |
| Lopez Bernaldo de Quiros, J C; Miro, J. M.; Pena, J. M.; Podzamczer, D.; Alberdi, J. C.; Martinez, E.; Cosin, J.; Claramonte, X.; Gonzalez, J.; Domingo, P.; Casado, J. L.; Ribera, E. | 2001 | A randomized trial of the discontinuation of primary and secondary prophylaxis against Pneumocystis carinii pneumonia after highly active antiretroviral therapy in patients with HIV infection. Grupo de Estudio del SIDA 04/98 | The New England journal of medicine |
| Shammas N.W.; Harris M.L.; McKinney D.; Hauber W.J. | 2001 | Digoxin withdrawal in patients with dilated cardiomyopathy following normalization of ejection fraction with beta blockers | Clin. Cardiol. |
| Tremollieres, F. A.; Pouilles, J. M.; Ribot, C. | 2001 | Withdrawal of hormone replacement therapy is associated with significant vertebral bone loss in postmenopausal women | Osteoporosis international : a journal established as result of cooperation between the European Foundation for Osteoporosis and the National Osteoporosis Foundation of the USA |
| Weir, M. R.; Ward, M. T.; Blahut, S. A.; Klassen, D. K.; Cangro, C. B.; Bartlett, S. T.; Fink, J. C. | 2001 | Long-term impact of discontinued or reduced calcineurin inhibitor in patients with chronic allograft nephropathy | Kidney international |
| Whittal M.L.; Otto M.W.; Hong J.J. | 2001 | Cognitive-behavior therapy for discontinuation of SSRI treatment of panic disorder: A case series | BEHAV. RES. THER. |
| Wirén L.; Johannsson G.; Bengtsson B.-Å. | 2001 | A prospective investigation of quality of life and psychological well-being after the discontinuation of GH treatment in adolescent patients WHO had GH deficiency during childhood | J. Clin. Endocrinol. Metab. |
| Aberg, Judith A.; Price, Richard W.; Heeren, Dorie M.; Bredt, Barry | 2002 | A pilot study of the discontinuation of antifungal therapy for disseminated cryptococcal disease in patients with acquired immunodeficiency syndrome, following immunologic response to antiretroviral therapy | The Journal of infectious diseases |
| Abramowicz, Daniel; Manas, Derek; Lao, Mieczyslaw; Vanrenterghem, Yves; Del Castillo, Domingo; Wijngaard, Peter; Fung, Samson | 2002 | Cyclosporine withdrawal from a mycophenolate mofetil-containing immunosuppressive regimen in stable kidney transplant recipients: a randomized, controlled study | Transplantation |
| Akuta N.; Suzuki F.; Tsubota A.; Arase Y.; Suzuki Y.; Someya T.; Kobayashi M.; Saitoh S.; Ikeda K.; Kumada H. | 2002 | Long-term clinical remission induced by corticosteroid withdrawal therapy (CSWT) in patients with chronic hepatitis b infection: A prospective randomized controlled trial - CSWT with and without follow-up interferon-α therapy | Dig. Dis. Sci. |
| Bahçeciler N.N.; Barlan I.B.; Nuhoǧlu Y.; Başaran M.M. | 2002 | Which factors predict success after discontinuation of inhaled budesonide therapy in children with asthma? | J. Asthma |
| Berenguer, Juan; Gonzalez, Juan; Pulido, Federico; Padilla, Belen; Casado, Jose Luis; Rubio, Rafael; Arribas, Jose Rarmon | 2002 | Discontinuation of secondary prophylaxis in patients with cytomegalovirus retinitis who have responded to highly active antiretroviral therapy | Clinical infectious diseases : an official publication of the Infectious Diseases Society of America |
| Boots J.M.M.; Van Duijnhoven E.M.; Christiaans M.H.L.; Wolffenbuttel B.H.R.; Van Hooff J.P. | 2002 | Glucose metabolism in renal transplant recipients on tacrolimus: The effect of steroid withdrawal and tacrolimus trough level reduction | J. Am. Soc. Nephrol. |
| Díaz-Guerra G.M.; Gómez R.; Jódar E.; Loinaz C.; Moreno and E.; Hawkins F. | 2002 | Long-term follow-up of bone mass after orthotopic liver transplantation: Effect of steroid withdrawal from the immunosuppressive regimen | Osteoporosis Int |
| Graniewski-Wijnands H.S.; Van der Torren K. | 2002 | Electro-ophthalmological recovery after withdrawal from vigabatrin | Doc. Ophthalmol. |
| Kostis J.B.; Wilson A.C.; Shindler D.M.; Cosgrove N.M.; Lacy C.R. | 2002 | Persistence of normotension after discontinuation of lifestyle intervention in the trial of TONE | Am. J. Hypertens. |
| Lampit M.; Hochberg Z. | 2002 | Prevention of growth deceleration after withdrawal of growth hormone therapy in idiopathic short stature | J. Clin. Endocrinol. Metab. |
| Lemieux, Isabelle; Houde, Isabelle; Pascot, Agnes; Lachance, Jean-Guy; Noel, Real; Radeau, Thierry; Despres, Jean-Pierre; Bergeron, Jean | 2002 | Effects of prednisone withdrawal on the new metabolic triad in cyclosporine-treated kidney transplant patients | Kidney international |
| Mori K.; Yamashita H.; Nagao M.; Horiguchi J.; Yamawaki S. | 2002 | Effects of anticholinergic drug withdrawal on memory, regional cerebral blood flow and extra-pyramidal side effects in schizophrenic patients | Pharmacopsychiatry |
| Najafi M.R.; Tamizifar B. | 2002 | Drug discontinuation in epileptic children: Predictive value of the EEG | Arch. Iran. Med. |
| Newell K.M.; Ko Y.G.; Sprague R.L.; Mahorney S.L.; Bodfish J.W. | 2002 | Onset of dyskinesia and changes in postural task performance during the course of neuroleptic withdrawal | AM. J. MENT. RETARD. |
| Petrovic M.; Pevernagie D.; Mariman A.; Van Maele G.; Afschrift M. | 2002 | Fast withdrawal from benzodiazepines in geriatric inpatients: A randomised double-blind, placebo-controlled trial | Eur. J. Clin. Pharmacol. |
| Schnuelle, Peter; van der Heide, Jaap Homan; Tegzess, Adam; Verburgh, Cornelis A.; Paul, Leendert C.; van der Woude, Fokko Johannes; Fijter, Johan W. de | 2002 | Open randomized trial comparing early withdrawal of either cyclosporine or mycophenolate mofetil in stable renal transplant recipients initially treated with a triple drug regimen | Journal of the American Society of Nephrology : JASN |
| Sheng W.H.; Hung C.C.; Chen M.Y.; Hsieh S.M.; Chang S.C. | 2002 | Successful discontinuation of fluconazole as secondary prophylaxis for cryptococcosis in AIDS patients responding to highly active antiretroviral therapy | Int. J. STD AIDS |
| Smak Gregoor, Peter J H; de Sevaux, Ruud G L; Ligtenberg, Gerry; Hoitsma, Andries J.; Hene, Ronald J.; Weimar, Willem; Hilbrands, Luuk B.; van Gelder, Teun | 2002 | Withdrawal of cyclosporine or prednisone six months after kidney transplantation in patients on triple drug therapy: a randomized, prospective, multicenter study | Journal of the American Society of Nephrology : JASN |
| Specchio L.M.; Tramacere L.; La Neve A.; Beghi E. | 2002 | Discontinuing antiepileptic drugs in patients who are seizure free on monotherapy | J. Neurol. Neurosurg. Psychiatry |
| van Reekum, Robert; Clarke, Diana; Conn, David; Hermann, Nathan; Eryavec, Goran; Cohen, Tammy; Ostrander, Laurie | 2002 | A randomized, placebo-controlled trial of the discontinuation of long-term antipsychotics in dementia | International Psychogeriatrics |
| Aberg, Judith A.; Williams, Paige L.; Liu, Tun; Lederman, Howard M.; Hafner, Richard; Torriani, Francesca J.; Lennox, Jeffrey L.; Dube, Michael P.; MacGregor, Rob Roy; Currier, Judith S. | 2003 | A study of discontinuing maintenance therapy in human immunodeficiency virus-infected subjects with disseminated Mycobacterium avium complex: AIDS Clinical Trial Group 393 Study Team | The Journal of infectious diseases |
| Bowers, Malcom B. [JR.]; McKay, Bernice G.; Mazure, Carolyn M. | 2003 | Discontinuation of antidepressants in newly admitted psychotic patients | The Journal of neuropsychiatry and clinical neurosciences |
| Curran, H. V.; Collins, R.; Fletcher, S.; Kee, S. C. Y.; Woods, B.; Iliffe, S. | 2003 | Older adults and withdrawal from benzodiazepine hypnotics in general practice: effects on cognitive function, sleep, mood and quality of life | Psychological medicine |
| Egawa S.; Okusa H.; Matsumoto K.; Suyama K.; Baba S. | 2003 | Changes in prostate-specific antigen and hormone levels following withdrawal of prolonged ablation for prostate cancer | Prostate Cancer Prostatic Dis |
| Fledelius H.C. | 2003 | Vigabatrin-associated visual field constriction in a longitudinal series. Reversibility suggested after drug withdrawal | Acta Ophthalmol. Scand. |
| George J.; Kitzis I.; Zandorf D.; Golovner M.; Shapira I.; Laniado S.; Roth A. | 2003 | Safety of Nitrate Withdrawal in Angina-Free and Hemodynamically Stable Patients with Coronary Artery Disease | Chest |
| Iwamoto, Toshihiko; Kin, Kyoko; Miyazaki, Kaori; Shin, Kouichi; Takasaki, Masaru | 2003 | Recovery of platelet function after withdrawal of cilostazol administered orally for a long period | Journal of atherosclerosis and thrombosis |
| Kosch, Markus; Hausberg, Martin; Suwelack, Barbara | 2003 | Studies on effects of calcineurin inhibitor withdrawal on arterial distensibility and endothelial function in renal transplant recipients | Transplantation |
| Kos-Kudła B.; Ciesielska-Kopacz N.; Ostrowska Z.; Marek B.; Kajdaniuk D.; Staszewicz P.; Foltyn W. | 2003 | Adrenal cortex function in asthmatic patients following the discontinuation of chronic therapy with systemic glucocorticosteroids | J. Clin. Pharm. Ther. |
| McMillan, C. V.; Bradley, C.; Gibney, J.; Healy, M. L.; Russell-Jones, D. L.; Sonksen, P. H. | 2003 | Psychological effects of withdrawal of growth hormone therapy from adults with growth hormone deficiency | Clinical endocrinology |
| Mortimer, Ann M.; Martin, Maria; Wheeler Vega, Jason A.; Tyson, Philip J. | 2003 | Conventional antipsychotic prescription in unipolar depression, II: withdrawing conventional antipsychotics in unipolar, nonpsychotic patients | The Journal of clinical psychiatry |
| Mussini C.; Pezzotti P.; Antinori A.; Borghi V.; d'Arminio Monforte A.; Govoni A.; De Luca A.; Ammassari A.; Mongiardo N.; Cerri M.C.; Bedini A.; Beltrami C.; Ursitti M.A.; Bini T.; Cossarizza A.; Esposito R. | 2003 | Discontinuation of secondary prophylaxis for Pneumocystis carinii pneumonia in human immunodeficiency virus-infected patients: A randomized trial by the CIOP Study Group | Clin. Infect. Dis. |
| Nelson; Reid, C. M.; Krum, H.; Ryan, P.; Wing, L. M.H.; McNeil, J. J.; Second Australian Natl Blood Press | 2003 | Short-term predictors of maintenance of normotension after withdrawal of anti hypertensive drugs in the Second Australian National Blood Pressure Study (ANBP2) | American journal of hypertension |
| Oude Voshaar, R. C.; Gorgels, W. J. M. J.; Mol, A. J. J.; Van Balkom, A. J. L. M.; Van de Lisdonk, E. H.; Breteler, M. H. M.; Van den Hoogen, H. J. M.; Zitman, F. G. | 2003 | Tapering off long-term benzodiazepine use with or without group cognitive-behavioural therapy: Three-conditioned, randomised controlled trial | The British Journal of Psychiatry |
| Pons J.A.; Yélamos J.; Ramírez P.; Oliver-Bonet M.; Sánchez A.; Rodríguez-Gago M.; Navarro J.; Bermejo J.; Robles R.; Parrilla P. | 2003 | Endothelial cell chimerism does not influence allograft tolerance in liver transplant patients after withdrawal of immunosuppression | Transplantation |
| Roy-Byrne P.; Russo J.; Pollack M.; Stewart R.; Bystrisky A.; Bell J.; Rosenbaum J.; Corrigan M.H.; Stolk J.; Rush A.J.; Ballenger J. | 2003 | Personality and symptom sensitivity predictors of alprazolam withdrawal in panic disorder | UTILISATION DES MEDICAMENTS BETA-BLOQUANTS DANS LES ETATS DE SEVRAGE |
| Rynn M.; García-España F.; Greenblatt D.J.; Mandos L.A.; Schweizer E.; Rickels K. | 2003 | Imipramine and buspirone in patients with panic disorder who are discontinuing long-term benzodiazepine therapy | J. Clin. Psychopharmacol. |
| Sevinc, C.; Cimrin, A. Hikmet; Ellidokuz, H. | 2003 | Withdrawal of inhaled corticosteroid therapy in long-term, stable, mild to moderate, persistent asthmatic patients | Journal of investigational allergology & clinical immunology |
| Tauber M.; Jouret B.; Cartault A.; Lounis N.; Gayrard M.; Marcouyeux C.; Pienkowski C.; Oliver I.; Moulin P.; Otal P.; Joffre F.; Arnaud C.; Rochiccioli P. | 2003 | Adolescents with Partial Growth Hormone (GH) Deficiency Develop Alterations of Body Composition after GH Discontinuation and Require Follow-Up | J. Clin. Endocrinol. Metab. |
| Ulfvarson, Johanna; Adami, Johanna; Wredling, Regina; Kjellman, Bengt; Reilly, Marie; Bahr, Christer von | 2003 | Controlled withdrawal of selective serotonin reuptake inhibitor drugs in elderly patients in nursing homes with no indication of depression | European journal of clinical pharmacology |
| Van den Ham E.C.H.; Kooman J.P.; Christiaans M.H.L.; Van Hooff J.P. | 2003 | The influence of early steroid withdrawal on body composition and bone mineral density in renal transplantation patients | Transplant Int |
| Van Kraaija D.J.W.; Jansen R.W.M.M.; Sweep F.C.G.J.; Hoefnagels W.H.L. | 2003 | Neurohormonal effects of furosemide withdrawal in elderly heart failure patients with normal systolic function | Eur. J. Heart Fail. |
| Vibhagool, Asda; Sungkanuparph, Somnuek; Mootsikapun, Piroon; Chetchotisakd, Ploenchan; Tansuphaswaswadikul, Somsit; Bowonwatanuwong, Chureeratana; Ingsathit, Atiporn | 2003 | Discontinuation of secondary prophylaxis for cryptococcal meningitis in human immunodeficiency virus-infected patients treated with highly active antiretroviral therapy: a prospective, multicenter, randomized study | Clinical infectious diseases : an official publication of the Infectious Diseases Society of America |
| Westall C.A.; Nobile R.; Morong S.; Buncic J.R.; Logan W.J.; Panton C.M. | 2003 | Changes in the electroretinogram resulting from discontinuation of vigabatrin in children | Doc. Ophthalmol. |
| Ballard, Clive G.; Thomas, Alan; Fossey, Jane; Lee, Lesley; Jacoby, Robin; Lana, Marisa M.; Bannister, Carol; McShane, Rupert; Swann, Alan; Juszczak, Ed; O'Brien, John T. | 2004 | A 3-month, randomized, placebo-controlled, neuroleptic discontinuation study in 100 people with dementia: the neuropsychiatric inventory median cutoff is a predictor of clinical outcome | The Journal of clinical psychiatry |
| Carroll, P. V.; Drake, W. M.; Maher, K. T.; Metcalfe, K.; Shaw, N. J.; Dunger, D. B.; Cheetham, T. D.; Camacho-Hubner, C.; Savage, M. O.; Monson, J. P. | 2004 | Comparison of continuation or cessation of growth hormone (GH) therapy on body composition and metabolic status in adolescents with severe GH deficiency at completion of linear growth | The Journal of clinical endocrinology and metabolism |
| Drimer, T.; Shahal, B.; Barak, Y. | 2004 | Effects of discontinuation of long-term anticholinergic treatment in elderly schizophrenia patients | International clinical psychopharmacology |
| Goldman, Mitchell; Zackin, Robert; Fichtenbaum, Carl J.; Skiest, Daniel J.; Koletar, Susan L.; Hafner, Richard; Wheat, L. Joseph; Nyangweso, Peter M.; Yiannoutsos, Constantin T.; Schnizlein-Bick, Carol T.; Owens, Susan; Aberg, Judith A. | 2004 | Safety of discontinuation of maintenance therapy for disseminated histoplasmosis after immunologic response to antiretroviral therapy | Clinical infectious diseases : an official publication of the Infectious Diseases Society of America |
| Lönnkvist K.; Anderson M.; Redlin G.; Svartengren M. | 2004 | Exhaled NO and eosinophil markers in blood, nasal lavage and sputum in children with asthma after withdrawal of budesonide | Pediatr. Allergy Immunol. |
| Maher B.; Lloyd J.; Wilkins E.G.L.; Fraser W.D.; Back D.; Park B.K.; Pirmohamed M. | 2004 | Lipodystrophy in patients with HIV-1 infection: Effect of stopping protease inhibitors on TNF-α and TNF-receptor levels, and on metabolic parameters | Antiviral Ther |
| Mercier-Guyon C.; Chabannes J.P.; Saviuc P. | 2004 | The role of captodiamine in the withdrawal from long-term benzodiazepine treatment | Curr. Med. Res. Opin. |
| Miozzari, Marco; Ambuhl, Patrice M. | 2004 | Steroid withdrawal after long-term medication for immunosuppressive therapy in renal transplant patients: adrenal response and clinical implications | Nephrology, dialysis, transplantation : official publication of the European Dialysis and Transplant Association - European Renal Association |
| Morin, Charles M.; Bastien, Cã©Lyne; Guay, Bernard; Radouco-Thomas, Monelly; Leblanc, Jacinthe; Valliã¨Res, Annie | 2004 | Randomized Clinical Trial of Supervised Tapering and Cognitive Behavior Therapy to Facilitate Benzodiazepine Discontinuation in Older Adults With Chronic Insomnia | The American journal of psychiatry |
| Ohta, Hodaka; Ohtsuka, Yoko; Tsuda, Toshihide; Oka, Eiji | 2004 | Prognosis after withdrawal of antiepileptic drugs in childhood-onset cryptogenic localization-related epilepsies | Brain & development |
| Poyares, Dalva; Guilleminault, Christian; Ohayon, Maurice M.; Tufik, Sergio | 2004 | Chronic benzodiazepine usage and withdrawal in insomnia patients | Journal of psychiatric research |
| Ruths, Sabine; Straand, Jã¸Rund; Nygaard, Harald A.; Bjorvatn, Bjã¸Rn; Pallesen, Stã¥Le | 2004 | Effect of Antipsychotic Withdrawal on Behavior and Sleep/Wake Activity in Nursing Home Residents with Dementia: A Randomized, Placebo-Controlled, Double-Blinded Study: The Bergen District Nursing Home Study | Journal of the American Geriatrics Society |
| Saksa, John R.; Baker, C. Bruce; Woods, Scott W. | 2004 | Mood-stabilizer-maintained, remitted bipolar patients: taper and discontinuation of adjunctive antipsychotic medication | General hospital psychiatry |
| Schermer T.R.J.; Hendriks A.J.C.; Chavannes N.H.; Dekhuijzen P.N.R.; Wouters E.F.M.; van den Hoogen H.; Van Schayck C.P.; Van Weel C. | 2004 | Probability and determinants of relapse after discontinuation of inhaled corticosteroids in patients with COPD treated in general practice | Prim. Care Respir. J. |
| Stevenson, Claire; Rajan, Lovely; Reid, Gavin; Melville, Craig; McGilp, Robin; Cooper, Sally-Ann | 2004 | Withdrawal of antipsychotic drugs from adults with intellectual disabilities | Irish Journal of Psychological Medicine |
| Vilien M.; Dahlerup J.F.; Munck L.K.; Nørregaard P.; Grønbæk K.; Fallingborg J. | 2004 | Randomized controlled azathioprine withdrawal after more than two years treatment in Crohn's disease: Increased relapse rate the following year | Aliment. Pharmacol. Ther. |
| Weir M.R.; Blahut S.; Drachenburg C.; Young C.; Papademitriou J.; Klassen D.K.; Cangro C.B.; Bartlett S.T.; Fink J.C. | 2004 | Late calcineurin inhibitor withdrawal as a strategy to prevent graft loss in patients with suboptimal kidney transplant function | AM. J. NEPHROL. |
| Wichniak A.; Brunner H.; Ising M.; Gil F.P.; Holsboer F.; Friess E. | 2004 | Impaired hypothalamic-pituitary-adrenocortical (HPA) system is related to severity of benzodiazepine withdrawal in patients with depression | Psychoneuroendocrinology |
| Yazici, Olcay; Kora, Kaan; Polat, Aslihan; Saylan, Mete | 2004 | Controlled lithium discontinuation in bipolar patients with good response to long-term lithium prophylaxis | Journal of affective disorders |
| Zellweger, Claudine; Opravil, Milos; Bernasconi, Enos; Cavassini, Matthias; Bucher, Heiner C.; Schiffer, Veronique; Wagels, Thomas; Flepp, Markus; Rickenbach, Martin; Furrer, Hansjakob | 2004 | Long-term safety of discontinuation of secondary prophylaxis against Pneumocystis pneumonia: prospective multicentre study | AIDS (London, England) |
| Armanini D.; Scaroni C.; Mattarello M.J.; Fiore C.; Albiger N.; Sartorato P. | 2005 | Idiopathic primary hyperaldosteronism: Normalization of plasma aldosterone after one month withdrawal of long-term therapy with aldosterone-receptor antagonist potassium canrenoate | J. ENDOCRINOL. INVEST. |
| Christiansen, Jens Juel; Fisker, Sanne; Gravholt, Claus Hojbjerg; Bennett, Paul; Svenstrup, Birgit; Andersen, Marianne; Feldt-Rasmussen, Ulla; Christiansen, Jens Sandahl; Jorgensen, Jens Otto Lunde | 2005 | Discontinuation of estrogen replacement therapy in GH-treated hypopituitary women alters androgen status and IGF-I | European journal of endocrinology / European Federation of Endocrine Societies |
| Citterio, F.; Sparacino, V.; Altieri, P.; Rigotti, P.; Calabrese, S.; Poli, M.; Vinti, V.; Segoloni, G. P. | 2005 | Addition of sirolimus to cyclosporine in long-term kidney transplant recipients to withdraw steroid | Transplantation proceedings |
| Fernandez, Hubert H.; Trieschmann, Martha E.; Okun, Michael S. | 2005 | Rebound psychosis: effect of discontinuation of antipsychotics in Parkinson's disease | Movement disorders : official journal of the Movement Disorder Society |
| Galve E.; Mallol A.; Catalan R.; Palet J.; Méndez S.; Nieto E.; Diaz A.; Soler-Soler J. | 2005 | Clinical and neurohumoral consequences of diuretic withdrawal in patients with chronic, stabilized heart failure and systolic dysfunction | Eur. J. Heart Fail. |
| Jackson, Graham; Martin, Emma; McGing, Elaine; Cooper, Alethea | 2005 | Successful withdrawal of oral long-acting nitrates to facilitate phosphodiesterase type 5 inhibitor use in stable coronary disease patients with erectile dysfunction | The journal of sexual medicine |
| Lémann M.; Mary J.-Y.; Colombel J.-F.; Duclos B.; Soule J.-C.; Lerebours E.; Modigliani R.; Bouhnik Y. | 2005 | A randomized, double-blind, controlled withdrawal trial in Crohn's disease patients in long-term remission on azathioprine | Gastroenterology |
| Loucaidou, M.; Borrows, R.; Cairns, T.; Griffith, M.; Hakim, N.; Palmer, A.; Papalois, V.; Taube, D.; McLean, A. G. | 2005 | Late steroid withdrawal for renal transplant recipients on tacrolimus and MMF is safe | Transplantation proceedings |
| Nachman, Sharon; Gona, Philimon; Dankner, Wayne; Weinberg, Adrianna; Yogev, Ram; Gershon, Anne; Rathore, Mobeen; Read, Jennifer S.; Huang, Sharon; Elgie, Carol; Hudgens, Kim; Hughes, Walter | 2005 | The rate of serious bacterial infections among HIV-infected children with immune reconstitution who have discontinued opportunistic infection prophylaxis | Pediatrics |
| Oto, M.; Espie, C.; Pelosi, A.; Selkirk, M.; Duncan, R. | 2005 | The safety of antiepileptic drug withdrawal in patients with non-epileptic seizures | Journal of neurology, neurosurgery, and psychiatry |
| Serra, Jessica Guerra; Montenegro, Maria Augusta; Guerreiro, Marilisa M. | 2005 | Antiepileptic drug withdrawal in childhood: does the duration of tapering off matter for seizure recurrence? | Journal of child neurology |
| Van Der Mast B.J.; Rischen-Vos J.; De Kuiper P.; Vaessen L.M.B.; van Besouw N.M.; Weimar W. | 2005 | Calcineurin inhibitor withdrawal in stable kidney transplant patients decreases the donor-specific cytotoxic T lymphocyte precursor frequency | Transplantation |
| Weston R.; Hussain A.; George E.; Parr N.J. | 2005 | Testosterone recovery and changes in bone mineral density after stopping long-term luteinizing hormone-releasing hormone analogue therapy in osteoporotic patients with prostate cancer | BJU Int |
| Wohl D.A.; Kendall M.A.; Owens S.; Holland G.; Nokta M.; Spector S.A.; Schrier R.; Fiscus S.; Davis M.; Jacobson M.A.; Currier J.S.; Squires K.; Alston-Smith B.; Andersen J.; Freeman W.R.; Higgins M.; Torriani F.J. | 2005 | The safety of discontinuation of maintenance therapy for cytomegalovirus (CMV) retinitis and incidence of immune recovery uveitis following potent antiretroviral therapy | HIV Clin. Trials |
| Aktekin, Berrin; Dogan, Ebru Apaydin; Oguz, Yurttas; Senol, Yesim | 2006 | Withdrawal of antiepileptic drugs in adult patients free of seizures for 4 years: a prospective study | Epilepsy & behavior : E&B |
| Angiolillo D.J.; Fernandez-Ortiz A.; Bernardo E.; Ramiŕez C.; Sabaté M.; Jimenez-Quevedo P.; Hernández R.; Moreno R.; Escaned J.; Alfonso F.; Bañuelos C.; Costa M.A.; Bass T.A.; Macaya C. | 2006 | Clopidogrel withdrawal is associated with proinflammatory and prothrombotic effects in patients with diabetes and coronary artery disease | Diabetes |
| Baz-Hecht, M.; Osher, E.; Yachnin, T.; Nakache, R.; Nakache, G.; Tordjman, K.; Stern, N. | 2006 | The low-dose (1 microg) adrenocorticotropin stimulation test in kidney and kidney-pancreas transplant patients: a potential guideline for steroid withdrawal | Clinical transplantation |
| Bertschy S.; Opravil M.; Cavassini M.; Bernasconi E.; Schiffer V.; Schmid P.; Flepp M.; Chave J.-P.; Christen A.; Furrer H. | 2006 | Discontinuation of maintenance therapy against toxoplasma encephalitis in AIDS patients with sustained response to anti-retroviral therapy | Clin. Microbiol. Infect. |
| Bjornsson, E.; Abrahamsson, H.; Simren, M.; Mattsson, N.; Jensen, C.; Agerforz, P.; Kilander, A. | 2006 | Discontinuation of proton pump inhibitors in patients on long-term therapy: a double-blind, placebo-controlled trial | Alimentary pharmacology & therapeutics |
| Farmer, C. K. T.; Hampson, G.; Abbs, I. C.; Hilton, R. M.; Koffman, C. G.; Fogelman, I.; Sacks, S. H. | 2006 | Late low-dose steroid withdrawal in renal transplant recipients increases bone formation and bone mineral density | American journal of transplantation : official journal of the American Society of Transplantation and the American Society of Transplant Surgeons |
| Gleissner C.A.; Doesch A.; Ehlermann P.; Koch A.; Sack F.U.; Katus H.A.; Dengler T.J. | 2006 | Cyclosporine withdrawal improves renal function in heart transplant patients on reduced-dose cyclosporine therapy | Am. J. Transplant. |
| Hausberg M.; Lang D.; Levers A.; Suwelack B.; Kisters K.; Tokmak F.; Barenbrock M.; Kosch M. | 2006 | Sympathetic nerve activity in renal transplant patients before and after withdrawal of cyclosporine | J. Hypertens. |
| Hessen E.; Lossius M.I.; Reinvang I.; Gjerstad L. | 2006 | Influence of major antiepileptic drugs on attention, reaction time, and speed of information processing: Results from a randomized, double-blind, placebo-controlled withdrawal study of seizure-free epilepsy patients receiving monotherapy | Epilepsia |
| Kobayashi T.; Nishizawa K.; Mitsumori K. | 2006 | Individual variation of hormonal recovery after cessation of luteinizing hormone-releasing hormone agonist therapy in men receiving long-term medical castration therapy for prostate cancer | Scand. J. Urol. Nephrol. |
| Michalska, Dana; Stepan, Jan J.; Basson, Bruce R.; Pavo, Imre | 2006 | The effect of raloxifene after discontinuation of long-term alendronate treatment of postmenopausal osteoporosis | The Journal of clinical endocrinology and metabolism |
| Miro, Jose M.; Lopez, Juan C.; Podzamczer, Daniel; Pena, Jose M.; Alberdi, Juan C.; Martinez, Esteban; Domingo, Pere; Cosin, Jaime; Claramonte, Xavier; Arribas, Jose R.; Santin, Miguel; Ribera, Esteban | 2006 | Discontinuation of primary and secondary Toxoplasma gondii prophylaxis is safe in HIV-infected patients after immunological restoration with highly active antiretroviral therapy: results of an open, randomized, multicenter clinical trial | Clinical infectious diseases : an official publication of the Infectious Diseases Society of America |
| Rauch, Frank; Munns, Craig; Land, Christof; Glorieux, Francis H. | 2006 | Pamidronate in children and adolescents with osteogenesis imperfecta: effect of treatment discontinuation | The Journal of clinical endocrinology and metabolism |
| Shinkai N.; Tanaka Y.; Orito E.; Ito K.; Ohno T.; Hirashima N.; Hasegawa I.; Sugauchi F.; Ueda R.; Mizokami M. | 2006 | Measurement of hepatitis B virus core-related antigen as predicting factor for relapse after cessation of lamivudine therapy for chronic hepatitis B virus infection | Hepatol. Res. |
| Sillanpää M.; Schmidt D. | 2006 | Prognosis of seizure recurrence after stopping antiepileptic drugs in seizure-free patients: A long-term population-based study of childhood-onset epilepsy | Epilepsy Behav |
| Tonekaboni, Seyed Hassan; Beyraghi, Narguess; Tahbaz, Hosseinzadeh Sahar; Bahreynian, Seyed Abdolmajid; Aghamohammadpoor, Mehran | 2006 | Neurocognitive effects of phenobarbital discontinuation in epileptic children | Epilepsy & Behavior |
| Tonelli, M.; Bacci, E.; Dente, F. L.; Bartoli, M. L.; Cianchetti, S.; Di Franco, A.; Vagaggini, B.; Zingoni, M.; Paggiaro, P. L. | 2006 | Predictors of symptom recurrence after low-dose inhaled corticosteroid cessation in mild persistent asthma | Respiratory medicine |
| Vicens, Catalina; Fiol, Francisca; Llobera, Joan; Campoamor, Francisco; Mateu, Catalina; Alegret, Santiago; Socias, Isabel | 2006 | Withdrawal from long-term benzodiazepine use: randomised trial in family practice | The British journal of general practice : the journal of the Royal College of General Practitioners |
| Aslan, Erdogan; Bagis, Tayfun; Kilicdag, Esra Bulgan; Tarim, Ebru; Erkanli, Serkan; Kuscu, Esra | 2007 | How best is to discontinue postmenopausal hormone therapy: immediate or tapered? | Maturitas |
| Assy, Nimer; Adams, Paul C.; Myers, Paul; Simon, Verra; Minuk, Gerry Y.; Wall, William; Ghent, Cameron N. | 2007 | Randomized controlled trial of total immunosuppression withdrawal in liver transplant recipients: role of ursodeoxycholic acid | Transplantation |
| Choudhury, Aklak B.; Dawson, Carolyn M.; Kilvington, Hazel E.; Eldridge, Sandra; James, Wai-Yee; Wedzicha, Jadwiga A.; Feder, Gene S.; Griffiths, Chris J. | 2007 | Withdrawal of inhaled corticosteroids in people with COPD in primary care: a randomised controlled trial | Respiratory research |
| Colivicchi F.; Bassi A.; Santini M.; Caltagirone C. | 2007 | Discontinuation of statin therapy and clinical outcome after ischemic stroke | Stroke |
| Gorgels W.J.M.J.; Oude Voshaar R.C.; Mol A.J.J.; Van De Lisdonk E.H.; Mulder J.; van den Hoogen H.; Van Balkom A.J.L.M.; Breteler M.H.M.; Zitman F.G. | 2007 | Consequences of a benzodiazepine discontinuation programme in family practice on psychotropic medication prescription to the participants | Fam. Pract. |
| Hessen E.; Lossius M.I.; Reinvang I.; Gjerstad L. | 2007 | Influence of major antiepileptic drugs on neuropsychological function: Results from a randomized, double-blind, placebo-controlled withdrawal study of seizure-free epilepsy patients on monotherapy | J. Int. Neuropsychol. Soc. |
| Hessen, Erik; Lossius, Morten I.; Reinvang, Ivar; Gjerstad, Leif | 2007 | Slight improvement in mood and irritability after antiepileptic drug withdrawal: A controlled study in patients on monotherapy | Epilepsy & Behavior |
| Kennebäck G.; Tabrizi F.; Lindell P.; Nordlander R. | 2007 | High-degree atrioventricular block during anti-arrhythmic drug treatment: Use of a pacemaker with a bradycardia-detection algorithm to study the time course after drug withdrawal | Europace |
| Kim K.W.; Park Y.J.; Kim T.Y.; Park D.J.; Park K.S.; Cho B.Y. | 2007 | Susceptible alleles of the CD40 and CTLA-4 genes are not associated with the relapse after antithyroid withdrawal in Graves' disease | THYROID |
| Legendre C.; Brault Y.; Morales J.M.; Oberbauer R.; Altieri P.; Riad H.; Mahony J.; Messina M.; Pussell B.; Martínez J.G.; Lelong M.; Burke J.T.; Neylan J.K.F. | 2007 | Factors influencing glomerular filtration rate in renal transplantation after cyclosporine withdrawal using sirolimus-based therapy: A multivariate analysis of results at five years | Clin. Transplant. |
| Lossius M.I.; Erikssen J.E.; Mowinckel P.; Gulbrandsen P.; Gjerstad L. | 2007 | Changes in autonomic cardiac control in patients with epilepsy after discontinuation of antiepileptic drugs: A randomized controlled withdrawal study | Eur. J. Neurol. |
| Lossius M.I.; Taubøll E.; Mowinckel P.; Mørkrid L.; Gjerstad L. | 2007 | Reversible effects of antiepileptic drugs on reproductive endocrine function in men and women with epilepsy - A prospective randomized double-blind withdrawal study | Epilepsia |
| Manosuthi, Weerawat; Ruxrungtham, Kiat; Likanonsakul, Sirirat; Prasithsirikul, Wisit; Inthong, Yaowarat; Phoorisri, Thanongsri; Sungkanuparph, Somnuek | 2007 | Nevirapine levels after discontinuation of rifampicin therapy and 60-week efficacy of nevirapine-based antiretroviral therapy in HIV-infected patients with tuberculosis | Clinical infectious diseases : an official publication of the Infectious Diseases Society of America |
| Nyback-Nakell, A.; Adamson, U.; Lins, P. E.; Landstedt-Hallin, L. | 2007 | Glycaemic responsiveness to long-term insulin plus sulphonylurea therapy as assessed by sulphonylurea withdrawal | Diabetic medicine : a journal of the British Diabetic Association |
| Shen B.; Fazio V.W.; Remzi F.H.; Bennett A.E.; Lopez R.; Lavery I.C.; Brzezinski A.; Sherman K.K.; Lashner B.A. | 2007 | Effect of withdrawal of nonsteroidal anti-inflammatory drug use on ileal pouch disorders | Dig. Dis. Sci. |
| Tengstrand, B.; Larsson, E.; Klareskog, L.; Hafstrom, I. | 2007 | Randomized withdrawal of long-term prednisolone treatment in rheumatoid arthritis: effects on inflammation and bone mineral density | Scandinavian journal of rheumatology |
| Van Der Velde N.; Stricker B.H.Ch.; Pols H.A.P.; Van Der Cammen T.J.M. | 2007 | Risk of falls after withdrawal of fall-risk-increasing drugs: A prospective cohort study | Br. J. Clin. Pharmacol. |
| van der Velde, Nathalie; Stricker, Bruno H. Ch.; Pols, Huibert A. P.; van der Cammen, Tischa J. M. | 2007 | Withdrawal of fall-risk-increasing drugs in older persons: Effect on mobility test outcomes | Drugs & aging |
| van der Velde, Nathalie; van den Meiracker, Anton H.; Pols, Huibert A. P.; Stricker, Bruno H. Ch.; van der Cammen, Tischa J. M. | 2007 | Withdrawal of fall-risk-increasing drugs in older persons: Effect on tilt table test outcomes | Journal of the American Geriatrics Society |
| Vissers F.H.J.A.; Knipschild P.G.; Crebolder H.F.J.M. | 2007 | Is melatonin helpful in stopping the long-term use of hypnotics? A discontinuation trial | PHARM. WORLD SCI. |
| Waib, Luis Fernando; Bonon, Sandra Helena Alves; Salles, Angela Christina; Benard, Gil; de Oliveira, Augusto Cesar Penalva; Pannuti, Claudio Sergio; Pedro, Rogerio de Jesus; Costa, Sandra Cecilia Botelho | 2007 | Withdrawal of maintenance therapy for cytomegalovirus retinitis in AIDS patients exhibiting immunological response to HAART | Revista do Instituto de Medicina Tropical de Sao Paulo |
| Wunderink L.; Nienhuis F.J.; Sytema S.; Slooff C.J.; Knegtering R.; Wiersma D. | 2007 | Guided discontinuation versus maintenance treatment in remitted first-episode psychosis: Relapse rates and functional outcome | J. CLIN. PSYCHIATRY |
| Ballard C.; Lana M.M.; Theodoulou M.; Douglas S.; McShane R.; Jacoby R.; Kossakowski K.; Yu L.-M.; Juszczak E. | 2008 | A randomised, blinded, placebo-controlled trial in dementia patients continuing or stopping neuroleptics (The DART-AD Trial) | PLoS Med |
| Bergh, Sverre; Engedal, Knut | 2008 | The withdrawal of antipsychotics and antidepressants from patients with dementia and BPSD living in nursing homes--An open pilot study | International journal of geriatric psychiatry |
| Greenspan S.L.; Nelson J.B.; Trump D.L.; Wagner J.M.; Miller M.E.; Perera S.; Resnick N.M. | 2008 | Skeletal health after continuation, withdrawal, or delay of alendronate in men with prostate cancer undergoing androgen-deprivation therapy | J. Clin. Oncol. |
| Groetzner, Jan; Kaczmarek, Ingo; Schirmer, Johannes; Uberfuhr, Peter; Gulbins, Helmut; Daebritz, Sabine; Meiser, Bruno; Reichart, Bruno | 2008 | Calcineurin inhibitor withdrawal and conversion to mycophenolate mofetil and steroids in cardiac transplant recipients with chronic renal failure: a word of caution | Clinical transplantation |
| Guran, T.; Ersu, R.; Karadag, B.; Karakoc, F.; Demirel, G. Y.; Hekim, N.; Dagli, E. | 2008 | Withdrawal of inhaled steroids in children with non-cystic fibrosis bronchiectasis | Journal of clinical pharmacy and therapeutics |
| Hessen E.; Lossius M.I.; Reinvang I.; Gjerstad L. | 2008 | Improvement in speeded cognitive processing after anti-epileptic drug withdrawal - A controlled study in mono-therapy patients | Prog. Neurotherapeutics Neuropsychopharmacology |
| Lossius, Morten Ingvar; Hessen, Erik; Mowinckel, Petter; Stavem, Knut; Erikssen, Jan; Gulbrandsen, Paal; Gjerstad, Leif | 2008 | Consequences of antiepileptic drug withdrawal: a randomized, double-blind study (Akershus Study) | Epilepsia |
| Ronchi C.L.; Rizzo E.; Lania A.G.; Pivonello R.; Grottoli S.; Colao A.; Ghigo E.; Spada A.; Arosio M.; Beck-Peccoz P. | 2008 | Preliminary data on biochemical remission of acromegaly after somatostatin analogs withdrawal | Eur. J. Endocrinol. |
| Ruths, Sabine; Straand, Jã¸Rund; Nygaard, Harald A.; Aarsland, Dag | 2008 | Stopping antipsychotic drug therapy in demented nursing home patients: A randomized, placebo-controlled study--The Bergen District Nursing Home Study (BEDNURS) | International journal of geriatric psychiatry |
| Sjoblom, Peter; AndersTengblad; Lofgren, Ulla-Britt; Lannering, Christina; Anderberg, Niklas; Rosenqvist, Ulf; Molstad, Sigvard; Ostgren, Carl J. | 2008 | Can diabetes medication be reduced in elderly patients? An observational study of diabetes drug withdrawal in nursing home patients with tight glycaemic control | Diabetes research and clinical practice |
| Tse W.; Frisina P.G.; Hälbig T.D.; Gracies J.-M.; Liang L.; Tarshish C.; Lesser G.; Neufeld R.; Koller W.C.; Libow L.S. | 2008 | The Effects of Withdrawal of Dopaminergic Medication in Nursing Home Patients With Advanced Parkinsonism | J. Am. Med. Dir. Assoc. |
| Tsujimura, Akira; Takada, Shingo; Matsuoka, Yasuhiro; Hirai, Toshiaki; Takao, Tetsuya; Miyagawa, Yasushi; Nonomura, Norio; Okuyama, Akihiko | 2008 | Is discontinuation of hormone replacement therapy possible for patients with late-onset hypogonadism? | International journal of urology : official journal of the Japanese Urological Association |
| Van Der Velde N.; Meerding W.J.; Looman C.W.; Pols H.A.P.; Van Der Cammen T.J.M. | 2008 | Cost effectiveness of withdrawal of fall-risk-increasing drugs in geriatric outpatients | Drugs Aging |
| Zhu, Qing-guo; Zhao, Ya-kun; Liu, Wei; Luo, Hui; Qiu, Yu; Gao, Zhi-zhong | 2008 | Two-year observation of a randomized trial on tacrolimus-based therapy with withdrawal of steroids or mycophenolate mofetil after renal transplantation | Chinese medical sciences journal = Chung-kuo i hsueh k'o hsueh tsa chih / Chinese Academy of Medical Sciences |
| Ballard, Clive; Hanney, Maria Luisa; Theodoulou, Megan; Douglas, Simon; McShane, Rupert; Kossakowski, Katja; Gill, Randeep; Juszczak, Edmund; Yu, Ly-Mee; Jacoby, Robin | 2009 | The dementia antipsychotic withdrawal trial (DART-AD): Long-term follow-up of a randomised placebo-controlled trial | The Lancet Neurology |
| Brocq O.; Millasseau E.; Albert C.; Grisot C.; Flory P.; Roux C.-H.; Euller-Ziegler L. | 2009 | Effect of discontinuing TNFα antagonist therapy in patients with remission of rheumatoid arthritis | Jt. Bone Spine |
| Gillard P.; Huurman V.; Van Der Auwera B.; Decallonne B.; Poppe K.; Roep B.O.; Gorus F.; Mathieu C.; Pipeleers D.; Keymeulen B. | 2009 | Graves hyperthyroidism after stopping immunosuppressive therapy in type 1 diabetic islet cell recipients with pretransplant TPO autoantibodies | Diabetes care |
| Hocker, Britta; Weber, Lutz T.; Feneberg, Reinhard; Drube, Jens; John, Ulrike; Fehrenbach, Henry; Pohl, Martin; Zimmering, Miriam; Frund, Stefan; Klaus, Gunter; Wuhl, Elke; Tonshoff, Burkhard | 2009 | Prospective, randomized trial on late steroid withdrawal in pediatric renal transplant recipients under cyclosporine microemulsion and mycophenolate mofetil | Transplantation |
| Huber, Bernd; Bocchicchio, M.; Hauser, I.; Horstmann, V.; Jokeit, G.; May, T.; Meinert, T.; Robertson, E.; Schorlemmer, H.; Schulz, L.; Wagner, W.; Seidel, M. | 2009 | Ambiguous results of an attempt to withdraw barbiturates in epilepsy patients with intellectual disability | Seizure |
| Kerling F.; Pauli E.; Lorber B.; Blümcke I.; Buchfelder M.; Stefan H. | 2009 | Drug withdrawal after successful epilepsy surgery: How safe is it? | Epilepsy Behav |
| Lossius, Morten I.; Taubã¸Ll, Erik; Mowinckel, Petter; Gjerstad, Leif | 2009 | Reversible effects of antiepileptic drugs on thyroid hormones in men and women with epilepsy: A prospective randomized double-blind withdrawal study | Epilepsy & Behavior |
| Pincus T.; Swearingen C.J.; Luta G.; Sokka T. | 2009 | Efficacy of prednisone 1-4 mg/day in patients with rheumatoid arthritis: A randomised, double-blind, placebo controlled withdrawal clinical trial | Ann. Rheum. Dis. |
| Pons, Jose A.; Ramirez, Pablo; Revilla-Nuin, Beatriz; Pascual, Domingo; Baroja-Mazo, Alberto; Robles, Ricardo; Sanchez-Bueno, Francisco; Martinez, Laura; Parrilla, Pascual | 2009 | Immunosuppression withdrawal improves long-term metabolic parameters, cardiovascular risk factors and renal function in liver transplant patients | Clinical transplantation |
| Tong, Louis; Huang, Xiao Ling; Koh, Angeline L. T.; Zhang, Xiaoe; Tan, Donald T. H.; Chua, Wei-Han | 2009 | Atropine for the treatment of childhood myopia: effect on myopia progression after cessation of atropine | Ophthalmology |
| Ahmed A.K.; Kamath N.S.; El Kossi M.; El Nahas A.M. | 2010 | The impact of stopping inhibitors of the renin-angiotensin system in patients with advanced chronic kidney disease | Nephrol. Dial. Transplant. |
| Aidaros M.A.; Siam A.G. | 2010 | Effect of the Duration of Withdrawal of Antiepileptic Drugs on the Risk of Seizure Recurrence in Childhood Epilepsy | Egypt. J. Neurol., Psychiatr. Neurosurg. |
| Baldessarini, Ross J.; Tondo, Leonardo; Ghiani, Carmen; Lepri, Beatrice | 2010 | Illness risk following rapid versus gradual discontinuation of antidepressants | The American journal of psychiatry |
| Calvo, Jose A.; Miskulin, Dana C.; Meyer, Klemens B.; Weiner, Daniel E. | 2010 | Nadir hemoglobin levels after discontinuation of epoetin in hemodialysis patients | Clinical journal of the American Society of Nephrology : CJASN |
| Chen, Eric Y. H.; Hui, Christy L. M.; Lam, May M. L.; Chiu, Cindy P. Y.; Law, C. W.; Chung, Dicky W. S.; Tso, Steve; Pang, Edwin P. F.; Chan, K. T.; Wong, Y. C.; Mo, Flora Y. M.; Chan, Kathy P. M.; Yao, T. J.; Hung, S. F.; Honer, William G. | 2010 | Maintenance treatment with quetiapine versus discontinuation after one year of treatment in patients with remitted first episode psychosis: Randomised controlled trial | BMJ: British Medical Journal |
| Cunha, E. P.; Azevedo, L. H.; Pompei, L. M.; Strufaldi, R.; Steiner, M. L.; Ferreira, J. A. S.; Peixoto, S.; Fernandes, C. E. | 2010 | Effect of abrupt discontinuation versus gradual dose reduction of postmenopausal hormone therapy on hot flushes | Climacteric : the journal of the International Menopause Society |
| Foell D.; Wulffraat N.; Wedderburn L.R.; Wittkowski H.; Frosch M.; Gerß J.; Stanevicha V.; Mihaylova D.; Ferriani V.; Tsakalidou F.K.; Foeldvari I.; Cuttica R.; Gonzalez B.; Ravelli A.; Khubchandani R.; Oliveira S.; Armbrust W.; Garay S.; Vojinovic J.; Norambuena X.; Gamir M.L.; García-Consuegra J.; Lepore L.; Susic G.; Corona F.; Dolezalova P.; Pistorio A.; Martini A.; Ruperto N.; Roth J. | 2010 | Methotrexate withdrawal at 6 vs 12 months in juvenile idiopathic arthritis in remission a randomized clinical trial | J. Am. Med. Assoc. |
| Garfinkel, Doron; Mangin, Derelie | 2010 | Feasibility study of a systematic approach for discontinuation of multiple medications in older adults: addressing polypharmacy | Archives of internal medicine |
| Ghaemi, S. Nassir; Ostacher, Michael M.; El-Mallakh, Rif S.; Borrelli, David; Baldassano, Claudia F.; Kelley, Mary E.; Filkowski, Megan M.; Hennen, John; Sachs, Gary S.; Goodwin, Frederick K.; Baldessarini, Ross J. | 2010 | Antidepressant discontinuation in bipolar depression: a Systematic Treatment Enhancement Program for Bipolar Disorder (STEP-BD) randomized clinical trial of long-term effectiveness and safety | The Journal of clinical psychiatry |
| Höcker B.; Weber L.T.; Feneberg R.; Drube J.; John U.; Fehrenbach H.; Pohl M.; Zimmering M.; Fründ S.; Klaus G.; Wühl E.; Tönshoff B. | 2010 | Improved growth and cardiovascular risk after late steroid withdrawal: 2-year results of a prospective, randomised trial in paediatric renal transplantation | Nephrol. Dial. Transplant. |
| Huda M.S.B.; Athauda N.B.; Teh M.M.; Carroll P.V.; Powrie J.K. | 2010 | Factors determining the remission of microprolactinomas after dopamine agonist withdrawal | Clin. Endocrinol. |
| Lašaite L.; Lašiene D.; Lašas L. | 2010 | Cognition, emotions and quality of life in Lithuanian girls with Turner syndrome after growth hormone therapy discontinuation | J. Pediatr. Endocrinol. Metab. |
| Le Cesne, Axel; Ray-Coquard, Isabelle; Bui, Binh Nguyen; Adenis, Antoine; Rios, Maria; Bertucci, Francois; Duffaud, Florence; Chevreau, Christine; Cupissol, Didier; Cioffi, Angela; Emile, Jean-Francois; Chabaud, Sylvie; Perol, David; Blay, Jean-Yves | 2010 | Discontinuation of imatinib in patients with advanced gastrointestinal stromal tumours after 3 years of treatment: an open-label multicentre randomised phase 3 trial | The Lancet. Oncology |
| Lindh-Astrand, Lotta; Bixo, Marie; Hirschberg, Angelica Linden; Sundstrom-Poromaa, Inger; Hammar, Mats | 2010 | A randomized controlled study of taper-down or abrupt discontinuation of hormone therapy in women treated for vasomotor symptoms | Menopause (New York, N.Y.) |
| Mahon, Francois-Xavier; Rea, Delphine; Guilhot, Joelle; Guilhot, Francois; Huguet, Francoise; Nicolini, Franck; Legros, Laurence; Charbonnier, Aude; Guerci, Agnes; Varet, Bruno; Etienne, Gabriel; Reiffers, Josy; Rousselot, Philippe | 2010 | Discontinuation of imatinib in patients with chronic myeloid leukaemia who have maintained complete molecular remission for at least 2 years: the prospective, multicentre Stop Imatinib (STIM) trial | The Lancet. Oncology |
| Murata Y.; Kobayashi D.; Imuta N.; Haraguchi K.; Ieiri I.; Nishimura R.; Koyama S.; Mine K. | 2010 | Effects of the serotonin 1A, 2A, 2C, 3A, and 3B and serotonin transporter gene polymorphisms on the occurrence of paroxetine discontinuation syndrome | J. Clin. Psychopharmacol. |
| Oettl, Tobias; Zuliani, Eugenia; Gaspert, Ariana; Hopfer, Helmut; Dickenmann, Michael; Fehr, Thomas | 2010 | Late steroid withdrawal after ABO blood group-incompatible living donor kidney transplantation: high rate of mild cellular rejection | Transplantation |
| Oto, Maria; Espie, Colin A.; Duncan, Roderick | 2010 | An exploratory randomized controlled trial of immediate versus delayed withdrawal of antiepileptic drugs in patients with psychogenic nonepileptic attacks (PNEAs) | Epilepsia |
| Reimer C.; Bytzer P. | 2010 | Discontinuation of long-term proton pump inhibitor therapy in primary care patients: A randomized placebo-controlled trial in patients with symptom relapse | Eur. J. Gastroenterol. Hepatol. |
| Ross D.M.; Branford S.; Seymour J.F.; Schwarer A.P.; Arthur C.; Bartley P.A.; Slader C.; Field C.; Dang P.; Filshie R.J.; Mills A.K.; Grigg A.P.; Melo J.V.; Hughes T.P. | 2010 | Patients with chronic myeloid leukemia who maintain a complete molecular response after stopping imatinib treatment have evidence of persistent leukemia by DNA PCR | Leukemia |
| Saleem, Benazir; Keen, Helen; Goeb, Vincent; Parmar, Rekha; Nizam, Sharmin; Hensor, Elizabeth M. A.; Churchman, Sarah M.; Quinn, Mark; Wakefield, Richard; Conaghan, Philip G.; Ponchel, Frederique; Emery, Paul | 2010 | Patients with RA in remission on TNF blockers: when and in whom can TNF blocker therapy be stopped? | Annals of the rheumatic diseases |
| Takaku Y.; Nakagome K.; Kobayashi T.; Yamaguchi T.; Nishihara F.; Soma T.; Hagiwara K.; Kanazawa M.; Nagata M. | 2010 | Changes in airway inflammation and hyperresponsiveness after inhaled corticosteroid cessation in allergic asthma | Int. Arch. Allergy Immunol. |
| Tanaka, Y.; Takeuchi, T.; Mimori, T.; Saito, K.; Nawata, M.; Kameda, H.; Nojima, T.; Miyasaka, N.; Koike, T. | 2010 | Discontinuation of infliximab after attaining low disease activity in patients with rheumatoid arthritis: RRR (remission induction by Remicade in RA) study | Annals of the rheumatic diseases |
| Thamrin, Cindy; Taylor, D. Robin; Jones, Stuart L.; Suki, Bela; Frey, Urs | 2010 | Variability of lung function predicts loss of asthma control following withdrawal of inhaled corticosteroid treatment | Thorax |
| Thobois S.; Ardouin C.; Lhommée E.; Klinger H.; Lagrange C.; Xie J.; Fraix V.; Coelho Braga M.C.; Hassani R.; Kistner A.; Juphard A.; Seigneuret E.; Chabardes S.; Mertens P.; Polo G.; Reilhac A.; Costes N.; Lebars D.; Savasta M.; Tremblay L.; Quesada J.-L.; Bosson J.-L.; Benabid A.-L.; Broussolle E.; Pollak P.; Krack P. | 2010 | Non-motor dopamine withdrawal syndrome after surgery for Parkinson's disease: Predictors and underlying mesolimbic denervation | Brain |
| Tsunoda, Kenichi; Uchida, Hiroyuki; Suzuki, Takefumi; Watanabe, Koichiro; Yamashima, Tetsumori; Kashima, Haruo | 2010 | Effects of discontinuing benzodiazepine-derivative hypnotics on postural sway and cognitive functions in the elderly | International journal of geriatric psychiatry |
| Weber N.K.; Forman L.M.; Trotter J.F. | 2010 | HBIg discontinuation with maintenance oral anti-viral therapy and HBV vaccination in liver transplant recipients | Dig. Dis. Sci. |
| Boonstra, Geartsje; van Haren, Neeltje E. M.; Schnack, Hugo G.; Cahn, Wiepke; Burger, Huibert; Boersma, Maria; Kroon, Bart de; Grobbee, Diederick E.; Hulshoff Pol, Hilleke E.; Kahn, Renã© S. | 2011 | Brain volume changes after withdrawal of atypical antipsychotics in patients with first-episode schizophrenia | Journal of clinical psychopharmacology |
| Emoto Y.; Emoto H.; Oishi E.; Hikita S.; Wakakura M. | 2011 | Twelve cases of drug-induced blepharospasm improved within 2 months of psychotropic cessation | Drug Healthc. Patient Saf. |
| Heiwe, Susanne; Lönnquist, Ingeborg; Kã¤Llmã©N, Hã¥Kan | 2011 | Potential risk factors associated with risk for drop-out and relapse during and following withdrawal of opioid prescription medication | European Journal of Pain |
| Klieverik L.P.; Kalsbeek A.; Ackermans M.T.; Sauerwein H.P.; Wiersinga W.M.; Fliers E. | 2011 | Energy homeostasis and body weight before and after cessation of block and replacement therapy in euthyroid patients with graves' disease | Intl. J. Endocrinol. |
| Lenci, Ilaria; Tisone, Giuseppe; Di Paolo, Daniele; Marcuccilli, Fabio; Tariciotti, Laura; Ciotti, Marco; Svicher, Valentina; Perno, Carlo Federico; Angelico, Mario | 2011 | Safety of complete and sustained prophylaxis withdrawal in patients liver-transplanted for HBV-related cirrhosis at low risk of HBV recurrence | Journal of hepatology |
| Liesker, Jeroen J. W.; Bathoorn, Erik; Postma, Dirkje S.; Vonk, Judith M.; Timens, Wim; Kerstjens, Huib A. M. | 2011 | Sputum inflammation predicts exacerbations after cessation of inhaled corticosteroids in COPD | Respiratory medicine |
| Liu, Feng; Wang, Lei; Li, Xiao Ying; Liu, You De; Wang, Jing Bo; Zhang, Zhao Hua; Wang, Yao Zong | 2011 | Poor durability of lamivudine effectiveness despite stringent cessation criteria: a prospective clinical study in hepatitis B e antigen-negative chronic hepatitis B patients | Journal of gastroenterology and hepatology |
| McKellar G.E.; Hampson R.; Tierney A.; Capell H.A.; Madhok R. | 2011 | Nonsteroidal antiinflammatory drug withdrawal in patients with stable rheumatoid arthritis | J. Rheumatol. |
| Ogino, Shin; Miyamoto, Seiya; Tenjin, Tomomi; Kitajima, Rei; Ojima, Kazuaki; Miyake, Nobumi; Funamoto, Yasuyuki; Arai, Jun; Tsukahara, Sachiko; Ito, Yukie; Tadokoro, Masanori; Anai, Kiriko; Tatsunami, Shinobu; Kubota, Hiroshi; Kaneda, Yasuhiro; Yamaguchi, Noboru | 2011 | Effects of discontinuation of long-term biperiden use on cognitive function and quality of life in schizophrenia | Progress in neuro-psychopharmacology & biological psychiatry |
| Rathore, Chaturbhuj; Panda, Samhita; Sarma, P. Sankara; Radhakrishnan, Kurupath | 2011 | How safe is it to withdraw antiepileptic drugs following successful surgery for mesial temporal lobe epilepsy? | Epilepsia |
| Siger, Maå‚Gorzata; Durko, Agnieszka; Nicpan, Agnieszka; Konarska, Maria; Grudziecka, Monika; Selmaj, Krzysztof | 2011 | Discontinuation of interferon beta therapy in multiple sclerosis patients with high pre-treatment disease activity leads to prompt return to previous disease activity | Journal of the neurological sciences |
| Van De Wetering J.; Koumoutsakos P.; Peeters A.; Van Der Mast B.J.; De Kuiper P.; IJzermans J.N.; Weimar W.; Baan C.C. | 2011 | Discontinuation of calcineurin inhibitors treatment allows the development of FOXP3+ regulatory T-cells in patients after kidney transplantation | Clin. Transplant. |
| Vorma H.; Katila H. | 2011 | Effect of valproate on benzodiazepine withdrawal severity in opioid-dependent subjects: A pilot study | Heroin Addict. Relat. Clin. Probl. |
| Bergh, Sverre; Selbã¦K, Geir; Engedal, Knut | 2012 | Discontinuation of antidepressants in people with dementia and neuropsychiatric symptoms (DESEP study): Double blind, randomised, parallel group, placebo controlled trial | BMJ: British Medical Journal |
| Campbell, James D.; Moore, David; Degerman, Richard; Kaharuza, Frank; Were, Willy; Muramuzi, Emmy; Odongo, George; Wetaka, Milton; Mermin, Jonathan; Tappero, Jordan W. | 2012 | HIV-infected ugandan adults taking antiretroviral therapy with CD4 counts 200 cells/muL who discontinue cotrimoxazole prophylaxis have increased risk of malaria and diarrhea | Clinical infectious diseases : an official publication of the Infectious Diseases Society of America |
| Evenepoel P.; Sprangers B.; Lerut E.; Bammens B.; Claes K.; Kuypers D.; Meijers B.; Vanrenterghem Y. | 2012 | Mineral metabolism in renal transplant recipients discontinuing cinacalcet at the time of transplantation: A prospective observational study | Clin. Transplant. |
| Faber G.; Smid H.G.O.M.; Van Gool A.R.; Wiersma D.; Van Den Bosch R.J. | 2012 | The effects of guided discontinuation of antipsychotics on neurocognition in first onset psychosis | Eur. Psychiatry |
| Frost M.L.; Siddique M.; Blake G.M.; Moore A.E.; Marsden P.K.; Schleyer P.J.; Eastell R.; Fogelman I. | 2012 | Regional bone metabolism at the lumbar spine and hip following discontinuation of alendronate and risedronate treatment in postmenopausal women | Osteoporosis Int |
| Louis E.; Mary J.-Y.; Verniermassouille G.; Grimaud J.-C.; Bouhnik Y.; Laharie D.; Dupas J.L.; Pillant H.; Picon L.; Veyrac M.; Flamant M.; Savoye G.; Jian R.; Devos M.; Porcher R.; Paintaud G.; Piver E.; Colombel J.-F.; Lemann M. | 2012 | Maintenance of remission among patients with Crohn's disease on antimetabolite therapy after infliximab therapy is stopped | Gastroenterology |
| Mourer, Jacqueline S.; Ewe, See Hooi; Mallat, Marko J. K.; Ng, Arnold C. T.; Rabelink, Ton J.; Bax, Jeroen J.; Delgado, Victoria; Fijter, Johan W. de | 2012 | Late calcineurin inhibitor withdrawal prevents progressive left ventricular diastolic dysfunction in renal transplant recipients | Transplantation |
| Ramirez, Claudia; Vargas, Guadalupe; Gonzalez, Baldomero; Grossman, Ashley; Rabago, Julia; Sosa, Ernesto; Espinosa-de-Los-Monteros, Ana Laura; Mercado, Moises | 2012 | Discontinuation of octreotide LAR after long term, successful treatment of patients with acromegaly: is it worth trying? | European journal of endocrinology / European Federation of Endocrine Societies |
| Scandling J.D.; Busque S.; Dejbakhsh-Jones S.; Benike C.; Sarwal M.; Millan M.T.; Shizuru J.A.; Lowsky R.; Engleman E.G.; Strober S. | 2012 | Tolerance and withdrawal of immunosuppressive drugs in patients given kidney and hematopoietic cell transplants | Am. J. Transplant. |
| van der Maas, Aatke; Kievit, Wietske; van den Bemt, Bart J F; van den Hoogen, Frank H J; van Riel, Piet L.; den Broeder, Alfons A. | 2012 | Down-titration and discontinuation of infliximab in rheumatoid arthritis patients with stable low disease activity and stable treatment: an observational cohort study | Annals of the rheumatic diseases |
| Azermai, M.; Petrovic, M.; Engelborghs, S.; Elseviers, M. M.; van der Mussele, S.; Debruyne, H.; van Bortel, L.; Vander Stichele, R. H. | 2013 | The effects of abrupt antipsychotic discontinuation in cognitively impaired older persons: a pilot study | Aging & mental health |
| Benítez C.; Londoño M.-C.; Miquel R.; Manzia T.-M.; Abraldes J.G.; Lozano J.-J.; Martínez-Llordella M.; López M.; Angelico R.; Bohne F.; Sese P.; Daoud F.; Larcier P.; Roelen D.L.; Claas F.; Whitehouse G.; Lerut J.; Pirenne J.; Rimola A.; Tisone G.; Sánchez-Fueyo A. | 2013 | Prospective multicenter clinical trial of immunosuppressive drug withdrawal in stable adult liver transplant recipients | HEPATOLOGY |
| Cantarovich, D.; Hodemon-Corne, B.; Trebern-Launay, K.; Giral, M.; Foucher, Y.; Dantan, E. | 2013 | Early steroid withdrawal compared with steroid avoidance correlates with graft failure among kidney transplant recipients with an history of diabetes | Transplantation proceedings |
| Chaiwarith R.; Praparattanapan J.; Nuntachit N.; Kotarathitithum W.; Supparatpinyo K. | 2013 | Discontinuation of primary and secondary prophylaxis for opportunistic infections in HIV-Infected Patients Who Had CD4+ Cell Count <200 cells/mm3 but Undetectable Plasma HIV-1 RNA: An Open-Label Randomized Controlled Trial | AIDS Patient Care STDs |
| De Kuijper G.; Mulder H.; Evenhuis H.; Visser F.; Hoekstra P.J. | 2013 | Effects of controlled discontinuation of long-term used antipsychotics on weight and metabolic parameters in individuals with intellectual disability | J. Clin. Psychopharmacol. |
| Faulhaber, Marion; Mading, Ilona; Malehsa, Doris; Raggi, Matthias C.; Haverich, Axel; Bara, Christoph L. | 2013 | Steroid withdrawal and reduction of cyclosporine A under mycophenolate mofetil after heart transplantation | International immunopharmacology |
| Hajjar I.; Hart M.; Wan S.-H.; Novak V. | 2013 | Safety and blood pressure trajectory of short-term withdrawal of antihypertensive medications in older adults: Experience from a clinical trial sample | J. Am. Soc. Hypertens. |
| Hirata S.; Saito K.; Kubo S.; Fukuyo S.; Mizuno Y.; Iwata S.; Nawata M.; Sawamukai N.; Nakano K.; Yamaoka K.; Tanaka Y. | 2013 | Discontinuation of adalimumab after attaining disease activity score 28-erythrocyte sedimentation rate remission in patients with rheumatoid arthritis (HONOR study): An observational study | Arthritis Res. Ther. |
| Jackson T.P.; Lonergan D.F.; Todd R.D.; Martin P.R. | 2013 | Intentional Intrathecal Opioid Detoxification in 3 Patients: Characterization of the Intrathecal Opioid Withdrawal Syndrome | Pain Pract |
| Kim, Young Jip; Kim, Kichan; Hwang, Sun Hyuk; Kim, Soon Sun; Lee, Dami; Cheong, Jae Youn; Cho, Sung Won | 2013 | Durability after discontinuation of nucleos(t)ide therapy in chronic HBeAg negative hepatitis patients | Clinical and molecular hepatology |
| Krumova, Elena K.; Bennemann, Philipp; Kindler, Doris; Schwarzer, Andreas; Zenz, Michael; Maier, Christoph | 2013 | Low pain intensity after opioid withdrawal as a first step of a comprehensive pain rehabilitation program predicts long-term nonuse of opioids in chronic noncancer pain | The Clinical journal of pain |
| Kuijper, Gerda de; Mulder, Hans; Evenhuis, Heleen; Visser, Frank; Hoekstra, Pieter J. | 2013 | Effects of controlled discontinuation of long-term used antipsychotics on weight and metabolic parameters in individuals with intellectual disability | Journal of clinical psychopharmacology |
| Lee S.-E.; Choi S.Y.; Bang J.-H.; Kim S.-H.; Jang E.-J.; Byeun J.-Y.; Park J.-E.; Jeon H.-R.; Oh Y.J.; Kim H.-J.; Kim Y.-K.; Park J.S.; Jeong S.H.; Zang D.Y.; Oh S.; Koo D.H.; Kim H.; Do Y.R.; Kwak J.-Y.; Kim J.-A.; Kim D.-Y.; Mun Y.-C.; Mauro M.J.; Kim D.-W. | 2013 | Predictive factors for successful imatinib cessation in chronic myeloid leukemia patients treated with imatinib | Am. J. Hematol. |
| Mizoguchi I.; Yoshimoto T.; Katagiri S.; Mizuguchi J.; Tauchi T.; Kimura Y.; Inokuchi K.; Ohyashiki J.H.; Ohyashiki K. | 2013 | Sustained upregulation of effector natural killer cells in chronic myeloid leukemia after discontinuation of imatinib | Cancer Sci |
| Mourer J.S.; De Koning E.J.P.; Van Zwet E.W.; Mallat M.J.K.; Rabelink T.J.; De Fijter J.W. | 2013 | Impact of late calcineurin inhibitor withdrawal on ambulatory blood pressure and carotid intima media thickness in renal transplant recipients | Transplantation |
| Ross D.M.; Branford S.; Seymour J.F.; Schwarer A.P.; Arthur C.; Yeung D.T.; Dang P.; Goyne J.M.; Slader C.; Filshie R.J.; Mills A.K.; Melo J.V.; White D.L.; Grigg A.P.; Hughes T.P. | 2013 | Safety and efficacy of imatinib cessation for CML patients with stable undetectable minimal residual disease: Results from the TWISTER study | Blood |
| Su L.; Di Q.; Yu N.; Zhang Y. | 2013 | Predictors for relapse after antiepileptic drug withdrawal in seizure-free patients with epilepsy | J. Clin. Neurosci. |
| Thielen, Noortje; van der Holt, Bronno; Cornelissen, Jan J.; Verhoef, Gregor E. G.; Gussinklo, Titia; Biemond, Bart J.; Daenen, Simon M. G.; Deenik, Wendy; van Marwijk Kooy, Rien; Petersen, Eefke; Smit, Willem M.; Valk, Peter J. M.; Ossenkoppele, Gert J.; Janssen, Jeroen J W M | 2013 | Imatinib discontinuation in chronic phase myeloid leukaemia patients in sustained complete molecular response: a randomised trial of the Dutch-Belgian Cooperative Trial for Haemato-Oncology (HOVON) | European journal of cancer (Oxford, England : 1990) |
| Tsuge M.; Murakami E.; Imamura M.; Abe H.; Miki D.; Hiraga N.; Takahashi S.; Ochi H.; Hayes C.N.; Ginba H.; Matsuyama K.; Kawakami H.; Chayama K. | 2013 | Serum HBV RNA and HBeAg are useful markers for the safe discontinuation of nucleotide analogue treatments in chronic hepatitis B patients | J. Gastroenterol. |
| Weimar C.; Cotton D.; Sha N.; Sacco R.L.; Bath P.M.W.; Weber R.; Diener H.C. | 2013 | Discontinuation of antiplatelet study medication and risk of recurrent stroke and cardiovascular events: Results from the PRoFESS study | Cerebrovasc. Dis. |
| Winkle, Roger A.; Mead, R. Hardwin; Engel, Gregory; Kong, Melissa H.; Patrawala, Rob A. | 2013 | Discontinuing anticoagulation following successful atrial fibrillation ablation in patients with prior strokes | Journal of interventional cardiac electrophysiology : an international journal of arrhythmias and pacing |
| Ardissino G.; Testa S.; Possenti I.; Tel F.; Paglialonga F.; Salardi S.; Tedeschi S.; Belingheri M.; Cugno M. | 2014 | Discontinuation of eculizumab maintenance treatment for atypical hemolytic uremic syndrome: A report of 10 cases | AM. J. KIDNEY DIS. |
| Bourgeois J.; Elseviers M.M.; Van Bortel L.; Petrovic M.; Stichele R.H.V. | 2014 | Feasibility of discontinuing chronic benzodiazepine use in nursing home residents: A pilot study | Eur. J. Clin. Pharmacol. |
| Chavarri-Guerra Y.; Higgins M.J.; Szymonifka J.; Cigler T.; Liedke P.; Partridge A.; Ligibel J.; Come S.E.; Finkelstein D.; Ryan P.D.; Goss P.E. | 2014 | Drug withdrawal in women with progressive metastatic breast cancer while on aromatase inhibitor therapy | Br. J. Cancer |
| De Kuijper G.; Evenhuis H.; Minderaa R.B.; Hoekstra P.J. | 2014 | Effects of controlled discontinuation of long-term used antipsychotics for behavioural symptoms in individuals with intellectual disability | J. Intellect. Disabil. Res. |
| Desmarais J.E.; Beauclair L.; Annable L.; Bélanger M.-C.; Kolivakis T.T.; Margolese H.C. | 2014 | Effects of discontinuing anticholinergic treatment on movement disorders, cognition and psychopathology in patients with schizophrenia | Ther. Adv. Psychopharmacol. |
| Djukanovic, N.; Todorovic, Z.; Obradovic, S.; Njegomirovic, S.; Zamaklar-Trifunovic, D.; Protiä‡, D.; Ostojic, M. | 2014 | Clopidogrel cessation triggers aspirin rebound in patients with coronary stent | J CLIN PHARM THER (Journal of Clinical Pharmacy & Therapeutics) |
| Gafoor V.A.; Saifudheen K.; Jose J. | 2014 | Recurrence rate of seizure following discontinuation of anti-epileptic drugs in patients with normal long term electroencephalography | Ann. Indian Acad. Neurol. |
| Gueguen, Antoine; Roux, Pascal; Deschamps, Romain; Moulignier, Antoine; Bensa, Caroline; Savatovsky, Julien; Heran, Franã§Oise; Gout, Olivier | 2014 | Abnormal inflammatory activity returns after natalizumab cessation in multiple sclerosis | Journal of Neurology, Neurosurgery & Psychiatry |
| Kim, Mi Na; Lee, Chun Kyon; Ahn, Sang Hoon; Lee, Sangheun; Kim, Seung Up; Kim, Do Young; Kim, Hyon Suk; Han, Kwang-Hyub; Chon, Chae Yoon; Park, Jun Yong | 2014 | Maintaining remission in lamivudine-resistant patients with a virological response to adefovir add-on lamivudine after stopping lamivudine therapy | Liver international : official journal of the International Association for the Study of the Liver |
| Kuijper, G. de; Evenhuis, H.; Minderaa, R. B.; Hoekstra, P. J. | 2014 | Effects of controlled discontinuation of long-term used antipsychotics for behavioural symptoms in individuals with intellectual disability | Journal of intellectual disability research : JIDR |
| Lähteenmäki R.; Puustinen J.; Vahlberg T.; Lyles A.; Neuvonen P.J.; Partinen M.; Räihä I.; Kivelä S.-L. | 2014 | Melatonin for sedative withdrawal in older patients with primary insomnia: A randomized double-blind placebo-controlled trial | Br. J. Clin. Pharmacol. |
| Lee S.-H.; Kwon H.-S.; Park Y.-M.; Ko S.-H.; Choi Y.-H.; Yoon K.-H.; Ahn Y.-B. | 2014 | Statin discontinuation after achieving a target low density lipoprotein cholesterol level in type 2 diabetic patients without cardiovascular disease: A randomized controlled study | Diabetes Metab. J. |
| Lemos K.F.; Rabelo-Silva E.R.; Ribeiro L.W.; Cruz L.N.; Polanczyk C.A. | 2014 | Effect of nitrate withdrawal on quality of life and adherence to treatment in patients with stable angina: Evidence from a randomized clinical trial | Coron. Artery Dis. |
| Li W.; Si Y.; Zou X.-M.; An D.-M.; Yang H.; Zhou D. | 2014 | Prospective study on the withdrawal and reinstitution of antiepileptic drugs among seizure-free patients in west China | J. Clin. Neurosci. |
| Lin, Victor Chia-Hsiang; Liao, Chun-Hou; Kuo, Hann-Chorng | 2014 | Progression of lower urinary tract symptoms after discontinuation of 1 medication from 2-year combined alpha-blocker and 5-alpha-reductase inhibitor therapy for benign prostatic hyperplasia in men--a randomized multicenter study | Urology |
| Modi S.; Tripathi M.; Saha S.; Goswami R. | 2014 | Seizures in patients with idiopathic hypoparathyroidism: Effect of antiepileptic drug withdrawal on recurrence of seizures and serum calcium control | Eur. J. Endocrinol. |
| Molander P.; Färkkilä M.; Salminen K.; Kemppainen H.; Blomster T.; Koskela R.; Jussila A.; Rautiainen H.; Nissinen M.; Haapamäki J.; Arkkila P.; Nieminen U.; Kuisma J.; Punkkinen J.; Kolho K.-L.; Mustonen H.; Sipponen T. | 2014 | Outcome after discontinuation of TNFα-blocking therapy in patients with inflammatory bowel disease in deep remission | Inflammatory Bowel Dis |
| Nishimoto N.; Amano K.; Hirabayashi Y.; Horiuchi T.; Ishii T.; Iwahashi M.; Iwamoto M.; Kohsaka H.; Kondo M.; Matsubara T.; Mimura T.; Miyahara H.; Ohta S.; Saeki Y.; Saito K.; Sano H.; Takasugi K.; Takeuchi T.; Tohma S.; Tsuru T.; Ueki Y.; Yamana J.; Hashimoto J.; Matsutani T.; Murakami M.; Takagi N. | 2014 | Drug free REmission/low disease activity after cessation of tocilizumab (Actemra) Monotherapy (DREAM) study | Mod. Rheumatol. |
| Ory-Magne F.; Corvol J.-C.; Azulay J.-P.; Bonnet A.-M.; Brefel-Courbon C.; Damier P.; Dellapina E.; Destée A.; Durif F.; Galitzky M.; Lebouvier T.; Meissner W.; Thalamas C.; Tison F.; Salis A.; Sommet A.; Viallet F.; Vidailhet M.; Rascol O. | 2014 | Withdrawing amantadine in dyskinetic patients with Parkinson disease | Neurology |
| Prodam, Flavia; Savastio, Silvia; Genoni, Giulia; Babu, Deepak; Giordano, Mara; Ricotti, Roberta; Aimaretti, Gianluca; Bona, Gianni; Bellone, Simonetta | 2014 | Effects of growth hormone (GH) therapy withdrawal on glucose metabolism in not confirmed GH deficient adolescents at final height | PloS one |
| Puustinen J.; Lähteenmäki R.; Polo-Kantola P.; Salo P.; Vahlberg T.; Lyles A.; Neuvonen P.J.; Partinen M.; Räihä I.; Kivelä S.-L. | 2014 | Effect of withdrawal from long-term use of temazepam, zopiclone or zolpidem as hypnotic agents on cognition in older adults | Eur. J. Clin. Pharmacol. |
| Rossi, Andrea; Guerriero, Massimo; Corrado, Antonio | 2014 | Withdrawal of inhaled corticosteroids can be safe in COPD patients at low risk of exacerbation: a real-life study on the appropriateness of treatment in moderate COPD patients (OPTIMO) | Respiratory research |
| Rousselot, Philippe; Charbonnier, Aude; Cony-Makhoul, Pascale; Agape, Philippe; Nicolini, Franck E.; Varet, Bruno; Gardembas, Martine; Etienne, Gabriel; Rea, Delphine; Roy, Lydia; Escoffre-Barbe, Martine; Guerci-Bresler, Agnes; Tulliez, Michel; Prost, Stephane; Spentchian, Marc; Cayuela, Jean Michel; Reiffers, Josy; Chomel, Jean Claude; Turhan, Ali; Guilhot, Joelle; Guilhot, Francois; Mahon, Francois-Xavier | 2014 | Loss of major molecular response as a trigger for restarting tyrosine kinase inhibitor therapy in patients with chronic-phase chronic myelogenous leukemia who have stopped imatinib after durable undetectable disease | Journal of clinical oncology : official journal of the American Society of Clinical Oncology |
| Sangalli F.; Moiola L.; Ferrè L.; Radaelli M.; Barcella V.; Rodegher M.; Colombo B.; Martinelli Boneschi F.; Martinelli V.; Comi G. | 2014 | Long-term management of natalizumab discontinuation in a large monocentric cohort of multiple sclerosis patients | Mult. Scler. Relat. Disord. |
| Suzuki H.; Inoue Y.; Mikami K.; Gen K. | 2014 | The influence and changes in the dosages of concomitantly used psychotropic drugs associated with the discontinuation of donepezil in severe Alzheimer's disease with behavioral and psychological symptoms on dementia: A preliminary open-label trial | Ther. Adv. Psychopharmacol. |
| Tveito, M.; Lorentzen, B.; Engedal, K.; Tanum, L.; Bramness, J. G.; Refsum, H.; Hã¸Iseth, G. | 2014 | Changes in cognitive function during psychogeriatric treatment in relation to benzodiazepine cessation | Pharmacopsychiatry |
| Vicens C.; Bejarano F.; Sempere E.; Mateu C.; Fiol F.; Socias I.; Aragonès E.; Palop V.; Beltran J.L.; Piñol J.L.; Lera G.; Folch S.; Mengual M.; Basora J.; Esteva M.; Llobera J.; Roca M.; Gili M.; Leiva A. | 2014 | Comparative efficacy of two interventions to discontinue long-term benzodiazepine use: Cluster randomised controlled trial in primary care | Br. J. Psychiatry |
| Vilar, Lucio; Fleseriu, Maria; Naves, Luciana Ansaneli; Albuquerque, Jose Luciano; Gadelha, Patricia Sampaio; dos Santos Faria, Manuel; Nascimento, Gilvan Cortes; Montenegro, Renan Magalhaes [JR]; Montenegro, Renan Magalhaes | 2014 | Can we predict long-term remission after somatostatin analog withdrawal in patients with acromegaly? Results from a multicenter prospective trial | Endocrine |
| Wunsch E.; Trottier J.; Milkiewicz M.; Raszeja-Wyszomirska J.; Hirschfield G.M.; Barbier O.; Milkiewicz P. | 2014 | Prospective evaluation of ursodeoxycholic acid withdrawal in patients with primary sclerosing cholangitis | HEPATOLOGY |
| Yamaguchi, Kunihisa; Izaki, Hirofumi; Takahashi, Masayuki; Fukumori, Tomoharu; Nishitani, Masaaki; Sutou, Yasushi; Uema, Kenzou; Kawano, Akira; Hamao, Takumi; Kanayama, Hiro-omi | 2014 | Changes in levels of prostate-specific antigen and testosterone following discontinuation of long-term hormone therapy for non-metastatic prostate cancer | The journal of medical investigation : JMI |
| Young, J.; Wang, Q.; Fux, C. A.; Bernasconi, E.; Furrer, H.; Vernazza, P.; Calmy, A.; Cavassini, M.; Weber, R.; Battegay, M.; Bucher, H. C. | 2014 | The rate of recovery in renal function when patients with HIV infection discontinue treatment with tenofovir | HIV medicine |
| Araujo E.G.; Finzel S.; Englbrecht M.; Schreiber D.A.; Faustini F.; Hueber A.; Nas K.; Rech J.; Schett G. | 2015 | High incidence of disease recurrence after discontinuation of disease-modifying antirheumatic drug treatment in patients with psoriatic arthritis in remission | Ann. Rheum. Dis. |
| Ballard, Clive; Thomas, Alan; Gerry, Stephen; Yu, Ly-Mee; Aarsland, Dag; Merritt, Claire; Corbett, Anne; Davison, Christopher; Sharma, Narenda; Khan, Zunera; Creese, Byron; Loughlin, Paul; Bannister, Carol; Burns, Alistair; Win, Soe Nyunt; Walker, Zuzana | 2015 | A double-blind randomized placebo-controlled withdrawal trial comparing memantine and antipsychotics for the long-term treatment of function and neuropsychiatric symptoms in people with Alzheimer's disease (MAIN-AD) | Journal of the American Medical Directors Association |
| Bernat A.L.; Oyama K.; Hamdi S.; Mandonnet E.; Vexiau D.; Pocard M.; George B.; Froelich S. | 2015 | Growth stabilization and regression of meningiomas after discontinuation of cyproterone acetate: a case series of 12 patients | Acta Neurochir |
| Boers-Sonderen, Marye J.; Desar, Ingrid M. E.; Futterer, Jurgen J.; Mulder, Sasja F.; Geus-Oei, Lioe-Fee de; Mulders, Peter F.; Van Der Graaf, Winette T A; Oyen, Wim J. G.; Van Herpen, Carla M L | 2015 | Biological Effects After Discontinuation of VEGFR Inhibitors in Metastatic Renal Cell Cancer | Anticancer research |
| Browne G.A.; Griffin T.P.; O'Shea P.M.; Dennedy M.C. | 2015 | β-Blocker withdrawal is preferable for accurate interpretation of the aldosterone-renin ratio in chronically treated hypertension | Clin. Endocrinol. |
| Buti M.; Casillas R.; Riveiro-Barciela M.; Homs M.; Tabernero D.; Salcedo M.T.; Rodriguez-Frias F.; Esteban R. | 2015 | Tenofovir discontinuation after long-term viral suppression in HBeAg negative chronic hepatitis B. Can HBsAg levels be useful? | J. Clin. Virol. |
| Coura-Filho G.B.; Willegaignon J.; Buchpiguel C.A.; Sapienza M.T. | 2015 | Effects of Thyroid Hormone Withdrawal and Recombinant Human Thyrotropin on Glomerular Filtration Rate during Radioiodine Therapy for Well-Differentiated Thyroid Cancer | THYROID |
| Fernández I.; Loinaz C.; Hernández O.; Abradelo M.; Manrique A.; Calvo J.; Manzano M.; García A.; Cambra F.; Castellano G.; Jiménez C. | 2015 | Tenofovir/entecavir monotherapy after hepatitis B immunoglobulin withdrawal is safe and effective in the prevention of hepatitis B in liver transplant recipients | Transplant Infect. Dis. |
| Hirano K.; Tada M.; Isayama H.; Sasahira N.; Umefune G.; Akiyama D.; Watanabe T.; Saito T.; Takagi K.; Takahara N.; Hamada T.; Mizuno S.; Miyabayashi K.; Mohri D.; Kogure H.; Yamamoto N.; Nakai Y.; Arizumi T.; Toda N.; Koike K. | 2015 | Outcome of Long-term Maintenance Steroid Therapy Cessation in Patients With Autoimmune Pancreatitis: A Prospective Study | J. Clin. Gastroenterol. |
| Hooten, W. Michael; Warner, David O. | 2015 | Varenicline for opioid withdrawal in patients with chronic pain: A randomized, single-blinded, placebo controlled pilot trial | Addictive behaviors |
| Lin N.-C.; Wang H.-K.; Yeh Y.-C.; Liu C.-P.; Loong C.-C.; Tsai H.-L.; Chen C.-Y.; Chin T.; Liu C. | 2015 | Minimization or withdrawal of immunosuppressants in pediatric liver transplant recipients | J. PEDIATR. SURG. |
| Lossius M.I.; Nakken K.O.; Mowinckel P.; Taubøll E.; Gjerstad L. | 2015 | Favorable change of lipid profile after carbamazepine withdrawal | Acta Neurol. Scand. |
| Moonen J.E.F.; Foster-Dingley J.C.; Ce Ruijter W.; Van Der Grond J.; Bertens A.S.; Van Buchem M.A.; Gussekloo J.; Middelkoop H.A.; Wermer M.J.H.; Westendorp R.G.J.; De Craen A.J.M.; Van Der Mast R.C. | 2015 | Effect of discontinuation of antihypertensive treatment in elderly people on cognitive functioning-the DANTE Study Leiden: A randomized clinical trial | JAMA Intern. Med. |
| Moschos M.M.; Nitoda E.; Chatziralli I.P.; Gatzioufas Z.; Koutsandrea C.; Kitsos G. | 2015 | Assessment of hydroxychloroquine maculopathy after cessation of treatment: An optical coherence tomography and multifocal electroretinography study | Drug Des. Dev. Ther. |
| Moverley A.; Coates L.; Marzo-Ortega H.; Waxman R.; Torgerson D.; Cocks K.; Watson J.; Helliwell P.S. | 2015 | A feasibility study for a randomised controlled trial of treatment withdrawal in psoriatic arthritis (REmoval of treatment for patients in REmission in psoriatic ArThritis (RETREAT (F)) | Clin. Rheumatol. |
| Seto W.-K.; Hui A.J.; Wong V.W.-S.; Wong G.L.-H.; Liu K.S.-H.; Lai C.-L.; Yuen M.-F.; Chan H.L.-Y. | 2015 | Treatment cessation of entecavir in Asian patients with hepatitis B e antigen negative chronic hepatitis B: A multicentre prospective study | Gut |
| Srivanichakorn W.; Sriwijitkamol A.; Kongchoo A.; Sriussadaporn S.; Plengvidhya N.; Lertwattanarak R.A.; Vannasaeng S.; Thongtang N. | 2015 | Withdrawal of sulfonylureas from patients with type 2 diabetes receiving long-term sulfonylurea and insulin combination therapy results in deterioration of glycemic control: A randomized controlled trial | Diabetes Metab. Syndr. Obes. Targets Ther. |
| Takeuchi, Tsutomu; Matsubara, Tsukasa; Ohta, Shuji; Mukai, Masaya; Amano, Koichi; Tohma, Shigeto; Tanaka, Yoshiya; Yamanaka, Hisashi; Miyasaka, Nobuyuki | 2015 | Biologic-free remission of established rheumatoid arthritis after discontinuation of abatacept: a prospective, multicentre, observational study in Japan | Rheumatology (Oxford, England) |
| van Herwaarden, Noortje; van der Maas, Aatke; Minten, Michiel J. M.; van den Hoogen, Frank H J; Kievit, Wietske; van Vollenhoven, Ronald F.; Bijlsma, Johannes W. J.; van den Bemt, Bart J F; den Broeder, Alfons A. | 2015 | Disease activity guided dose reduction and withdrawal of adalimumab or etanercept compared with usual care in rheumatoid arthritis: open label, randomised controlled, non-inferiority trial | BMJ (Clinical research ed.) |
| Wenzl, Heimo H.; Primas, Christian; Novacek, Gottfried; Teml, Alexander; Offerlbauer-Ernst, Anna; Hogenauer, Christoph; Vogelsang, Harald; Petritsch, Wolfgang; Reinisch, Walter | 2015 | Withdrawal of long-term maintenance treatment with azathioprine tends to increase relapse risk in patients with Crohn's disease | Digestive diseases and sciences |
| Gasparini S.; Ferlazzo E.; Giussani G.; Italiano D.; Cianci V.; Sueri C.; Spina E.; Beghi E.; Aguglia U. | 2016 | Rapid versus slow withdrawal of antiepileptic monotherapy in 2-year seizure-free adult patients with epilepsy (RASLOW) study: a pragmatic multicentre, prospective, randomized, controlled study | Neurol. Sci. |
| Haschka J.; Englbrecht M.; Hueber A.J.; Manger B.; Kleyer A.; Reiser M.; Finzel S.; Tony H.-P.; Kleinert S.; Feuchtenberger M.; Fleck M.; Manger K.; Ochs W.; Schmitt-Haendle M.; Wendler J.; Schuch F.; Ronneberger M.; Lorenz H.-M.; Nuesslein H.; Alten R.; Demary W.; Henes J.; Schett G.; Rech J. | 2016 | Relapse rates in patients with rheumatoid arthritis in stable remission tapering or stopping antirheumatic therapy: Interim results from the prospective randomised controlled RETRO study | Ann. Rheum. Dis. |
| Van Vollenhoven R.F.; Østergaard M.; Leirisalo-Repo M.; Uhlig T.; Jansson M.; Larsson E.; Brock F.; Franck-Larsson K. | 2016 | Full dose, reduced dose or discontinuation of etanercept in rheumatoid arthritis | Ann. Rheum. Dis. |
| Baandrup, Lone; Lindschou, Jane; Winkel, Per; Gluud, Christian; Glenthoj, Birte Y. | 2016 | Prolonged-release melatonin versus placebo for benzodiazepine discontinuation in patients with schizophrenia or bipolar disorder: A randomised, placebo-controlled, blinded trial | World J Biol Psychiatry (The World Journal of Biological Psychiatry) |
| Nina Djukanovic, Zoran Todorovic, Danijela Zamaklar-Trifunovic, Dragana Protic, Boris Dzudovic, Miodrag Ostojic, Slobodan Obradovic | 2016 | Sustained increase in platelet aggregation after the cessation of clopidogrel | Clinical and Experimental Pharmacology and Physiology |
|  |  |  |  |
| Marjan Ghiti Moghadam; Harald E. Vonkeman; Peter M. ten  Klooster; Janneke Tekstra; Dirkjan van Schaardenburg; Mirian  Starmans-Kool; Elisabeth Brouwer; Reinhard Bos; Willem  F. Lems; Edgar M. Colin; Cornelia F. Allaart; Inger L.  Meek; Robert Landewé; Hein J. Bernelot Moens; Piet  L.C.M. van Riel; Mart A.F.J. van de Laar; Tim L. Jansen;  on behalf of the Dutch National POET Collaboration. | 2016 | Stopping Tumor Necrosis Factor-inhibitors in Patients with Established Rheumatoid  Arthritis in Remission or Stable Low Disease Activity: A Pragmatic Randomized Multicenter  Open-Label Controlled Trial. | Arthritis & Rheumatology |
| Nathan Herrmann, Jordana O’Regan, Myuri Ruthirakuhan,  Alexander Kiss, Goran Eryavec, Evelyn Williams,  Krista L. Lanctôt | 2016 | A Randomized Placebo-Controlled Discontinuation Study  of Cholinesterase Inhibitors in Institutionalized Patients  With Moderate to Severe Alzheimer Disease | JAMDA |
| KenjiHirano, Minoru Tada, Hiroyuki Isayama,  Naoki Sasahira, Gyotane Umefune, Dai Akiyama,  Takeo Watanabe, Tomotaka Saito, Kaoru Takagi,  Naminatsu Takahara, Tsuyoshi Hamada,  Suguru Mizuno,Koji Miyabayashi,Dai Mohri,  Hirofumi Kogure, Natsuyo Yamamoto,  Yousuke Nakai,Toshihiko Arizumi,  Nobuo Toda, and Kazuhiko Koike | 2016 | Outcome of Long-term Maintenance Steroid Therapy  Cessation in Patients With Autoimmune Pancreatitis  A Prospective Study | J Clin Gastroenterol |
| Michele Iudici & Serena Vettori & Barbara Russo & Veronica Giacco &  Domenico Capocotta & Gabriele Valentini | 2016 | Outcome of a glucocorticoid discontinuation regimen in patients  with inactive systemic sclerosis | Clin Rheumatol |
| Simona Malucchi, Marco Capobianco, Alessia di Sapio, Marianna Lo Re, Paola Cavalla and Antonio Bertolotto | 2016 | Rituximab suppresses disease activity after  natalizumab withdrawal: an exploratory  study | Multiple Sclerosis and Demyelinating Disorders |
| Jacqueline Mayoral-van Son; Victor Ortiz-Garcia de la Foz,; Obdulia Martinez-Garcia in nursing; Teresa Moreno; Maria Parrilla-Escobar; Elsa M. Valdizan; and Benedicto Crespo-Facorro | 2016 | Mayoral-Van Son J.; Ortiz-Garcia De La Foz V.; Martinez-Garcia O.; Moreno T.; Parrilla-Escobar M.; Valdizan E.M.; Crespo-Facorro B. (2016): Clinical outcome after antipsychotic treatment discontinuation in functionally recovered first-episode nonaffective psychosis individuals: A 3-year naturalistic follow-up study. In: The Journal of clinical psychiatry 77 (4), S. 492–500. DOI: 10.4088/JCP.14m09540. | The Journal of clinical psychiatry |
| Marianne O. Price, Amanda Scanameo, BS, Matthew T. Feng, Francis W. Price, Jr. | 2016 | Risk of Immunologic Rejection Episodes after Discontinuing  Topical Corticosteroids | Ophthalmology |
| Ada C. Stefanescu Schmidt, Dean J. Kereiakes, Donald E. Cutlip, Robert W. Yeh, Ralph B. D’Agostino Sr., Joseph M. Massaro, Wen-Hua Hsieh, and Laura Mauri | 2017 | Myocardial Infarction Risk after Discontinuation of Thienopyridine Therapy in the Randomized DAPT Study | Circulation. |
| Fatih Karakaya,Sevil Özer ,Çağdaş Kalkan ,E Ali Tüzün ,Aysun Çalışkan,Onur Keskin ,Gökhan Kabaçam , Senem Karatayl , Ersin Karatayli ,A Mithat Bozdayi ,Ramazan Idilman,Cihan Yurdaydin | 2017 | Discontinuation of lamivudine treatment in HbeAg-negative chronic hepatitis B: A pilot study with longterm follow-up | Antivir Ther |
| T Ishida, S Yoshida, Y Kimura, Y Fujiki, T Kotani, T Takeuchi, S Makino and S Arawaka | 2018 | Efficacy of discontinuing risedronate for patients with systemic  lupus erythematosus: a prospective study | Lupus |
| Luis Jara-Palomares, Aurora Solier-Lopez, Teresa Elias-Hernandez, Maria Isabel Asensio-Cruz, Isabel Blasco-Esquivias,  Veronica Sanchez-Lopez, Maria Rodriguez de la Borbolla, Elena Arellano-Orden, Lionel Suarez-Valdivia, Samira Marin-Romero,  Lucia Marin-Barrera, Aranzazu Ruiz-Garcia, Emilio Montero-Romero, Silvia Navarro-Herrero, Jose Luis Lopez-Campos,  Maria Pilar Serrano-Gotarredona, Juan Manuel Praena-Fernandez, Jose Maria Sanchez-Diaz and Remedios Otero-Candelera | 2018 | D-dimer and high-sensitivity C-reactive protein levels to  predict venous thromboembolism recurrence after  discontinuation of anticoagulation for cancer-associated  thrombosis | British Journal of Cancer |
| Daniel J. Lovell, Anne L. Johnson, Bin Huang, Beth S. Gottlieb, Paula W. Morris, Yukiko Kimura, Karen Onel, Suzanne C. Li, Alexei A. Grom, Janalee Taylor, Hermine I. Brunner, Jennifer L. Huggins, James J. Nocton, Kathleen A. Haines, Barbara S. Edelheit, Michael Shishov, Lawrence K. Jung, Calvin B. Williams, Melissa S. Tesher, Denise M. Costanzo, Lawrence S. Zemel, Jason A. Dare, Murray H. Passo, Kaleo C. Ede, Judyann C. Olson, Elaine A. Cassidy, Thomas A. Griffin, Linda Wagner-Weiner, Jennifer E. Weiss, Larry B. Vogler, Kelly A. Rouster-Stevens, Timothy Beukelman,, Randy Q. Cron, Daniel Kietz, Kenneth Schikler, Jay Mehta, Tracy V. Ting, James W. Verbsky, B. Anne Eberhard, MBBS, Steven Spalding, Chen Chen, and Edward H. Giannini | 2018 | Risk, Timing and Predictors of Disease Flare after Discontinuation of Anti-Tumor Necrosis Factor (TNF) Therapy in Children with Polyarticular Forms of Juvenile Idiopathic Arthritis (JIA) in Clinical Inactive Disease | Arthritis Rheumatol. |
| Matteo A. Maninia, Gavin Whitehouse, Matthew Bruce, Matteo Passerini,Tiong Y. Lim, Ivana Carey, Aisling Considine, Pietro Lampertico, Abid Suddlea,Nigel Heaton, Michael Heneghan, Kosh Agarwal | 2018 | Entecavir or tenofovir monotherapy prevents HBV recurrence in livertransplant recipients: A 5-year follow-up study after hepatitis Bimmunoglobulin withdrawal | Digestive and Liver Disease |
| Masaya Okada,1 Jun Imagawa,2 Hideo Tanaka,3 Hirohisa Nakamae,4  Masayuki Hino,4 Kazunori Murai,5 Yoji Ishida,5 Takashi Kumagai,6 Seiichi Sato,7  Kazuteru Ohashi,8 Hisashi Sakamaki,8 Hisashi Wakita,9 Nobuhiko Uoshima,10  Yasunori Nakagawa,11 Yosuke Minami,12 Masahiro Ogasawara,13  Tomoharu Takeoka, Hiroshi Akasaka, Takahiko Utsumi, Naokuni Uike,  Tsutomu Sato, Sachiko Ando,Kensuke Usuki, Syuichi Mizuta,  Satoshi Hashino, Tetsuhiko Nomura, Masato Shikami, Hisashi Fukutani,  Yokiko Ohe, Hiroshi Kosugi, Hirohiko Shibayama, Yasuhiro Maeda,  Toshihiro Fukushima, Hirohito Yamazaki, Kazuo Tsubaki,  Toshimasa Kukita, Yoko Adachi, Toshiki Nataduka, Hiroto Sakoda,  Hisayuki Yokoyama, Takahiro Okamoto, Yukari Shirasugi, Yasushi Onishi | 2018 | Final 3-year Results of the Dasatinib  Discontinuation Trial in Patients With Chronic  Myeloid Leukemia Who Received Dasatinib as a  Second-line Treatment | Clinical Lymphoma, Myeloma & Leukemia |
| Lotte Ramerman, Pieter J. Hoekstra, and  Gerda de Kuijper | 2018 | Changes in Health-Related Quality of Life  in People With Intellectual Disabilities Who  Discontinue Long-Term Used Antipsychotic  Drugs for Challenging Behaviors | The Journal of Clinical Pharmacology |
| Marcelo De Rosa1, Francisco Azzato1, Jorge E. Toblli2, Graciela De Rosa1, Federico Fuentes1,  Haikady N. Nagaraja3, Ryan Nash4 and Brad H. Rovin5 | 2018 | A prospective observational cohort study  highlights kidney biopsy findings of lupus  nephritis patients in remission who flare  following withdrawal of maintenance therapy | Kidney International |
| Susanne Saussele, Johan Richter, Joelle Guilhot, Franz X Gruber, Henrik Hjorth-Hansen, Antonio Almeida, Jeroen J W M Janssen, Jiri Mayer,  Perttu Koskenvesa, Panayiotis Panayiotidis, Ulla Olsson-Strömberg, Joaquin Martinez-Lopez, Philippe Rousselot, Hanne Vestergaard,  Hans Ehrencrona, Veli Kairisto, Katerina Machová Poláková, Martin C Müller, Satu Mustjoki, Marc G Berger, Alice Fabarius,  Wolf-Karsten Hofmann, Andreas Hochhaus, Markus Pfirrmann, Francois-Xavier Mahon, on behalf of the EURO-SKI investigators | 2018 | Discontinuation of tyrosine kinase inhibitor therapy in  chronic myeloid leukaemia (EURO-SKI): a prespecified interim  analysis of a prospective, multicentre, non-randomised, trial | Lancet Oncol |
| Chung-Cheng Wang Yung-Hong Jiang Hann-Chorng Kuo | 2018 | Higher urge severity score predicts resumption of overactive  bladder (OAB) medication following discontinuation of  mirabegron treatment in patients with OAB | Lower Urinary Tract Symptoms |
| Asai S.; Hayashi M.; Hanabayashi M.; Kanayama Y.; Takemoto T.; Yabe Y.; Shioura T.; Ishikawa H.; Yoshioka Y.; Kato T.; Hirano Y.; Fujibayashi T.; Hattori Y.; Kobayakawa T.; Ando M.; Kuwatsuka Y.; Takahashi N.; Matsumoto T.; Asai N.; Sobue Y.; Nishiume T.; | 2019 | Discontinuation of concomitant methotrexate in Japanese patients with rheumatoid arthritis treated with tocilizumab: An interventional study | Modern rheumatology |
| Browne, Gerard A.; Griffin, Tomas P.; O'Shea, Paula M.; Dennedy, Michael Conall | 2019 | b-Blocker withdrawal is preferable for accurate interpretation of  the aldosterone–renin ratio in chronically treated hypertension | Clinical Endocrinology |
| Richard E Clark, Fotios Polydoros, Jane F Apperley, Dragana Milojkovic, Katherine Rothwell, Christopher Pocock, Jennifer Byrne,  Hugues de Lavallade, Wendy Osborne, Lisa Robinson, Stephen G O’Brien, Lucy Read, Letizia Foroni, Mhairi Copland | 2019 | De-escalation of tyrosine kinase inhibitor therapy before  complete treatment discontinuation in patients with  chronic myeloid leukaemia (DESTINY): a non-randomised,  phase 2 trial | Lancet Haematol |
| Constance H. Fung, Jennifer L. Martin, Cathy Alessi, Joseph M. Dzierzewski ,  Ian A. Cook, Alison Moore, Austin Grinberg , Michelle Zeidler and Lara Kierlin | 2019 | Hypnotic Discontinuation Using a Blinded (Masked) Tapering Approach: A Case Series | Front. Psychiatry |
| Tadakazu Hisamatsu  Shingo Kato  Reiko Kunisaki  Minoru Matsuura  Masakazu Nagahori  Satoshi Motoya  Motohiro Esaki  Norimasa Fukata  Satoko Inoue  Takeshi Sugaya  Hirotake Sakuraba  Fumihito Hirai  Kenji Watanabe  Takanori Kanai  Makoto Naganuma  Hiroshi Nakase  Yasuo Suzuki  Mamoru Watanabe  Toshifumi Hibi  Masanori Nojima  Takayuki Matsumoto | 2019 | Withdrawal of thiopurines in Crohn’s disease treated  with scheduled adalimumab maintenance: a prospective  randomised clinical trial (DIAMOND2) | J Gastroenterol |
| Vadim Jucaud; Abraham Shaked; Michele DesMarais; Peter Sayre;  Sandy Feng; Josh Levitsky; Matthew J. Everly | 2019 | Prevalence and impact of de novo DSA during a multicenter immunosuppression withdrawal trial in adult liver transplant recipients | Hepatology |
| M. Kerkhof· J. A. F. Koekkoek· M. J. Vos M. J. van den Bent W. Taal T. J. Postma J. E. C. Bromberg·  M. C. M. Kouwenhoven L. Dirven J. C. Reijneveld M. J. B. Taphoorn | 2019 | Withdrawal of antiepileptic drugs in patients with low grade  and anaplastic glioma after long-term seizure freedom: a prospective  observational study | Journal of Neuro-Oncology |
| Kun-Peng Li, Jing-Yu Jin, Jin-Shui Yang, Yan Li, Wei Zhao, Gui Luo, Jian Zhu, Jiang-Lin Zhang,  Feng Huang | 2019 | Full dose, half dose, or discontinuation of etanercept  biosimilar in early axial spondyloarthritis patients:  a real-world study in China | Arch Med Sci |
| Anne-Flore M. Matthijssen., Andrea Dietrich, Margreet Bierens., Renee Kleine Deters.,  Gigi H.H. van de Loo-Neus., Barbara J. van den Hoofdakker, Jan K. Buitelaar,  Pieter J. Hoekstra | 2019 | Continued Benefits of Methylphenidate in ADHD  After 2 Years in Clinical Practice: A Randomized  Placebo-Controlled Discontinuation Study | AJP in Advance |
| Ronan Roussel Santiago Duran-García Yilong Zhang  Suneri Shah Carolyn Darmiento MS R. Ravi Shankar  Gregory T. Golm Raymond L. H. Lam \| Edward A. O'Neill \|  Ira Gantz Keith D. Kaufman Samuel S. Engel | 2019 | Double-blind, randomized clinical trial comparing the efficacy  and safety of continuing or discontinuing the dipeptidyl  peptidase-4 inhibitor sitagliptin when initiating insulin glargine  therapy in patients with type 2 diabetes: The CompoSIT-I  Study | Diabetes Obes Metab. |
| Halumatha Surendra Shree Shilpa, N. Naveen Kumar, Eswaran Maheswari,  Harave Shanmugam Virupaksha, Viswam Subeesh, Ganesan Rajalekshmi Saraswathy,  Radhika Kunnavil | 2019 | Deprescribing of benzodiazepines and Z-drugs amongst the psychiatric  patients of a tertiary care Hospital | Asian Journal of Psychiatry |
| Kiyohiko Takahashi, Kyu Yong Cho, Akinobu Nakamura , Aika Miya , Arina Miyoshi, Chiho Yamamoto,  Hiroshi Nomoto, Hirokatsu Niwa, Kiyohito Takahashi, Naoki Manda, Yoshio Kurihara, Shin Aoki, Yoichi M Ito,  Tatsuya Atsumi, Hideaki Miyoshi | 2019 | Should sulfonylurea be discontinued or  maintained at the lowest dose when starting  ipragliflozin? A multicenter observational study  in Japanese patients with type 2 diabetes | J Diabetes Investig |
| Olivier Vittecoq, Sandra Desouches, Marie Kozyreff, Julia Nicolau,  Sophie Pouplin,1 Pascal Rottenberg, Nicolas Sens, Thierry Lequerre,  Gilles Avenel | 2019 | Relapse in rheumatoid arthritis patients  undergoing dose reduction and  withdrawal of biologics: are predictable  factors more relevant than predictive  parameters? An observational  prospective real-life  study | BMJ Open |
| Mesut Yilmaz and Sermin Guven Mese | 2019 | Durable response after discontinuation  of nivolumab therapy in the absence of  disease progression or toxicity with two  advanced NSCLC patients | J Oncol Pharm Practice |
| Yaryna Boyko, Viktoriia Ivanova, Maryna Vakaruk, Tamila Kozina, Nataliia Shevchenko,  Nataliia Vaizer, Olha Synoverska, Oksana Chubata, Olha Marchuk, Anna Havrylyuk | 2020 | Blood calprotectin in children with juvenile idiopathic arthritis:  relationship to flare development after discontinuation of treatment | Reumatologia 2020 |
| Jacqueline Kyosiimire-Lugemwa,, Zacchaeus Anywaine, Andrew Abaasa, Jonathan Levin, Ben Gombe, Kenneth Musinguzi, Pontiano Kaleebu,  Heiner Grosskurth, Paula Munderi and Pietro Pala | 2020 | Effect of Stopping Cotrimoxazole Preventive Therapy  on Microbial Translocation and Inflammatory Markers  Among Human Immunodeficiency Virus–Infected  Ugandan Adults on Antiretroviral Therapy: The COSTOP  Trial Immunology Substudy | The Journal of Infectious Diseases |
| Mathian, A.Micheline Pha, Julien Haroche, Fleur Cohen-Aubart,  Miguel Hié, Marc Pineton de Chambrun, Thi Huong Du Boutin, Makoto Miyara,  Guy Gorochov, Hans Yssel, Patrick Cherin, Hervé Devilliers, Zahir Amoura | 2020 | Withdrawal of low-dose  prednisone in SLE patients  with a clinically quiescent disease for more than 1  year: a randomised clinical trial | Ann Rheum Dis |
